# Intermittent Preventive Treatment in Infants for the Prevention of Malaria in Rural Western Kenya: A Randomized, Double-Blind Placebo-Controlled Trial

**DOI:** 10.1371/journal.pone.0010016

**Published:** 2010-04-02

**Authors:** Frank O. Odhiambo, Mary J. Hamel, John Williamson, Kim Lindblade, Feiko O. ter Kuile, Elizabeth Peterson, Peter Otieno, Simon Kariuki, John Vulule, Laurence Slutsker, Robert D. Newman

**Affiliations:** 1 Kenya Medical Research Institute, Centre for Global Health Research, Kisumu, Kenya; 2 Malaria Branch, Division of Parasitic Diseases, Centers for Disease Control and Prevention, Atlanta, Georgia, United States of America; 3 Liverpool School of Tropical Medicine, Liverpool, United Kingdom; 4 Vermont Department of Health, Burlington, Vermont, United States of America; The George Washington University Medical Center, United States of America

## Abstract

**Background:**

Intermittent preventive treatment in infants (IPTi) with sulphadoxine-pyrimethamine (SP) for the prevention of malaria has shown promising results in six trials. However, resistance to SP is rising and alternative drug combinations need to be evaluated to better understand the role of treatment versus prophylactic effects.

**Methods:**

Between March 2004 and March 2008, in an area of western Kenya with year round malaria transmission with high seasonal intensity and high usage of insecticide-treated nets, we conducted a randomized, double-blind placebo-controlled trial with SP plus 3 days of artesunate (SP-AS3), 3 days of amodiaquine-artesunate (AQ3-AS3), or 3 days of short-acting chlorproguanil-dapsone (CD3) administered at routine expanded programme of immunization visits (10 weeks, 14 weeks and 9 months).

**Principal Findings:**

1,365 subjects were included in the analysis. The incidence of first or only episode of clinical malaria during the first year of life (primary endpoint) was 0.98 episodes/person-year in the placebo group, 0.74 in the SP-AS3 group, 0.76 in the AQ3-AS3 group, and 0.82 in the CD3 group. The protective efficacy (PE) and 95% confidence intervals against the primary endpoint were: 25.7% (6.3, 41.1); 25.9% (6.8, 41.0); and 16.3% (−5.2, 33.5) in the SP-AS3, AQ3-AS3, and CD3 groups, respectively. The PEs for moderate-to-severe anaemia were: 27.5% (−6.9, 50.8); 23.1% (−11.9, 47.2); and 11.4% (−28.6, 39.0). The duration of the protective effect remained significant for up to 5 to 8 weeks for SP-AS3 and AQ3-AS3. There was no evidence for a sustained beneficial or rebound effect in the second year of life. All regimens were well tolerated.

**Conclusions:**

These results support the view that IPTi with long-acting regimens provide protection against clinical malaria for up to 8 weeks even in the presence of high ITN coverage, and that the prophylactic rather than the treatment effect of IPTi appears central to its protective efficacy.

**Trial Registration:**

ClinicalTrials.gov NCT00111163

## Introduction

It is estimated that approximately 100 million children aged <5 years in Africa live in areas where malaria transmission occurs; each year >800,000 die from the direct effects of malaria [1], [2]. Acute febrile illnesses, mostly attributable to malaria, are responsible for 400–900 million hospitalizations per year among African children [3]. Malaria-related anaemia is widespread [4] and its burden falls primarily on infants and children <5 y ears of age[ 5], [6].

In 2001, Schellenberg and colleagues in Tanzania demonstrated that intermittent preventive treatment in infants (IPTi) with sulphadoxine-pyrimethamine (SP) administered at routine Expanded Program of Immunization (EPI) visits with iron supplementation from 2–6 months of age reduced the incidence of clinical malaria and severe anaemia by 59% (95% CI: 41, 72; p<0.0001) and 50% (95% CI: 8, 73; p = 0.023) respectively in the first year of life [7]. Another study of IPTi conducted contemporaneously in northern Tanzania also reported significant reductions in malarial fevers and severe anaemia (65% [95% CI: 42, 77; p<0.001] and 71% [95% CI: 39, 87; p = 0.001] respectively) using 3 presumptive treatment courses of amodiaquine (AQ) given at 60 day intervals over 6 months starting at 12–16 weeks of age [8].

Although currently available evidence regarding the efficacy of IPTi with SP is promising [7], [9]–[14], the protective efficacy of IPTi with SP in more recent trials has been more modest than the initial Tanzania trial, and a recent study reported no protective efficacy of IPTi with SP [15]. In addition, *Plasmodium falciparum* resistance to SP is high in sub-Saharan Africa [16], [17], and alternative drugs for this strategy need to be identified. The mechanism by which IPTi works is not well understood: it is unknown whether the intermittent clearance of existing malaria infections (treatment effect) or the post-treatment prophylactic effect of long-acting drugs is more important [18]. It remains uncertain for how long the protective effect of IPTi persists and whether there is any evidence for a sustained benefit after the immediate effect of the drugs have waned [19], [20]. There is some evidence from a recent study showing that the prophylactic effect of the study drug used was central to the mode of action of IPTi [21]. A better understanding of the mode of action of IPTi is a key factor in determining potentially suitable alternative drugs.

We conducted a randomized, double-blind placebo-controlled trial to evaluate the efficacy and safety of IPTi with regimens containing short and long-acting anti-malarial drugs administered at routine EPI vaccinations (at 10, 14 weeks and 9 months). This was a proof of concept study to understand the importance for IPTi of the treatment versus prophylactic effect. The trial was conducted in a rural area of western Kenya with year round malaria transmission with high seasonal intensity, high SP treatment failure rates [17] and high usage of insecticide-treated nets (ITNs). This study was one of a series of trials coordinated by the IPTi Consortium (www.ipti-malaria.org) [22], [23]; this study aimed to evaluate the efficacy of artemisinin-based combination drugs as alternatives to the use of SP alone for IPTi.

## Methods

### Study Area

The study took place in Asembo (Rarieda District, Nyanza Province), western Kenya, where approximately 55,000 persons live in 76 villages over 178 square kilometres. The characteristics of the area have been described in detail elsewhere [24], [25]. This area around Lake Victoria once experienced intense perennial malaria transmission marked by seasonal variation [26]. However, transmission rates were reduced by 90% during an ITN efficacy trial (ITN coverage for children <5 years of age was 83% in 2002) conducted from 1996 to 2002 [27], [28]. The continued provision of free ITNs to the entire population has helped to maintain the low Entomological Inoculation Rate (EIR), currently estimated at 7 infective bites per person per year [24], [28]. Despite the reduced transmission, cross-sectional surveys conducted in 1999, 2000 and 2001 reported the prevalence of parasitaemia and severe anaemia (Haemoglobin [Hb] <7 g/dL) among children <5 years of age as 36% and 10%, respectively [28]. The infant mortality rate in the study area in 2002 was 125/1,000 live births in [24]. EPI coverage in the study area is relatively low: 55% of children receive all three doses of the diphtheria-tetanus toxoid-pertussis-hepatitis B-*Haemophilus influenza* type b vaccine (PENT) and oral polio vaccine (OPV), 48% receive the measles vaccine and only 38% receive all the essential vaccines in the programme [29].

SP became the first-line anti-malarial drug in Kenya in 1998. Data from 1999–2000 demonstrated that the Adequate Clinical and Parasitological Response (ACPR) by day 28 for SP was 54% among children aged <5 years in neighbouring Bondo District, western Kenya [17]. In 2000, the treatment response by day 28 to amodiaquine (AQ) mono-therapy in symptomatic children <5 years of age was reported as 89% in Bungoma, western Kenya [16]. A more recent study conducted in Bondo in 2007, reported similarly high (90%) ACPR for amodiaquine-artesunate by day 28 [30]. The available data on treatment response to chlorproguanil-dapsone showed 96% ACPR by day 14, obtained by pooling data from trials conducted in five African countries in 2000; no data were available from western Kenya at the start of the study [31]. In 2004, during the course of this trial, the Kenya Ministry of Health (MOH) adopted artemether-lumefantrine (AL) as the first-line treatment for uncomplicated malaria [32]; implementation began in late 2006.

### Study Participants

The protocol for this trial and supporting CONSORT checklist are available as supporting information; see Checklist S1 and Protocol S1. Figure S1 depicts the trial time-line for participants. *Inclusion criteria*: infants aged 5 to 16 weeks who were resident in Asembo (study area), when they attended a Mother and Child Health clinic prior to their first OPV/PENT vaccination at any one of the 4 study clinics located in 3 large outpatient healthcare units and 1 mission hospital within the study area were offered enrolment in the study. Infants were enrolled from within a 3 km radius of the given health facility as this distance was considered convenient enough to allow re-visits. **Exclusion criteria.** Infants with known allergy to any of the study drugs, receiving cotrimoxazole prophylaxis for opportunistic infections, suffering concomitant illness requiring hospitalization or transfusion, or planning to be away from the study area for more than 6 months were excluded. The study was explained to parents or guardians of eligible infants and written informed consent was obtained; see Consent S1. Comprehension of the consent form was assessed orally using a standard set of questions. The study was conducted according to Good Clinical Practice guidelines and monitored by a Data Safety and Monitoring Board (DSMB). The protocol was approved by the National Ethical Review Committee of the Kenya Medical Research Institute (KEMRI) in Nairobi, and the Institutional Review Board of the Centers for Disease Control and Prevention in Atlanta, Georgia. The study is registered with ClinicalTrials.gov (NCT00111163).

### Randomization, Allocation Concealment and Blinding

Study arm assignment was done by permuted block randomization with a block size of 8. Each study drug combination was assigned to a colour and then packed into identical bottles labelled only with the colour, the course number (1, 2, or 3), the day of treatment (day 1, day 2, or day 3), and whether the drug was drug A or B of the combination. Labelling was performed by an independent scientist who was not otherwise involved in this trial. Doses of study drugs were prepared from these bottles in an isolated and locked pharmacy room in each study clinic. The list of study identification numbers linked to a given colour was kept locked and was accessible solely to the pharmaceutical technician preparing doses. The colour-arm assignment of the study identification numbers remained concealed to everyone except the technician. The technician did not have access to names of participants. The key to the colour-arm assignment was kept by the DSMB. The key was obtained in exchange for the locked dataset and detailed analytical plan.

### Study Drug Selection

For the short-acting drug, chlorproguanil-dapsone (CD) was selected based on demonstrated efficacy in Kenya [31], [33]–[35] and northern Tanzania [36]. CD has a terminal elimination half-life (t_½_) of between 6 and 19 hours [33]. However, since March 2008 CD has no longer been available. The two long-acting anti-malarials given in combination with short-acting artesunate were AQ and SP. Amodiaquine had been shown to be efficacious for IPTi in neighbouring Tanzania [8], and for treatment in western Kenya [16]. The desethyl metabolite of AQ, which accounts for nearly all anti-malarial activity, has a t_½_ of between 3 to 12 days [37]. Sulphadoxine has a t_½_ of 7 days, whereas pyrimethamine has a t_½_ of 3 days [38]. Artesunate (AS) was added to both SP and AQ because of uncertainties of how rising *P. falciparum* resistance to SP and AQ alone might compromise efficacy if the primary action of IPTi was due to parasite clearance. Available data suggest that AQ plus AS is more efficacious than AQ alone in the treatment of uncomplicated malaria in symptomatic African children [39], [40]. Similarly, SP plus AS is more efficacious than SP alone [17], [41]–[43]. Artesunate is very short-acting and has a t_½_ of approximately 1 hour [18], and was not added to CD because of the very high treatment success rate observed with CD in Kenya and northern Tanzania, and because at the time there were no published studies on the safety or efficacy of a CD plus AS combination.

### Study drug regimens

The study drug regimens were as follows: (i) SP-AS3 (DAFRA Pharma, Belgium): one SP half-strength tablet (250 mg sulphadoxine, 12.5 mg pyrimethamine) once on the first day of treatment (followed by a placebo SP tablet on days 2 and 3) and one paediatric artesunate tablet (25 mg) once daily for 3 days; (ii) AQ3-AS3 (DAFRA Pharma, Belgium): one paediatric amodiaquine tablet (67.5 mg), once daily for 3 days and one paediatric artesunate tablet (25 mg) once daily for 3 days; (iii) CD3 (GlaxoSmithKline, United Kingdom): one paediatric caplet (15 mg chlorproguanil and 18.75 mg of dapsone) once daily for 3 days administered with a placebo once daily for 3 days; and (iv) Placebo (DAFRA Pharma, Belgium): 2 placebo tablets co-administered once daily for 3 days. The doses given were the same for all 3 courses of IPTi. All study drugs were Good Manufacturing Practice certified.

To increase palatability, all medications were dispensed as crushed tablets mixed with pharmaceutical-grade syrup (Humco Corporation, Texas, USA) immediately prior to administration in an opaque syringe. The first dose of each course of study drug was administered and supervised at the healthcare unit by a study nurse; subsequent doses of each course were administered and supervised at home by study staff. All the infants were observed for 30 minutes after drug administration; if vomiting occurred during that period a repeat dose was administered and supervised at the healthcare unit by a study nurse. If vomiting occurred during home administered doses, the child was immediately referred to the healthcare unit, where a repeat dose was administered. Supplies of iron sulphate (2 mg/kg/day) (Laboratory and Allied Ltd., Kenya) were given at the first and second IPTi courses, and 1 month later at the fourth scheduled visit to the parent/guardian of study children for home administration during a 4-month period from 2.5 to 6.5 months of age.

### Study Procedures

Malaria incidence was estimated through passive surveillance. Infants with any illnesses were instructed to present to one of the 4 study clinics for care, which was provided free of charge. A rapid diagnostic test (RDT) (OptiMal®, DiaMed, Switzerland) for malaria was performed for all infants with a documented fever (≥37.5°C by axillary measurement), history of recent fever in the previous 48 hours, or evidence of pallor. Results of the RDT were used solely for clinical management and not for trial outcome measures. If the RDT was positive, the infant was treated with quinine (7-day course) or AL (for children ≥12 months of age weighing >5 kg). If the sick visit and positive RDT occurred at the 10- or 14-week or 9-month visit when study drug was due, then IPTi and a paired 7-day treatment (prepared for each arm prior to trial commencement) was given such that the arms containing active study drugs were paired with placebo, whereas the placebo arm was paired with quinine. This arrangement ensured that all participants regardless of study arm received appropriate treatment for clinical malaria. In addition, Hb levels were checked and blood slides prepared. Any other childhood illnesses were treated according to the Integrated Management of Childhood Illnesses and MOH guidelines.

Participants who presented with serious adverse events suspected to be attributable to study drugs were withdrawn from receiving further study drugs, but follow-up visits and healthcare continued. Those who migrated outside the study area for more than 3 months were suspended from the study but allowed to re-enter upon returning to the study area, or were considered lost to follow-up if they failed to return.

### Laboratory Methods

Thick blood films prepared from capillary blood were stained with 10% Giemsa stain (pH 7.2) for 15 minutes and examined for parasites. Parasites and leukocytes were counted in the same fields until 500 leukocytes were counted. Due to difficulties in obtaining sufficient quantities of blood from infants, the logistics of carrying out complete blood counts in the field or even using accurate blood volumes (10 µL) to make blood smears, we preferred to use the WHO recommended method of assumed leukocyte counts in such areas, also used in some IPTi studies [11], [44]. Even though this method could potentially result in inaccurate estimates of the parasite densities it would not introduce a bias across study arms. Parasite densities were estimated using an assumed leukocyte count of 8,000 leukocytes per µL of blood. All slides were read by two laboratory technicians independently, who were blinded to treatment arms. Slides with discrepant readings or with parasite density estimations that differed by >50% were sent to a senior technician for a third reading, the result of which was considered final. All laboratory technicians were trained at the KEMRI and Walter Reed Project Centre for Excellence in Microscopy in Kisumu. Capillary blood was collected in micro-cuvettes and examined in Hemocue® photometers (Angelholm, Sweden) to determine Hb levels. All Hemocue® photometers were verified against known standard micro-cuvettes weekly and sent monthly for cleaning, maintenance, and verification against a Coulter Counter® (Beckman Coulter, USA). Capillary blood was also collected at 12 months of age in tubes containing ethylene diamine tetra acetic acid (EDTA) for sickle cell status determination using a standard Hb electrophoresis technique. Samples for glucose-6-phosphate dehydrogenase genotypes (G6PD) were analysed after all study subjects had completed the primary follow-up period (12 months) using blood samples collected at enrolment. G6PD mutations at nucleotides 376 (A – G) and 202 (G – A) were detected by polymerase chain reaction followed by digestion with restriction endonucleases *Fok*I and *Nla*III respectively [45].

### Outcomes

The primary outcome was time to the first or only episode of clinical malaria in the first year of life. An episode of clinical malaria was defined as an axillary temperature of at least 37.5°C or history of fever in the preceding 48 hours together with asexual *P. falciparum* parasitaemia of any density. Percent of febrile episodes attributable to malaria, cut-off parasite densities, and sensitivity and specificity of the cut-off densities for this site have been described elsewhere [46].

Secondary outcomes (time to event) were; moderate-to-severe anaemia (defined as Hb <8 g/dL) and all-cause hospitalizations in the first year of life, as defined in the protocol. In addition, other secondary outcomes included high density clinical malaria (defined as clinical malaria with parasite densities >5,000/µL of blood [time to event]); mild anaemia (defined as Hb <11 g/dL [time to event]); multiple episodes of clinical malaria (number of events over follow-up time); all-cause outpatient sick visits (time to event); hospitalizations with malaria (time to event); and the incidence of clinical malaria was also assessed to determine the duration of effect (number of events over follow-up time) and any rebound effect in the second year of life (e.g. increase in malaria incidence following discontinuation of IPTi). All additional outcomes were pre-specified before analysis; some were included in a separate analysis plan because we felt they were clinically important indicators, whereas others were included in order to facilitate comparability with other IPTi studies. The analysis plan was drawn up after the protocol was written (before analysis) to be comparable to other IPTi trials; see Analysis Plan S1 and Protocol S1.

### Sample Size

Preliminary sample size calculations were conducted for the outcome time to first episode of clinical malaria in the first 18 months of age and were based on the log-rank test. Median time in the placebo group receiving iron was assumed to be 18 months (168 weeks after the first intervention visit) [28]. The sample size required for 90% power to detect a hazard ratio of 0.6 (protective efficacy [PE] OF 40%, PE defined as [1- hazard ratio] ×100%) assuming alpha  = 0.017 to correct for three pair wise comparisons was 1000 infants (250 per arm). Adjusting for 10% loss to follow-up, 13% mortality and 11% of infants contracting malaria before the first treatment regimen resulted in a sample size of 1,516 infants (379 per arm).

### Data Management and Statistical Methods

Data were entered using scan-ready Teleforms® and an optical scanner (Cardiff, California, USA). Data were checked for internal consistency and out-of-range values. Study subjects were enrolled 4 weeks prior to receiving the first intervention, and were subsequently excluded from analysis if diagnosed with clinical malaria or they died or out-migrated from the study area within that 4-week period. This modified ITT population included all the participants who received the first course of study drug regardless of whether they received all or part of the interventions. We used Cox regression models to estimate the risk of the first or only episode of clinical malaria during the period starting from the first intervention visit and ending at 1 year of age or at censoring due to withdrawal or death [47]. The models' assumption regarding the proportionality of the hazard ratio was analyzed by assessing the interaction between age and effect of treatment with a time-dependent Cox regression model. Kaplan-Meier curves were used to plot the time to event analyses. Secondary analyses were performed to assess the effect of non-compliance on the efficacy estimates derived from the primary analysis. Only those who had received all three doses of IPTi or placebo within 28 days of the scheduled intervention visits were included in these per-protocol analyses. Multiple malaria episodes and hospital admissions were assessed using Generalised Estimating Equations (GEE) Poisson regression models to take into account intra-individual correlation [48]. During the 28 days following treatment for an episode of clinical malaria, participants were considered not at risk for a new episode of malaria, and any positive blood smear during that time period was considered a recrudescence. Furthermore, if a subject migrated outside the study area for 3 or more consecutive months at any given time and returned prior to the end of the trial, it would be reported by a Compliance Monitor, and they would be included in the multiple episodes analysis but considered not at risk for the period of their absences.

Post-dose analyses were done: firstly, by fixing a Cox regression model to look at the primary endpoint within a 30day period after each course of IPTi; and secondly, by using biweekly time versus treatment interaction models excluding the first IPTi course because the period of post-treatment prophylaxis overlapped with the second IPTi course of treatment. A Lexis expansion [49] was used to obtain estimates within defined time strata at 14 day intervals after IPTi courses 2 and 3 by fitting a Poisson regression model with time versus treatment interaction, and using GEE to take into account intra-individual correlation. Multiple episodes of clinical malaria were assessed, and PEs calculated within each consecutive period of 14 days after each of the relevant courses of IPTi. Infants were excluded from the analysis for a period of 28 days after each event. The Poisson model was pooled across the respective IPTi courses allowing for a more robust estimation of PEs over time. Biweekly intervals were preferred for analysis because some weekly intervals had very few episodes of clinical malaria or lacked them altogether. Anthropometric indices were calculated using growth references from the National Center for Health Services, USA and the World Health Organization [50]. The analysis was performed using SAS version 9.1.3 (SAS Institute, Cary, North Carolina, USA), and STATA version 10 (STATA Corporation, College Station, Texas, USA).

## Results

### Participant and Baseline Characteristics

Between March 2004 and March 2008, 1,365 (90.0%) of 1,516 randomized infants received ≥1 dose of study drug and were included in the ITT analysis. Fifty-five (4.0%) out of the 1,365 died, 38 (2.8%) withdrew consent, and 191 (14.0%) were lost to follow-up due to migration; 1,081 (79.2%) completed the 1-year follow-up period (Figure S2). The four study groups were comparable with regard to sex, Hb genotype, G6PD phenotype, weight-for-age, age at first, second, and third IPTi courses, use of ITNs, and compliance with iron supplementation during the first year of life (table 1).

**Table 1:** Characteristics of IPTi study groups in western Kenya.

Parameter	Placebo	SP-AS3	AQ3-AS3	CD3
	(n = 337)	(n = 339)	(n = 347)	(n = 342)
**Male sex** [%]	46.9	53.1	53.9	50.6
**Hb genotype**†:				
AA [%]	75.3	76.1	78.4	76.7
AS ″	23.3	22.9	18.9	21.0
SS ″	0.4	0.4	0.7	0.4
Other ″	1.1	0.7	2.1	2.0
**G6PD deficiency**:				
Normal [%]	73.9	71.6	68.0	72.2
Mild-deficient ″	12.5	15.4	13.3	11.4
Deficient ″	13.6	13.0	18.7	16.4
**Age [months]-1^st^ IPTi** (mean±sd)	2.7±0.4	2.7±0.4	2.7±0.5	2.7±0.5
**Age [months]-2^nd^ IPTi** ″	3.7±0.5	3.7±0.5	3.7±0.6	3.7±0.6
**Age [months]-3^rd^ IPTi** ″	9.2±0.3	9.2±0.3	9.2±0.3	9.2±0.3
**Weight-for-age z score – 1^st^ IPTi** ″	0.3±1.1	0.3±1.1	0.3±1.1	0.3±1.1
**ITN use-night before 1^st^ IPTi** [%]	77.7	76.6	75.6	74.0
<67% iron compliance*[%]	16.0	16.7	17.9	15.8

Note: *at the end of iron supplementation (6.5 months of age), compliance measured as percentage of iron taken over the expected; †at 1 year of age; [%] figure is presented as a percentage.

### Protective Efficacy (PE) against main Outcomes

The incidence of clinical malaria between the first dose of IPTi and 12 months of age was 0.98 episodes per person-year in the placebo group and 0.74, 0.76, and 0.82 in the SP-AS3, AQ3-AS3, and CD3 groups, respectively. The PE against the first or only episode of clinical malaria was 25.7% (95% CI: 6.3, 41.1; p = 0.012) in the SP-AS3 group and 25.9% (95% CI: 6.8, 41.0; p = 0.01) in the AQ3-AS3 group, when compared with placebo. When multiple episodes of clinical malaria were considered the results were similar: 22.2% (95% CI: 2.5, 37.8; p = 0.029) and 24.7% (95% CI: 6.4, 39.5; p = 0.011), in the SP-AS3 and AQ3-AS3 groups, respectively. Narrowing the case definition for clinical malaria to those with >5,000 parasites (par)/ µL of blood yielded a PE against the first or only episode of 48.9% (95% CI: 12.2, 70.3; p = 0.015) in the SP-AS3 group. The PE against the first or only episode of mild anaemia (Hb <11.0 g/dL) was 20.3% (95% CI: 4.0, 33.9; p = 0.017) in the AQ3-AS3 group; details are shown in table 2. Kaplan-Meier survival plots of the observed data are shown in Figure S3. CD3 did not provide a significant PE for any of the outcomes measured. None of the IPTi regimens appeared to provide statistically significant protection against all-cause outpatient sick visits, hospitalizations with malaria, or all-cause hospitalizations (table 2). There was no evidence that the assumption of proportional hazards was violated when we assessed the interaction between age and treatment in the time-dependent Cox model, and comparison of the crude and adjusted models suggested adjustment for co-variates was not required as inclusion of co-variates did not affect the point estimate or its precision.

**Table 2:** Incidence of the main outcomes during the first year of life.

Outcomes	Placebo	SP-AS3	AQ3-AS3	CD3
**1^st^ or only cl mal**	**(n = 337)**	**(n = 339)**	**(n = 347)**	**(n = 342)**
Events/PYAR	158/161.0	130/176.3	137/181.4	136/166.2
Rate	0.98	0.74	0.76	0.82
PE % (95% CI)	Reference:	25.7 (6.3, 41.1)	25.9 (6.8, 41.0)	16.3 (-5.2, 33.5)
*p*-Value		0.029	0.011	0.324
**All episodes cl mal**	**(n = 337)**	**(n = 339)**	**(n = 347)**	**(n = 342)**
Events/PYAR	263/197.0	209/201.1	213/212.0	233/195.0
Rate	1.33	1.04	1.00	1.20
PE % (95% CI)	Reference:	22.2 (2.5, 37.8)	24.7 (6.4, 39.5)	10.5 (–11.6, 28.2)
*p*-Value		0.029	0.011	0.324
**Cl mal (>5,000par/µL)**	**(n = 346)**	**(n = 350)**	**(n = 354)**	**(n = 347)**
Events/PYAR	38/206.3	20/213.1	25/219.8	36/203.0
Rate	0.18	0.09	0.11	0.18
PE % (95% CI)	Reference:	48.9 (12.2, 70.3)	41.2 (2.5, 64.5)	3.4 (−52.3, 38.8)
*p*-Value		0.015	0.040	0.880
**Anaemia (Hb<11 g/dL)**	**(n = 321)**	**(n = 335)**	**(n = 324)**	**(n = 326)**
Events/PYAR	232/114.8	221/128.9	214/127.3	223/118.5
Rate	2.02	1.71	1.68	1.88
PE % (95% CI)	Reference:	16.4 (−0.6, 30.4)	20.3 (4.0, 33.9)	8.0 (−10.6, 23.5)
*p*-Value		0.057	0.017	0.375
**Anaemia (Hb<8 g/dL)**	**(n = 350)**	**(n = 355)**	**(n = 357)**	**(n = 351)**
Events/PYAR	59/199.3	45/209.5	51/213.4	52/198.7
Rate	0.30	0.21	0.24	0.26
PE % (95% CI)	Reference:	27.5 (−6.9, 50.8)	23.1 (−11.9, 47.2)	11.4 (−28.6, 39.0)
*p*-Value		0.105	0.170	0.525
**Out-patient visits**	**(n = 337)**	**(n = 339)**	**(n = 347)**	**(n = 342)**
Events/PYAR	1996/210.7	1947/211.1	2125/221.5	2051/206.6
Rate	9.47	9.22	9.59	9.93
PE % (95% CI)	Reference:	2.6 (−4.9, 9.7)	−1.3 (−9.4, 6.2)	−4.8 (−13.2, 2.9)
*p*-Value		0.482	0.746	0.229
**All hospitalizations**	**(n = 337)**	**(n = 339)**	**(n = 347)**	**(n = 342)**
Events/PYAR	137/210.9	127/211.4	147/221.7	130/206.9
Rate	0.65	0.60	0.66	0.63
PE % (95% CI)	Reference:	7.5 (−19.7, 28.5)	−2.1 (−32.1, 21.1)	3.3 (−28.6, 27.3)
*p*-Value		0.553	0.875	0.818
**Hospitalizations with**:				
**Clinical malaria**	**(n = 337)**	**(n = 339)**	**(n = 347)**	**(n = 342)**
Events/PYAR	57/210.9	61/211.4	67/221.7	58/206.9
Rate	0.27	0.29	0.30	0.28
PE % (95% CI)	Reference:	−6.8 (−61.5, 29.4)	−11.8 (−67.9, 25.5)	−3.7 (−60.6, 33.0)
*p*-Value		0.756	0.590	0.871

Cl mal  =  clinical malaria; PYAR  =  person-years-at-risk; PE  =  protective efficacy; CI  =  confidence interval. All episodes (GEE Poisson model); other outcomes (Cox model).

### Post-dose Efficacy against Malaria

The PE against the first or only episode of clinical malaria within a period of 30 days after the 1^st^ course of IPTi was 88.7% (95% CI: 50.9, 97.4; p = 0.004) in the SP-AS3 group, 66.6% (95% CI: 15.2, 86.8; p = 0.021) in the AQ3-AS3 group, and 66.1% (95% CI: 14.0, 86.6; p = 0.023) in the CD3 group. The corresponding figures after the 2^nd^ course of IPTi were 86.6% (95% CI: 61.8, 95.3; p<0.001), 73.1% (95% CI: 40.9, 87.7; p = 0.001), and 61.8% (95% CI: 23.3, 81.0; p = 0.007) in the SP-AS3, AQ3-AS3 and CD3 groups, respectively. By contrast, the corresponding PEs after the 3^rd^ course of IPTi were far lower and not statistically significant (table 3).

**Table 3:** Incidences of the primary outcome 30 days after each course of IPTi.

Outcomes	Placebo	SP-AS3	AQ3-AS3	CD3
**Cl mal after IPTi-1**	**(n = 337)**	**(n = 339)**	**(n = 347)**	**(n = 342)**
Events/PYAR	17/27.1	2/27.8	6/28.3	6/27.9
Rate	0.63	0.07	0.21	0.22
PE % (95% CI)	Reference:	88.7 (50.9, 97.4)	66.6 (15.2, 86.8)	66.1 (14.0, 86.6)
*p*-Value		0.004	0.021	0.023
**Cl mal after IPTi-2**	**(n = 320)**	**(n = 323)**	**(n = 322)**	**(n = 315)**
Events/PYAR	28/25.2	4/26.4	8/26.3	11/25.6
Rate	1.11	0.15	0.30	0.43
PE % (95% CI)	Reference:	86.6 (61.8, 95.3)	73.1 (40.9, 87.7)	61.8 (23.3, 81.0)
*p*-Value		<0.001	0.001	0.007
**Cl mal after IPTi-3**	**(n = 268)**	**(n = 273)**	**(n = 288)**	**(n = 246)**
Events/PYAR	23/20.9	15/21.9	17/23.0	18/19.8
Rate	1.10	0.68	0.74	0.91
PE % (95% CI)	Reference:	38.1 (−18.7, 67.7)	33.4 (−24.7, 64.4)	17.5 (−52.9, 55.5)
*p*-Value		0.149	0.205	0.542

Cl mal  =  clinical malaria; PYAR  =  person-years-at-risk; PE  =  protective efficacy; CI  =  confidence interval. All outcomes (Cox model).

The pooled post-dose analysis using biweekly time versus treatment interaction models showed that the duration of protective efficacy was longest (5 to 8 weeks) for the combinations based on sulphadoxine-pyrimethamine and amodiaquine reflecting their longer half-lives compared to CD3. It also showed that the protective effect declined rapidly thereafter and was no longer evident beyond 8 weeks (Figure S4).

### Per-protocol Analyses

The per-protocol analyses provided similar estimates, and none of the outcomes (PEs) differed by more than 15% as compared to ITT analyses. Therefore, only the ITT analyses are presented.

### Tolerability

Those taking study drugs were more likely to vomit than those taking placebo, prevalence ratios 1.77 (95% CI: 1.14, 2.74; p = 0.010), 2.15 (95% CI: 1.41, 3.28; p<0.001), and 2.36 (95% CI: 1.55, 3.59; p<0.001) in the SP-AS3, AQ3-AS3, and CD3 groups, respectively. Out of 11,568 drug doses administered, only 2.1% were vomited.

### Safety and Morbidity

There were 593 serious adverse events (SAEs) recorded during the 1^st^ year of life. Of these SAEs, 55 were deaths and 538 were hospitalizations. Although, the number of deaths was highest in the CD3 group, their difference was not statistically significant. The one death classified as possibly related in the CD3 arm was the case of a 3 month old female infant born on 27th August 2005 and enrolled into the study on 3rd October 2005. First dose of IPTi study drug was administered from 31st October to 2nd November 2005 and the second dose from 28th to 30th November 2005. She was well when both the first and second doses of IPTi were administered. She left the study area with the mother for their rural home on 1st December 2005 when she fell ill with vomiting of feeds and diarrhoea. The stool was watery, copious in amount and frequent. There was no history of fever, convulsions or cough. No treatment was sought on that day. The following day, she became restless and developed fast breathing, and again no treatment was sought. She passed away on 3rd December 2005, the third day of illness at home.

There was no significant difference in the number of SAEs in the SP-AS3, AQ3-AS3, and CD3 groups as compared to placebo. Fifteen were assessed as possibly related to study drug, 6 in the SP-AS3 group, 5 in the AQ3-AS3 group, and 4 in the CD3 group (table 4). The SAEs classified as possibly related were hospitalizations due to expected childhood illnesses which occurred within one month of administering IPTi, and further evaluation showed that none of the SAEs were directly related to any of the administered study drugs. No serious cutaneous adverse events were noted, and no cases of severe haemolysis were recorded. Male study participants in the CD3 arm (n = 251) with G6PD deficiency were more likely than those without G6PD deficiency to have moderate-to-severe anaemia when compared to their counterparts in the placebo arm (n = 263), PE -166.8% (95% CI: −497.5, −19.1; p<0.017).

**Table 4:** Summary of Serious Adverse Events (SAEs) during the first year of life.

Type of AE	Placebo	SP-AS3	AQ3-AS3	CD3
**SAEs**	**(n = 337)**	**(n = 339)**	**(n = 347)**	**(n = 342)**
**Deaths**				
Not related	9	9	14	15
Unlikely	3	1	1	2
Possibly related	–	–	–	1
Total	12	10	15	18
**Hospitalizations**	135	128	147	128
**All SAEs**				
Events/PYAR	147/1533.8	138/1530.3	162/1683.9	146/1549.8
Rates	0.10	0.09	0.10	0.09
RR (95% CI)	Reference:	0.94 (0.74, 1.27)	1.00 (0.79, 1.27)	0.98 (0.75, 1.27)
*p*-Value		0.617	0.975	0.896
Possibly related	5	6	5	4

RR  =  Relative risk; PYAR  =  person-years-at-risk; outcome (Poisson model).

### Rebound Analyses

The period under observation for the ‘rebound analyses’ started from 30 days after the 3^rd^ course of IPTi (10 months of age) until 24 months of age, and all participants who received at least one dose of study drug and were still being followed-up during that period were included in the analyses (table 5). There was no statistically significant rebound for the first or only episode of clinical malaria, multiple episodes of clinical malaria, mild anaemia, and moderate-to-severe anaemia. For a more specific definition of clinical malaria (>5,000 par/µL of blood), statistically significant rebound was noted only in the CD3 group, PE −63.0% (95% CI: −142.5, −10.5; p = 0.014) (table 5).

**Table 5:** Incidences of main outcomes between 10 to 24 months of age follow-up.

Outcomes	Placebo	SP-AS3	AQ3-AS3	CD3
**1^st^ or only cl mal**	**(n = 282)**	**(n = 285)**	**(n = 296)**	**(n = 262)**
Events/PYAR	151/200.2	157/211.4	162/210.1	147/179.1
Rate	0.75	0.74	0.77	0.82
PE % (95% CI)	Reference:	1.5 (−23.1, 21.2)	−1.1 (−26.3, 19.0)	−7.3 (−34.6, 14.5)
*p*-Value		0.892	0.92	0.544
**All episodes cl mal**	**(n = 283)**	**(n = 286)**	**(n = 297)**	**(n = 262)**
Events/PYAR	456/284.0	464/292.9	475/297.4	480/256.7
Rate	1.61	1.58	1.60	1.87
PE % (95% CI)	Reference:	1.3 (−19.8, 18.7)	1.0 (−20.8, 18.1)	−16.5 (−41.2, 3.9)
*p*-Value		0.892	0.957	0.120
**Cl mal (>5,000 par/µL)**	**(n = 283)**	**(n = 286)**	**(n = 297)**	**(n = 262)**
Events/PYAR	42/288.7	47/298.0	51/300.3	61/255.2
Rate	0.15	0.16	0.17	0.24
PE % (95% CI)	Reference:	−8.6 (−64.6, 28.4)	−16.7 (−75.6, 22.4)	−63.7 (−142.5, −10.5)
*p*-Value		0.699	0.458	0.014
**Anaemia (Hb<11 g/dL)**	**(n = 279)**	**(n = 281)**	**(n = 295)**	**(n = 260)**
Events/PYAR	239/96.7	238/105.5	254/107.3	211/93.7
Rate	2.47	2.26	2.37	2.25
PE % (95% CI)	Reference:	8.2 (−9.8, 23.3)	3.3 (−15.4, 19.0)	6.5 (−12.5, 22.3)
*p*-Value		0.349	0.707	0.478
**Anaemia (Hb<8 g/dL)**	**(n = 282)**	**(n = 285)**	**(n = 298)**	**(n = 261)**
Events/PYAR	66/257.5	53/268.9	62/274.2	57/243.6
Rate	0.26	0.20	0.23	0.23
PE % (95% CI)	Reference:	22.2 (−11.7, 45.8)	11.2 (−25.6, 37.2)	8.0 (−31.1, 35.5)
*p*-Value		0.173	0.502	0.643

Cl mal  =  clinical malaria; PYAR  =  person-years-at-risk; PE  =  protective efficacy; CI  =  confidence interval. All episodes (GEE Poisson model); other outcomes (Cox model).

## Discussion

Our results indicate that two long-acting combinations (SP-AS3 and AQ3-AS3), but not a short-acting combination (CD3) provided significant protection against the first or only episode of clinical malaria and anaemia in this area with year round malaria transmission with high seasonal intensity, near universal usage of ITNs, and a high level of *P. falciparum* resistance to SP. The SP-AS3 and AQ3-AS3 groups also provided much higher protection against the first or only episode of high density clinical malaria. These results suggest that the prophylactic effect is more important for the mode of action of IPTi. It is reassuring to note that protective efficacy against multiple episodes of clinical malaria was fairly similar to the primary endpoint, as the multiple episodes endpoint has more direct relevance to public health interpretation.

The PE of 26% against clinical malaria provided by SP-AS3 is similar to that reported in earlier IPTi trials with SP alone [9]–[12] with the exception of the initial Tanzanian study which reduced clinical malaria by 59% [7]. Our results also suggest that AQ may be a suitable alternative to SP for IPTi. However, our results with AQ3-AS3 (PE 23% against clinical malaria) were less dramatic than the 65% protection reported using AQ alone in Tanzania. [8]. The Tanzanian trial of AQ was quite different in that IPTi administration was not linked to EPI and the 3 doses were given 60 days apart, beginning at 12–16 weeks of age. We found that AQ3-AS3 was tolerated as well as the other IPTi drug combinations used in this trial. Although the acceptance of AQ is sometimes poor among adults and pregnant women being treated for clinical malaria [51]–[54], data from our blinded trial does not suggest that tolerability is a problem in infants.

In contrast to the earlier study in Tanzania [19], our trial did not show a sustained benefit of IPTi in the second year of life. It is not clear why that initial trial showed a much higher protection in the first year of life and observed persistence of protection in the second year of life, whereas subsequent trials have not [55]. A comparative analysis of the Tanzanian trial and another IPTi trial in Mozambique suggested that the high ITN coverage in Tanzania at the time of the trial was likely responsible for the dramatically higher efficacy there [56]. The results of our trial, in which ITN ownership and usage was very high, do not lend support to that hypothesis. The observed difference in the second year could be explained by a decrease in malaria transmission in the Tanzanian study during the study period, as recently suggested by Gosling et al. [55]. It may also be that the results were at the higher end of the normal variation of protective efficacy, with subsequent trials showing regression toward the mean. While there is evidence that the burden of malaria in early infancy is generally lower than in later infancy in many settings, the results of this and other trials suggest that there is a significant protective efficacy after the first dose of IPTi. Therefore, while the EPI schedule may not provide the optimal spacing (especially the gap between 14 weeks and 9 months), it does provide a readily usable delivery platform. Furthermore, earlier randomised controlled trials with ITNs in the same study site showed that most of the benefit of ITNs occurred in the first 6 months of life and that this became apparent from 2 months of age onwards [57].

Similar to the previous trials with SP alone, we also observed a high PE with SP-AS3 in the 30 days after the first two courses of IPTi suggesting that the impact of rising resistance of *P. falciparum* to SP as evidenced by decreased efficacy in the treatment of symptomatic children with malaria may not be as dramatic when SP is used for IPTi in early infancy. It may be that the presence of maternal antibodies and foetal haemoglobin during the first two courses of IPTi result in lower parasite densities in asymptomatic children and therefore in better clearance, even with imperfect or even failing anti-malarial drugs. Given the short terminal half-life of AS, and our data suggesting that the prophylactic rather than the treatment effect of IPTi is central to its protective efficacy, it is unlikely that the addition of AS to SP had a substantial impact on its PE as IPTi; without an SP-alone arm, it is not possible to confirm this hypothesis. The reason behind our observed reduction in PEs, which occurs after the 5-month interval between the second and third courses of IPTi, is not clear. It may be the result of a complex interplay of factors including the loss of maternal antibodies, the development of naturally acquired immunity by all children (including the healthy survivors in the placebo group, thereby diminishing any differences between those in the intervention and placebo arms), limited exposure to malaria parasites (due to protection by high usage of ITNs), and even though a certain amount of relative under dosing may have occurred due to natural weight-gain with age, any effects of relative under dosing would likely have been randomly distributed across the study arms.

A closer look at the results of the post-dose analysis using biweekly time interaction models shows that the post-treatment prophylactic effect does not extend beyond 5 to 8 weeks after receiving SP-AS3 or AQ3-AS3. The results are very similar to a more detailed analysis of the duration of protection against clinical malaria provided by Cairns et al on the data from Navrongo. These also found that the duration of protective efficacy was short-lived and lasted for 4 to 6 weeks only, reflecting the prophylactic effect of IPTi (the duration varied with the endpoint used in the analysis). Similar to our study, there was no evidence for a sustained effect thereafter [20].

There was no evidence of a rebound effect with the SP- and AQ- containing combinations in the second year of life, suggesting that IPTi does not have a negative impact on the development of immunity to malaria. However, compared to the placebo arm, a higher rate of clinical malaria (>5,000 par/µL of blood) was noted in the CD3 group. It is possible that this may have been a chance finding, given the absence of a similar rebound effect with any of the other clinical endpoints, and considering the unlikely influence of CD3 on the acquisition of natural immunity due to its relatively short terminal half-life.

Some limitations to our study should be noted. First, SP and AQ were used in combination with AS, but this study was not designed to address the contribution of the rapidly eliminated artemisinin derivatives, and their contribution to the effect of IPTi still remains unclear. Any direct benefit would have resulted in the improved radical cure of existing infections over and above that of SP or AQ mono-therapy. The benefit would be greatest to infants with higher density parasitaemias, as low density infections would more likely have been cleared successfully even in the presence of mild to moderate resistant infections [58]. Thus one of the main benefits of adding an artemisinin derivative is probably the prevention of abuse of mono-therapies by reducing chances of the further development of resistance in the population [18]. However, this needs to be weighed against the risk of decreased adherence if combination therapy for IPTi results in longer and more complex regimens (e.g. single dose SP versus 3 days of SP-AS3). Thus our results obtained with these multi-day regimens in this trial should be interpreted with some caution as all doses were administered under direct supervision, which clearly would not be feasible for programmatic use.

Second, the high usage of ITNs may potentially provide competing or added benefits in reducing clinical malaria and anaemia. The effect of ITNs as a potential effect modifier could not be assessed in our study because ITNs were provided to all participants at enrolment. This was done because the benefits of using ITNs had already been conclusively demonstrated in the same study area [25], and there was already a very high rate of ownership and usage in the community. Third, we facilitated hospitalization–generally at the Provincial Hospital–for all participants. Our conservative approach to hospitalization may have resulted in a dilution of any effect of IPTi on hospitalization rates. Fourth, preliminary sample size calculations were based on assumptions of 90% power and 40% efficacy similar to the initial Tanzanian trial [7]. Though, the PE for our trial was much lower (similar to more recent IPTi trials), we were still able to achieve statistical significance because our assumption of a median time to first episode of clinical malaria was overly conservative, along with our relatively high estimates of loss to follow-up and mortality. Fifth, our trial was not designed to investigate mortality, though the pooled analysis suggests that IPTi does not reduce mortality when compared with placebo [59].

### Conclusion

In conclusion, our results support the view that long-acting but not short-acting regimens are suitable for IPTi in areas of year round malaria transmission with high seasonal intensity and high ITN coverage, and that the prophylactic rather than the treatment effect of IPTi appears central to its protective efficacy. The results of this study are relevant for other IPT strategies such as IPT in pregnancy (IPTp) and IPT in children (IPTc), and contribute to the development of target product profiles for alternative drugs to be used for IPT. There is need to confirm these results in other settings, to investigate the potential gap between efficacy and effectiveness for multi-day regimens, and to test other long-acting combinations such as dihydroartemisinin-piperaquine.

## Supporting Information

Checklist S1 CONSORT Checklist
(0.23 MB PDF)

Analysis Plan S1 Analysis Plan
(0.93 MB DOC)

Protocol S1 Trial Protocol
(0.27 MB DOC)

Figure S1 Trial time-line for participants of the IPTi trial in western Kenya Note: PENT = diphtheria-tetanus toxoid-pertussis-hepatitis B-Haemophilus influenza type b vaccine; OPV = oral polio vaccine; EPI = Expanded Programme of Immunization; IPTi = Intermittent Preventive Treatment of infants.
(0.68 MB TIF)

Figure S2 Trial profile Flowchart
(0.43 MB TIF)

Figure S3 Kaplan-Meier plots showing the cumulative proportion of children remaining free of clinical malaria episodes between the first dose of IPTi and 12 months of age.
(0.31 MB TIF)

Figure S4 Biweekly PEs pooled post-IPTi 2 and 3 for SP-AS3, AQ3-AS3, and CD3. Note: the error bars indicate 95% confidence intervals.
(0.06 MB TIF)

Consent S1 Parental/Guardian consent form.
(0.05 MB DOC)
