# Supplementary material for: Intermittent Preventive Treatment in Infants for the Prevention of Malaria in Rural Western Kenya: A Randomized, Double-Blind Placebo-Controlled Trial
Source: PLoS One. 2010 Apr 2;5(4):e10016. doi: 10.1371/journal.pone.0010016 (PMC2848869; doi:10.1371/journal.pone.0010016)
Supplement: Analysis Plan S1 — Analysis Plan (0.93 MB DOC) [file pone.0010016.s002.doc]

**Efficacy and safety of pediatric immunization-linked preventive intermittent treatment with anti-malarials in decreasing anemia and malaria morbidity in rural western Kenya**

Assessment Plan

##### Draft – 26^th^ June 2007

## Introduction

This document describes the methods used for the processing and analysis of data collected during the trial titled: “**Efficacy and safety of pediatric immunization-linked preventive intermittent treatment with anti-malarials in decreasing anemia and malaria morbidity in rural western Kenya**”.

This is a clinical trial designed to measure the efficacy in prevention of malaria morbidity, of giving one of 3 anti-malarial regimens: a single dose of sulphadoxine-pyrimethamine plus three doses of artesunate (SP/AS3), 3 doses of chlorproguanil-dapsone (Lapdap), or 3 doses of amodiaquine plus three doses of artesunate (AQ/AS3) intermittently at 10 weeks, 14 weeks, and 9 months of age to infants at the time they receive routine vaccinations as part of the Expanded Programme on Immunizations. The strategy is hereafter referred to as “Intermittent Preventive Treatment in Infants” (IPTi).

## Primary objective

Compare the efficacy of iron supplementation and one of three anti-malarial regimens (SP/AS3, Lapdap, or AQ/AS3) delivered at the time of routine EPI visits at 10 weeks, 14 weeks, and 9 months of age with iron supplementation alone (plus placebo) in the prevention of the first or only episode of clinical malaria during the first 12 months of life.

## Secondary objectives

1. Compare the efficacy of iron supplementation plus IPTi with one of three antimalarial regimens (SP/AS3, Lapdap, or AQ/AS3) given at routine EPI visits with iron supplementation alone (plus placebo) on the prevention of moderate-to-severe anemia (hemoglobin [Hb] <8) in the first year of life.
2. Compare the efficacy of iron supplementation and IPTi with one of three aforementioned regimens given at routine EPI visits with iron supplementation alone (plus placebo) on the prevention of all-cause hospitalization in the first year of life.
3. Assess the impact of IPTi with one of three aforementioned regimens on serologic responses to EPI vaccines (Polio, Diphtheria, Tetanus, Pertussis, Hepatitis B, *Haemophilus Influenza* type B, and Measles).*
4. Assess the impact of IPTi with one of three aforementioned regimens (particularly SP/AS3) on the nasal carriage rates of *Haemophilus influenza* type b.*

*Points 3 and 4 will be assessed separately from this assessment plan.

## Study design

This is a randomized, double-blind, placebo-controlled study (RCT).

## Treatment

The first dose of each course of study drug was administered and supervised at the healthcare centre by the study nurse. The second and third doses of each course of study drug were administered and supervised at home by a study compliance monitor. All the doses were observed for a 30 minute period, and if vomited before the end of that period then a repeat dose was administered and supervised at the healthcare centre by the nurse. The tablets were crushed and mixed with pharmaceutical-grade cherry syrup immediately before being administered.

## Sample Size

The sample size required for 90% power to detect a 40% protective efficacy in the prevention of clinical malaria in the first year of life assuming α=0.017, is 250 per arm. Alpha was adjusted to 0.017 in order to account for multiple comparisons (3 arms each compared to the placebo group, Bonferroni adjustment 0.05/3 tests=0.017). Therefore, the study required a sample size of 1000 infants (250 in each of 4 groups) who would complete the primary follow-up period (to 12 months of age). We expected that 11% of infants might have the outcome measure at the time of receiving first study drug, and thus would be excluded from the primary analysis. Including this decrease and adjusting for a 10% loss to follow up and 13% mortality, the study required a total of 1516 infants (379 in each arm).

## Sampling procedure

All infants attending one of the study clinics for the 6-week vaccination visit (the first post-birth scheduled EPI visit in Kenya) were screened for enrolment. The parents or guardians of infants meeting the study inclusion criteria and not meeting any of the exclusion criteria were offered enrolment in the study.

## Assignment to interventions

All infants whose parents or guardians agreed to participate in the study were assigned to one of the 4 treatment groups through permuted block randomization at the first intervention visit. The randomization code was prepared before the trial started, and unique study ID numbers were issued to participants at recruitment but the assignment to groups occurred at the second visit when IPTi dose 1 was given. These randomization procedures were expected to minimize the possible influence of geographical location and seasonal malaria variations.

## Inclusion / exclusion criteria

*Inclusion criteria: Infants attending any one of the 4 study clinics (Abidha, Ongielo, Lwak, and Saradidi) for immunization at the 6-week visit were offered enrolment in the study.*

1. Age 5 weeks to 16 weeks

2. Parent or guardian currently resident in Asembo (or Gem).

3. Parent or guardian has given permission for their child to participate (written informed consent)

*Exclusion criteria*

1. Known allergy to any of the study drugs

2. Current Cotrimoxazole prophylaxis

2. Concomitant disease requiring hospitalization or transfusion

3. Plans to be away from the study area for more than 6 months during the next year

## Loss / withdrawal criterion

All recruited infants who present with serious adverse events (SAEs) attributable to the administration of IPTi for malaria were withdrawn from receiving any more courses of study drug but their follow–up visits were continued.

Recruited infants whose parents or legal guardians refused to continue with the study at any point are also considered lost or withdrawals.

Lastly, all recruited infants who migrate (more than three months outside the study area) are suspended from the study but allowed to re-enter upon returning, or considered lost or withdrawals if they did not return during the study period.

## Values included in the analysis

*Intention to treat*

The main analysis will be performed according to a modified Intention-to-treat (ITT) approach because of the 1 month period between enrolment and assignment to treatment arms. Therefore, the analysis will include all randomized infants who received at least one dose of IPTi. Among these infants, all data available up to the time of death, loss, withdrawal or study completion (up to 12 months of age), are included regardless of whether they received all or part of the interventions. Children who died while actively enrolled are considered censored at the date of death. Children whose parents withdrew the child from participation are considered censored on the date the withdrawal occurred. Children who migrated out of the study area are considered censored at the date of the last scheduled study visit or unscheduled outpatient sick visit or iron compliance home visit. For the analysis of multiple episodes of malaria, children who had previously been censored because of loss-to-follow-up were re-admitted to the risk set on the day that they attended a scheduled study visit or made an unscheduled outpatient sick visit.

*According to protocol*

An alternative analysis will be done according to the protocol, hereafter referred to as “per-protocol”. This analysis will include only data for infants who received all the three courses of study drug within 28 days of the appointed treatment date, according to the protocol, up to the time of death, loss, withdrawal or study completion (up to 12 months of age).

## Summary of endpoints

Incidence of first or only episode of clinical malaria in the first year of life (ITT and per-protocol)

Incidence of first or only episode of anemia (mild, moderate-to-severe, and critical) in the first year of life (ITT and per-protocol)

Incidence of clinical malaria in the first year of life (all episodes, ITT and per-protocol)

Incidence of first or only episode of clinical malaria with high parasite density (>5000 parasites per microlitre)

Prevalence of parasitaemia at 12 months (ITT and per-protocol)

Prevalence of anemia (mild, moderate-to-severe, and critical) at 12 months (ITT and per-protocol)

Mean Hb at 12 months (ITT and per-protocol)

Prevalence of weight for age Z-score <-2 at 12 months

Prevalence of height for age Z-score <-2 at 12 months

Incidence of clinical malaria within a one month period after each dose of IPTi (ITT and per-protocol)

Incidence of anemia (mild, moderate-to-severe, and critical) within a one month period after each dose of IPTi (ITT and per-protocol)

## Analysis variables

The origin of the variables used in the analysis may be those directly described in the questionnaires or variables derived from those directly observed. Two types of variables are considered in the study:

- Continuous variables: obtained by assessing or measuring quantitative values among study subjects
- Categorical variables: obtained by assessing qualitative values among study subjects.

## Data management and breaking code

Data was collected on specially designed forms (Teleform software) and entered by scanning into an Access database. All data was stored and processed in a dedicated secure directory on a central server. A data manager performed daily cross-checking routines to detect and correct any data entry discrepancies. Discordances detected at this point were recorded in a log file (audit trail) permitting quality control of the data checking process. Weekly checks for duplicate records, completeness of the databases, range, consistency and referential integrity were also performed. The cleaned and locked database files will be handed to the DSMB in exchange for the randomization code. Any subsequent changes to the dataset for analysis will be carefully documented. As extended follow-up will continue after the first analysis, the field team will remain blind to avoid compromising the analysis at the end of the extended follow-up.

## General statistical methods

Quantitative variables will be described using the following statistics: total number of observations, mean value, standard deviation, minimum and maximum of each value. Comparison of these quantitative values will be measured by the Wilcoxon test for only two levels; otherwise the Kruskal Wallis test will be used. If adjustments for confounding factors are necessary, linear regression models will be carried out in the scale where the variable has a Normal distribution. Qualitative variables will be described according to frequency and percentage of frequency with respect to the whole sample. The chi-square test will be used to compare the values according to whether they belong to the treatment or placebo groups. Comparative tables with expected values below 5 in any of the response cells will be assessed using Fisher’s test. If adjustments for confounding factors are necessary, logistic regression will be used if the categorical variables have only two levels, otherwise log-linear models will be used. The goodness of fit will be evaluated for the models. Statistical analysis will be performed using SAS version 9.1 and STATA version 8.

## Data analysis

The data analysis will be divided into sections:

1. Preliminary analysis

- Trial profile
- Description of baseline characteristics
- Iron compliance
- Insecticide-treated bednet (ITN) use

1. Effects of IPTi

- 1^st^ or only episode of clinical malaria up to 12 months of age (ITT and per-protocol)
- 1^st^ or only episode of anemia (mild, moderate-to-severe, and critical) up to 12 months of age (ITT and per-protocol)
- All episodes of clinical malaria up to 12 months of age (ITT and per-protocol)
- 1^st^ or only episode of clinical malaria (alternative parasitaemia thresholds) up to 12 months of age (ITT and per-protocol).
- All episodes of clinical malaria (alternative parasitaemia thresholds) up to 12 months of age (ITT and per-protocol)
- Prevalence of clinical malaria at 12 months of age (ITT and per-protocol)
- Prevalence and density of parasitaemia at 12 months of age (ITT and per-protocol)
- Prevalence and severity of anemia at 12 months of age (ITT and per-protocol)
- Mean Hb levels at 12 months of age (ITT and per-protocol)
- Prevalence of weight for age Z-score <-2 at 12 months of age (ITT and per-protocol)
- Prevalence of height for age Z-score <-2 at 12 months of age (ITT and per-protocol)
- 1^st^ or only episode of clinical malaria within a one month period after each course of IPTi (post-dose) (ITT and per-protocol)
- 1^st^ or only episode of anemia (mild, moderate-to-severe, and critical) within a one month period after each course of IPTi (post-dose) (ITT and per-protocol)

1. Safety of IPTi

- Vomiting
- Deaths
- Dermatological adverse events
- All-cause outpatient visits
- Malaria and anemia related outpatient visits
- All-cause hospitalizations
- Malaria and anemia related hospitalizations

1. Other sub-analyses

- Repeat analysis “B” for children who met the primary outcome at IPTi visits
- Compliance of delivering IPTi concurrently with EPI schedule

## Preliminary analysis

# Trial profile

The profile will document the number of children enrolled and randomized to each of the 4 study arms, and completing follow-up. The numbers of withdrawals and deaths between doses 1 and 2, doses 2 and 3 and between dose 3 and age 12 months will also be included. The proportions of children receiving doses 1, 2 and 3 of each drug regimen and placebo will be compared separately using the Chi-squared test.

# Trial profile for intention-to-treat analysis

Table X. Trial Profile by intention-to-treat

Appendix 1

# Trial profile for per protocol analysis

Table X. Trial Profile per-protocol

Appendix 2

# Statistical analysis, tables, figures and individual data

# Description of baseline characteristics

Baseline characteristics of each drug regimen and placebo recipients will be compared. The following variables will be included:

Sex

Age at the 1^st^ treatment visit

Z-score of weight for age at the 1^st^ treatment visit (using EPINUT tables’ version 6)

Z-score of height for age at the 1^st^ treatment visit

Z-score of weight for height at the 1^st^ treatment visit

Hemoglobin genotypes assessed at the 12 month visit

G6PD status

ITN use at the 1^st^ treatment visit

More emphasis will be given to the size of any differences than to statistical significance, as well as to the relationship with the outcome, since this is what affects the degree of confounding. Individual variables listed above will be adjusted for if there are imbalances between the groups and they independently alter the efficacy estimate by 15% or more.

# Iron compliance

A non-supervised supplement of once daily oral iron (roughly 2 mg/kg/day) was given to all study children for 4 months (from recruitment at 10 weeks until 6 ½ months of age). A one month supply (2 bottles containing 25ml each) was given at the 10-week visit, an additional one month supply was given at the 14-week visit, and a 2 month supply (4 bottles) was given at the 18 week visit. Compliance with iron therapy was assessed by using a measuring stand to determine quantity used at the 14 week, 18 week, and 6 ½ month study follow-up visits. Children who received more than 67% of the total anticipated dosage of iron syrup will be considered to have had good compliance and the proportion of such children compared between each drug regimen and placebo using the Chi-squared test.

Table X. Distribution of age at each course of treatment according to treatment group

| Variable | Treatment | | | | | | | | | |
| --- | --- | --- | --- | --- | --- | --- | --- | --- | --- | --- |
|  | Placebo  (XXX) | | SP/AS3  (XXX) | | LAPDAP  (XXX) | | AQ/AS3  (XXX) | | TOTAL  (XXX) | |
|  | Mean | SD | Mean | SD | Mean | SD | Mean | SD | Mean | SD |
| Age (months) at recruitment | X.X | X.X | X.X | X.X | X.X | X.X | X.X | X.X | X.X | X.X |
| Age (months) at Dose 1 | X.X | X.X | X.X | X.X | X.X | X.X | X.X | X.X | X.X | X.X |
| Age (months) at Dose 2 | X.X | X.X | X.X | X.X | X.X | X.X | X.X | X.X | X.X | X.X |
| Age (months) at Dose 3 | X.X | X.X | X.X | X.X | X.X | X.X | X.X | X.X | X.X | X.X |

Table X. Distribution of baseline characteristics according to treatment group

| Variable | Treatment | | | | | | | | | |
| --- | --- | --- | --- | --- | --- | --- | --- | --- | --- | --- |
|  | Placebo  (XXX) | | SP/AS3  (XXX) | | LAPDAP  (XXX) | | AQ/AS3  (XXX) | | TOTAL  (XXX) | |
|  | Mean | SD | Mean | SD | Mean | SD | Mean | SD | Mean | SD |
| Weight | X.X | X.X | X.X | X.X | X.X | X.X | X.X | X.X | X.X | X.X |
| Height | X.X | X.X | X.X | X.X | X.X | X.X | X.X | X.X | X.X | X.X |
| Z-score: Height-for-age | X.X | X.X | X.X | X.X | X.X | X.X | X.X | X.X | X.X | X.X |
| Z-score: Weight-for-age | X.X | X.X | X.X | X.X | X.X | X.X | X.X | X.X | X.X | X.X |
| Z-score: Weight-for-height | X.X | X.X | X.X | X.X | X.X | X.X | X.X | X.X | X.X | X.X |
| G6PD genotypes |  |  |  |  |  |  |  |  |  |  |
| Hb genotypes |  |  |  |  |  |  |  |  |  |  |
| ITN use the previous night |  |  |  |  |  |  |  |  |  |  |
| “Good” Iron compliance |  |  |  |  |  |  |  |  |  |  |

Table X. Distribution of sex according to treatment group

| Variable | | Placebo  (XXX) | | SP/AS3  (XXX) | | LAPDAP  (XXX) | | AQ/AS3  (XXX) | |
| --- | --- | --- | --- | --- | --- | --- | --- | --- | --- |
|  |  | n | % | n | % | n | % | n | % |
| Sex | Male | XXX | XX% | XXX | XX% | XXX | XX% | XXX | XX% |
|  | Female | XXX | XX% | XXX | XX% | XXX | XX% | XXX | XX% |

¹: Chi-square test

Table X. Distribution of the study area according to treatment group

| Variable | | Placebo  (XXX) | | SP/AS3  (XXX) | | LAPDAP  (XXX) | | AQ/AS3  (XXX) | |
| --- | --- | --- | --- | --- | --- | --- | --- | --- | --- |
|  |  | n | % | n | % | n | % | n | % |
| IPTi Clinic | Lwak (1) | XXX | XX% | XXX | XX% | XXX | XX% | XXX | XX% |
|  | Abidha (2) | XXX | XX% | XXX | XX% | XXX | XX% | XXX | XX% |
|  | Ongielo (3) | XXX | XX% | XXX | XX% | XXX | XX% | XXX | XX% |
|  | Saradidi (4) | XXX | XX% | XXX | XX% | XXX | XX% | XXX | XX% |

## Effects of IPTi

### Data analysis

The objective will be to estimate the effect of IPTi on the incidence of the first or only episode of malaria and anemia observed in each infant in the four study arms during the first year of life. The statistical analysis will be based on the inclusion of all infants who received at least one dose of either SP/AS3 or Lapdap or AQ/AS3 or placebo, and will be analyzed by intention-to-treat. The time at risk begins on the day when the participant receives the first dose of the first course of IPTi study drug, and ends when the first case of malaria or anemia respectively is found or when there is a loss/withdrawal, refusal, migration or death of the infant studied. The first year of life is defined as all time up until the last clinical visit before 395 days of age, or visit 6 (designed to occur at 12 months of age) – which ever comes first.

The number of first episodes, as well as the person-time at risk statistics will be presented in the results. The primary result will be the unadjusted protective effect (PE), defined as 1-RR where RR is the hazard ratio given by the Cox regression model.

For the analysis of all malaria episodes in the first year of life, participants will be considered not at risk (and therefore not included in the risk set) for 14 days following each episode of malaria. As quinine was the antimalarial drug used for treating clinical malaria in those <1 year of age in this trial, and the half life of quinine is very short, it was felt that 14, rather than 28 days represented the most appropriate exclusion time.

Cox regression assumes that the hazard (risk) ratio between treatment groups is constant over time. This assumption regarding the proportionality of the hazard (risk) ratio will be analyzed using an approximation chart and assessing the interaction between age and effect of treatment with a time-dependent Cox regression model. If the proportionality assumption is not sustainable, the estimate of the hazards ratio will be presented at different time points of follow-up.

### Values of first or only episode of malaria

# Definitions

### Clinical malaria

#### Primary definition of clinical malaria

An episode of clinical malaria is defined as any child who comes to the healthcare centre with an axillary temperature of 37.5ºC or higher, OR whose caretaker gives a history of fever in the last 48 hours AND *P. falciparum* in its asexual form is present on a blood film examined at the CDC/KEMRI laboratory in Kisian. This definition excludes multiple episodes detected during follow-up of a single illness episode.

#### Alternate definition 1 of clinical malaria

Same as primary definition, but with parasitaemia threshold of over 500 parasites per microlitre of blood

#### Alternate definition 2 of clinical malaria

Same as primary definition, but with parasitaemia threshold over 2500 parasites per microlitre of blood

#### Alternate definition 3 of clinical malaria

Same as primary definition, but with parasitaemia threshold over 5000 parasites per microlitre of blood

#### Alternate definition 4 of clinical malaria

Same as primary definition, but with parasitaemia threshold over 15000 parasites per microlitre of blood

#### Alternate definition 5 of clinical malaria

Same as primary definition, but with parasitaemia threshold over 100000 parasites per microlitre of blood

#### Multiple malaria episodes

Same as primary definition, but considering all malaria episodes observed between dose 1 and age 12 months

**Post-dose clinical malaria**

The incidence of clinical malaria within a one month (30 day) period after each course of study drug.

Table X. Distribution of first or only episode of malaria according to treatment groups

| Events | Treatment | | | |
| --- | --- | --- | --- | --- |
|  | Placebo | | SP/AS3 | |
| Yes | XXX | XX.X% | XXX | XX.X% |
| No | XXX | XX.X% | XXX | XX.X% |
| Total | XXX | 100.0% | XXX | 100.0% |

| Events | Treatment | | | |
| --- | --- | --- | --- | --- |
|  | Placebo | | LAPDAP | |
| Yes | XXX | XX.X% | XXX | XX.X% |
| No | XXX | XX.X% | XXX | XX.X% |
| Total | XXX | 100.0% | XXX | 100.0% |

| Events | Treatment | | | |
| --- | --- | --- | --- | --- |
|  | Placebo | | AQ/AS3 | |
| Yes | XXX | XX.X% | XXX | XX.X% |
| No | XXX | XX.X% | XXX | XX.X% |
| Total | XXX | 100.0% | XXX | 100.0% |

Table X. Person-years at risk (PYAR) ratio

|  | Placebo  (n= ) | SP/AS3  (n= ) | LAPDAP  (n= ) | AQ/AS3  (n= ) |
| --- | --- | --- | --- | --- |
| Events | XX | XX | XX | XX |
| PYAR | XX.X | XX.X | XX.X | XX.X |
| Rate | X.XX | X.XX | X.XX | X.XX |
| PE (95% CI) | Reference group | XX.X  (.X-.X%) | XX.X  (.X-.X%) | XX.X  (.X-.X%) |
| p-value | - | X.XXX | X.XXX | X.XXX |

Event: first or only malaria episode, for parasites > 0, PYAR=person years at risk, PE=protective efficacy

Scope: from dose 1 until age 12 months

Note: p-value by Log Rank-test

Table X. Evaluation of potential confounding variables (15% level)

| Potential confounding variable | Adjusted treatment efficacy | p-value | Relative difference, % |
| --- | --- | --- | --- |
| None (unadjusted estimate) | XX.X% | X.XXX |  |
| Sex | XX.X% | X.XXX | XX.X |
| Age (months) at dose 1 | XX.X% | X.XXX | XX.X |
| Z-score: Height-for-age | XX.X% | X.XXX | XX.X |
| Z-score: Weight-for-age | XX.X% | X.XXX | XX.X |
| Z-score: Weight-for-height | XX.X% | X.XXX | XX.X |
| IPTi clinic | XX.X% | X.XXX | XX.X |
| G6PD status |  |  |  |
| Hb genotype |  |  |  |
| Vomiting |  |  |  |
| Presumptive quinine treatment |  |  |  |
| ITN use |  |  |  |
| No / Yes, confounding variables exist | | | |

Event: first or only malaria episode, for parasites > 0

Scope: from dose 1 until age 12 months

Table X. Risk Factors. Univariate Analysis

| Variable | | Hazard Ratio | (95%CI) | p-value |
| --- | --- | --- | --- | --- |
| Sex | Male | 1 |  | X.XXX |
|  | Female | X.XX | (X.XX; X.XX) |  |
| IPTi clinic | 01 | 1 |  | X.XXX |
|  | 02 | X.XX | (X.XX; X.XX) |  |
|  | ... | X.XX | (X.XX; X.XX) |  |
| Age (months) at dose 1 | for unit increase | X.XX | (X.XX; X.XX) | X.XXX |
| Z-score: Height-for-age | for unit increase | X.XX | (X.XX; X.XX) | X.XXX |
| Z-score: Weight-for-age | for unit increase | X.XX | (X.XX; X.XX) | X.XXX |
| Z-score: Weight-for-height | for unit increase | X.XX | (X.XX; X.XX) | X.XXX |
| … |  |  |  |  |

P-values by Wald test

### Values of multiple malaria episodes

All episodes of clinical malaria after dose 1 will be included in a Generalized Estimating Equations (GEE) Poisson regression model to take into account intra-individual variations and to make any adjustment for imbalances in baseline variables. An infant will not be considered at risk of a separate episode of clinical malaria, and therefore not contribute to the numerator or denominator of the risk set, for a period of 14 days after each episode. No assessment of the effect of the intervention on multiple episodes of anemia will be made due to the difficulty of estimating the duration of an episode of anemia.

A sensitivity analysis will be performed using case definitions of different specificity, as in the

Analysis of the primary endpoint.

Table X. Distribution of multiple malaria episodes

|  | Treatment | | | | | | | |
| --- | --- | --- | --- | --- | --- | --- | --- | --- |
|  | Placebo  (Mean) (%) | | SP/AS3  (Mean) (%) | | LAPDAP  (Mean) (%) | | AQ/AS3  (Mean) (%) | |
| Episodes per child | XXX | XX.X% | XXX | XX.X% | XXX | XX.X% | XXX | XX.X% |
| Total | XXX | 100.0% | XXX | 100.0% | XXX | 100.0% | XXX | 100.0% |

Scope: from dose 1 until age 12 months

Table X. Person-years at risk (PYAR) ratio

|  | Placebo  (n= ) | SP/AS3  (n= ) | LAPDAP  (n= ) | AQ/AS3  (n= ) |
| --- | --- | --- | --- | --- |
| Events | XX | XX | XX | XX |
| PYAR | XX.X | XX.X | XX.X | XX.X |
| Rate | X.XX | X.XX | X.XX | X.XX |
| PE (95% CI) | Reference group | XX.X  (.X-.X%) | XX.X  (.X-.X%) | XX.X  (.X-.X%) |
| p-value | - | X.XXX | X.XXX | X.XXX |

Event: total number of malaria episodes, for parasites > 0, PYAR=person years at risk, PE=protective efficacy

Scope: from dose 1 until age 12 months

***Values of first or only episode of anemia***

### Anemia

#### Primary definition of anemia (moderate-to-severe)

An anemia episode is defined as a child with a hemoglobin level (Hb) below 8.0 g/dl at any contact with the healthcare system, whether at passive visits or follow-up study visits. For any illness episode, the lowest Hb value associated with that illness shall determine whether the child meets the criterion for inclusion in that group. For example, if a child has an Hb of 8.5 at a sick visit, but is hospitalized and the Hb measurement upon admission is 7.9, then the child should be included as having met the criteria for an episode of moderate-to-severe anemia.

#### Alternate definition 1 of anemia (mild)

Same as primary definition, but with threshold below 11.0 g/dl

#### Alternate definition 2 of anemia (critical)

Same as primary definition, but with threshold below 5.0 g/dl

**Post-dose anemia**

The incidence of anemia (mild, moderate-to-severe, and critical) within a one month period after each course of study drug.

Table X. Distribution of first or only episode of anemia according to treatment group

| Events | Treatment | | | |
| --- | --- | --- | --- | --- |
|  | Placebo | | SP/AS3 | |
| Yes | XXX | XX.X% | XXX | XX.X% |
| No | XXX | XX.X% | XXX | XX.X% |
| Total | XXX | 100.0% | XXX | 100.0% |

Event: first episode of anemia, for Hb < 8.0 g/dl

Scope: from dose 1 until age 12 months

| Events | Treatment | | | |
| --- | --- | --- | --- | --- |
|  | Placebo | | LAPDAP | |
| Yes | XXX | XX.X% | XXX | XX.X% |
| No | XXX | XX.X% | XXX | XX.X% |
| Total | XXX | 100.0% | XXX | 100.0% |

| Events | Treatment | | | |
| --- | --- | --- | --- | --- |
|  | Placebo | | AQ/AS3 | |
| Yes | XXX | XX.X% | XXX | XX.X% |
| No | XXX | XX.X% | XXX | XX.X% |
| Total | XXX | 100.0% | XXX | 100.0% |

Table X. Person-Years at Risk (PYAR) Ratio

|  | Placebo  (n= ) | SP/AS3  (n= ) | LAPDAP  (n= ) | AQ/AS3  (n= ) |
| --- | --- | --- | --- | --- |
| Events | XX | XX | XX | XX |
| PYAR | XX.X | XX.X | XX.X | XX.X |
| Rate | X.XX | X.XX | X.XX | X.XX |
| PE (95% CI) | Reference group | XX.X  (.X-.X%) | XX.X  (.X-.X%) | XX.X  (.X-.X%) |
| p-value | - | X.XXX | X.XXX | X.XXX |

Event: first or only episode of anemia, for Hb < 8.0 g/dl

Scope: from dose 1 until age 12 months

Note: p-value by LR-test

Table X. Evaluation of potential confounding variables (15% level)

| Potential confounding variable | Adjusted treatment efficacy | p-value | Relative difference, % |
| --- | --- | --- | --- |
| None (unadjusted estimate) | XX.X% | X.XXX |  |
| Sex | XX.X% | X.XXX | XX.X |
| Age (months) at dose 1 | XX.X% | X.XXX | XX.X |
| Z-score: Height-for-age | XX.X% | X.XXX | XX.X |
| Z-score: Weight-for-age | XX.X% | X.XXX | XX.X |
| Z-score: Weight-for-height | XX.X% | X.XXX | XX.X |
| IPTi clinic | XX.X% | X.XXX | XX.X |
| G6PD status |  |  |  |
| Hb genotype |  |  |  |
| Vomiting |  |  |  |
| Presumptive quinine treatment |  |  |  |
| ITN use |  |  |  |
| No / Yes, confounding variables exist | | | |

Event: first episode of anemia, for Hb < 8.0 g/dl

Scope: from dose 1 until age 12 months

Table X. Risk Factors. Univariate Analysis

| Variable | | Hazard Ratio | (95%CI) | p-value |
| --- | --- | --- | --- | --- |
| Sex | Male | 1 |  | X.XXX |
|  | Female | X.XX | (X.XX; X.XX) |  |
| IPTi clinic | 01 | 1 |  | X.XXX |
|  | 02 | X.XX | (X.XX; X.XX) |  |
|  | ... | X.XX | (X.XX; X.XX) |  |
| Age (months) at dose 1 | for unit increase | X.XX | (X.XX; X.XX) | X.XXX |
| Z-score: Height-for-age | for unit increase | X.XX | (X.XX; X.XX) | X.XXX |
| Z-score: Weight-for-age | for unit increase | X.XX | (X.XX; X.XX) | X.XXX |
| Z-score: Weight-for-height | for unit increase | X.XX | (X.XX; X.XX) | X.XXX |
| … |  |  |  |  |

P-values by Wald test

Event: first episode of anemia, for Hb < 8.0 g/dl

Scope: from dose 1 until age 12 months

***Per-protocol analysis***

The analysis will be the same as described above but will be restricted to children who completed all the three courses of study drug as per-protocol.

***Prevalence of parasitaemia and anemia (mild, moderate-to-severe, and critical) at 12 months of age***

The prevalence of asexual *P falciparum* parasitaemia, the prevalence of anemia and the mean hemoglobin levels will be assessed. This evaluation will be conducted on data collected from children aged 12 months old.

***Anthropometric Indices***

Anthropometric indices will be assessed by comparing the proportions of children in each drug regimen and placebo with a weight for age Z-score < -2 and the proportion of children with a height for age Z-score < -2. This assessment will be performed on data collected at 12 months of age.

Table X. Prevalence of malaria and anemia at 12 months of age

| Variable | | Placebo  (XXX) | | SP/AS3  (XXX) | | LAPDAP  (XXX) | | AQ/AS3  (XXX) | |
| --- | --- | --- | --- | --- | --- | --- | --- | --- | --- |
|  |  | n | % | n | % | n | % | n | % |
| parasitaemia | Yes | XXX | XX% | XXX | XX% | XXX | XX% | XXX | XX% |
|  | No | XXX | XX% | XXX | XX% | XXX | XX% | XXX | XX% |
| Anemia | ≥ 11.0g/dL | XXX | XX% | XXX | XX% | XXX | XX% | XXX | XX% |
|  | < 11.0g/dL |  |  |  |  |  |  |  |  |
|  | < 8.0g/dL |  |  |  |  |  |  |  |  |
|  | < 5.0g/dL | XXX | XX% | XXX | XX% | XXX | XX% | XXX | XX% |
| Mean Hb levels | |  |  |  |  |  |  |  |  |
| Z-score: Height for age | | XXX | XX% | XXX | XX% | XXX | XX% | XXX | XX% |
| … | |  |  |  |  |  |  |  |  |

## Safety of IPTi

***Vomiting***

The frequency of vomiting the different drug regimens will be presented.

Table X. Vomiting when dose 1 was taken

| Vomiting after dose 1 | Treatment | | | | | | | | Total | |
| --- | --- | --- | --- | --- | --- | --- | --- | --- | --- | --- |
|  | Placebo | | SP/AS3 | | LAPDAP | | AQ/AS3 | |  |  |
| No | XXX | XX% | XXX | XX% | XXX | XX% | XXX | XX% | XXX | XX% |
| Vomit attempt1-day1 | XXX | XX% | XXX | XX% | XXX | XX% | XXX | XX% | XXX | XX% |
| Vomit repeat-day1 | XXX | XX% | XXX | XX% | XXX | XX% | XXX | XX% | XXX | XX% |
| Vomit attempt1-day2 | XXX | XX% | XXX | XX% | XXX | XX% | XXX | XX% | XXX | XX% |
| Vomit repeat-day2 | XXX | XX% | XXX | XX% | XXX | XX% | XXX | XX% | XXX | XX% |
| Vomit attempt1-day3 | XXX | XX% | XXX | XX% | XXX | XX% | XXX | XX% | XXX | XX% |
| Vomit repeat-day3 | XXX | XX% | XXX | XX% | XXX | XX% | XXX | XX% | XXX | XX% |
| Total | XXX | XX% | XXX | XX% | XXX | XX% | XXX | XX% | XXX | XX% |

Table X. Vomiting when dose 2 was taken

| Vomiting after dose 2 | Treatment | | | | | | | | Total | |
| --- | --- | --- | --- | --- | --- | --- | --- | --- | --- | --- |
|  | Placebo | | SP/AS3 | | LAPDAP | | AQ/AS3 | |  |  |
| No | XXX | XX% | XXX | XX% | XXX | XX% | XXX | XX% | XXX | XX% |
| Vomit attempt1-day1 | XXX | XX% | XXX | XX% | XXX | XX% | XXX | XX% | XXX | XX% |
| Vomit repeat -day1 | XXX | XX% | XXX | XX% | XXX | XX% | XXX | XX% | XXX | XX% |
| Vomit attempt1-day2 | XXX | XX% | XXX | XX% | XXX | XX% | XXX | XX% | XXX | XX% |
| Vomit repeat -day2 | XXX | XX% | XXX | XX% | XXX | XX% | XXX | XX% | XXX | XX% |
| Vomit attempt1-day3 | XXX | XX% | XXX | XX% | XXX | XX% | XXX | XX% | XXX | XX% |
| Vomit repeat -day3 | XXX | XX% | XXX | XX% | XXX | XX% | XXX | XX% | XXX | XX% |
| Total | XXX | XX% | XXX | XX% | XXX | XX% | XXX | XX% | XXX | XX% |

Table X. Vomiting when dose 3 was taken

| Vomiting after dose 3 | Treatment | | | | | | | | Total | |
| --- | --- | --- | --- | --- | --- | --- | --- | --- | --- | --- |
|  | Placebo | | SP/AS3 | | LAPDAP | | AQ/AS3 | |  |  |
| No | XXX | XX% | XXX | XX% | XXX | XX% | XXX | XX% | XXX | XX% |
| Vomit attempt1-day1 | XXX | XX% | XXX | XX% | XXX | XX% | XXX | XX% | XXX | XX% |
| Vomit repeat -day1 | XXX | XX% | XXX | XX% | XXX | XX% | XXX | XX% | XXX | XX% |
| Vomit attempt1-day2 | XXX | XX% | XXX | XX% | XXX | XX% | XXX | XX% | XXX | XX% |
| Vomit repeat -day2 | XXX | XX% | XXX | XX% | XXX | XX% | XXX | XX% | XXX | XX% |
| Vomit attempt1-day3 | XXX | XX% | XXX | XX% | XXX | XX% | XXX | XX% | XXX | XX% |
| Vomit repeat -day3 | XXX | XX% | XXX | XX% | XXX | XX% | XXX | XX% | XXX | XX% |
| Total | XXX | XX% | XXX | XX% | XXX | XX% | XXX | XX% | XXX | XX% |

Table X. Compliance with dose administration schedule

| Administration of  given treatment | Treatment | | | | | | | | Total | |
| --- | --- | --- | --- | --- | --- | --- | --- | --- | --- | --- |
|  | Placebo | | SP/AS3 | | LAPDAP | | AQ/AS3 | |  |  |
| OK – NO – NO | XXX | XX% | XXX | XX% | XXX | XX% | XXX | XX% | XXX | XX% |
| OK – OK – NO | XXX | XX% | XXX | XX% | XXX | XX% | XXX | XX% | XXX | XX% |
| OK – OK – OK | XXX | XX% | XXX | XX% | XXX | XX% | XXX | XX% | XXX | XX% |
| OK – NO – OK | XXX | XX% | XXX | XX% | XXX | XX% | XXX | XX% | XXX | XX% |
| Total | XXX | XX% | XXX | XX% | XXX | XX% | XXX | XX% | XXX | XX% |

OK = means that the full course of that dose of study drug was successfully administered.

NO = means that the full course of that dose of study drug was completely vomited or missed.

OK – OK – OK = means that all 3 courses of study drug were administered per-protocol.

OK – NO – NO = means that only 1 course of study drug was successfully administered.

OK – OK – NO = means that 2 courses of study drug were successfully administered.

### Deaths

The total number of deaths by treatment group will be presented.

Table X. Description of number of deaths by group

| Variable | Placebo  (XXX) | | SP/AS3  (XXX) | | LAPDAP  (XXX) | | AQ/AS3  (XXX) | | Total |
| --- | --- | --- | --- | --- | --- | --- | --- | --- | --- |
|  | n | % | n | % | n | % | n | % |  |
| Dead | XXX | XX% | XXX | XX% | XXX | XX% | XXX | XX% | X.XXX^1,2^ |

### Dermatological adverse events

Table X. Descriptor of number of adverse events and infants with AE’s

| Variable | Obs | Mean | SD | Min | Max |
| --- | --- | --- | --- | --- | --- |
| Number of dermatologic AE’s | XXX | XX | XX.X | XX | XX |
| Number of infants with at least one dermatologic AE | XXX | XX | XX.X | XX | XX |
| Stevens-Johnson syndrome |  |  |  |  |  |

Table X. Distribution of dermatologic adverse events according to treatment group

| Variable | | Placebo  (XXX) | | SP/AS3  (XXX) | | LAPDAP  (XXX) | | AQ/AS3  (XXX) | | p-value |
| --- | --- | --- | --- | --- | --- | --- | --- | --- | --- | --- |
|  |  | n | % | n | % | n | % | n | % |  |
| Stomatitis | Yes | XXX | XX% | XXX | XX% | XXX | XX% | XXX | XX% | X.XXX^1,2^ |
|  | No | XXX | XX% | XXX | XX% | XXX | XX% | XXX | XX% |  |
| Itching | Yes | XXX | XX% | XXX | XX% | XXX | XX% | XXX | XX% | X.XXX^1,2^ |
|  | No | XXX | XX% | XXX | XX% | XXX | XX% | XXX | XX% |  |
| Skin rash | Yes | XXX | XX% | XXX | XX% | XXX | XX% | XXX | XX% | X.XXX^1,2^ |
|  | No | XXX | XX% | XXX | XX% | XXX | XX% | XXX | XX% |  |
| Erythema | Yes | XXX | XX% | XXX | XX% | XXX | XX% | XXX | XX% | X.XXX^1,2^ |
|  | No | XXX | XX% | XXX | XX% | XXX | XX% | XXX | XX% |  |
| Diffuse papular eruption | Yes | XXX | XX% | XXX | XX% | XXX | XX% | XXX | XX% | X.XXX^1,2^ |
|  | No | XXX | XX% | XXX | XX% | XXX | XX% | XXX | XX% |  |
| Peeling | Yes | XXX | XX% | XXX | XX% | XXX | XX% | XXX | XX% | X.XXX^1,2^ |
|  | No | XXX | XX% | XXX | XX% | XXX | XX% | XXX | XX% |  |
| Blisters, ulcers | Yes | XXX | XX% | XXX | XX% | XXX | XX% | XXX | XX% | X.XXX^1,2^ |
|  | No | XXX | XX% | XXX | XX% | XXX | XX% | XXX | XX% |  |
| Wet peeling | Yes | XXX | XX% | XXX | XX% | XXX | XX% | XXX | XX% | X.XXX^1,2^ |
|  | No | XXX | XX% | XXX | XX% | XXX | XX% | XXX | XX% |  |
| Urticaria | Yes | XXX | XX% | XXX | XX% | XXX | XX% | XXX | XX% | X.XXX^1,2^ |
|  | No | XXX | XX% | XXX | XX% | XXX | XX% | XXX | XX% |  |
| Wheezing | Yes | XXX | XX% | XXX | XX% | XXX | XX% | XXX | XX% | X.XXX^1,2^ |
|  | No | XXX | XX% | XXX | XX% | XXX | XX% | XXX | XX% |  |
| Needs hospitalization | Yes | XXX | XX% | XXX | XX% | XXX | XX% | XXX | XX% | X.XXX^1,2^ |
|  | No | XXX | XX% | XXX | XX% | XXX | XX% | XXX | XX% |  |
| Hospitalization skin problems | Yes | XXX | XX% | XXX | XX% | XXX | XX% | XXX | XX% | X.XXX^1,2^ |
|  | No | XXX | XX% | XXX | XX% | XXX | XX% | XXX | XX% |  |

1: Fisher’s test

2: Chi-square test

Table X. Distribution of number of dermatologic AEs and infants with at least one dermatologic AE, by treatment group

| Variable | Treatment | | | | | | | | | | | | | | | |
| --- | --- | --- | --- | --- | --- | --- | --- | --- | --- | --- | --- | --- | --- | --- | --- | --- |
|  | Placebo  (XXX) | | | SP/AS3  (XXX) | | | | LAPDAP  (XXX) | | | | AQ/AS3  (XXX) | | | TOTAL  (XXX) | |
|  | Mean | SD | | Mean | | SD | | Mean | | SD | | Mean | | SD | Mean | SD |
| Number of dermatologic AEs | XX.X | | XX.X | | XX.X | | XX.X | | XX.X | | XX.X | | XX.X | XX.X | XX.X | XX.X |
| Number of infants with at least one dermatologic AEs | XX.X | XX.X | | XX.X | | XX.X | | XX.X | | XX.X | | XX.X | | XX.X | XX.X | XX.X |
| Stevens-Johnson syndrome |  |  | |  | |  | |  | |  | |  | |  |  |  |

#### *Outpatient visits (all cause and separately malaria and anemia related)*

Table X. Person-Years at Risk (PYAR) Ratio

|  | Placebo  (n= ) | SP/AS3  (n= ) | LAPDAP  (n= ) | AQ/AS3  (n= ) |
| --- | --- | --- | --- | --- |
| Events | XX | XX | XX | XX |
| PYAR | XX.X | XX.X | XX.X | XX.X |
| Rate | X.XX | X.XX | X.XX | X.XX |
| PE (95% CI) | Reference group | XX.X  (.X-.X%) | XX.X  (.X-.X%) | XX.X  (.X-.X%) |
| p-value | - | X.XXX | X.XXX | X.XXX |

Event: OPD visits (all cause or disease specific)

Scope: from dose 1 until age 12 months

Note: p-value by LR-test

Table X. Distribution of the number of OPD visits according to treatment group

| Variable | | Placebo  (XXX) | | SP/AS3  (XXX) | | LAPDAP  (XXX) | | AQ/AS3  (XXX) | |
| --- | --- | --- | --- | --- | --- | --- | --- | --- | --- |
|  |  | n | % | n | % | n | % | n | % |
| OPD visits | 1 | XXX | XX% | XXX | XX% | XXX | XX% | XXX | XX% |
|  | … | XXX | XX% | XXX | XX% | XXX | XX% | XXX | XX% |
|  | n | XXX | XX% | XXX | XX% | XXX | XX% | XXX | XX% |

Table X. Distribution of OPD diagnoses according to treatment group

| Variable | | Placebo  (XXX) | | SP/AS3  (XXX) | | LAPDAP  (XXX) | | AQ/AS3  (XXX) | | p-value |
| --- | --- | --- | --- | --- | --- | --- | --- | --- | --- | --- |
|  |  | n | % | n | % | n | % | n | % |  |
| URTI | Yes | XXX | XX% | XXX | XX% | XXX | XX% | XXX | XX% | X.XXX^1,2^ |
|  | No | XXX | XX% | XXX | XX% | XXX | XX% | XXX | XX% |  |
| Pneumonia | Yes | XXX | XX% | XXX | XX% | XXX | XX% | XXX | XX% | X.XXX^1,2^ |
|  | No | XXX | XX% | XXX | XX% | XXX | XX% | XXX | XX% |  |
| Gastroenteritis | Yes | XXX | XX% | XXX | XX% | XXX | XX% | XXX | XX% | X.XXX^1,2^ |
|  | No | XXX | XX% | XXX | XX% | XXX | XX% | XXX | XX% |  |
| Diarrhea | Yes | XXX | XX% | XXX | XX% | XXX | XX% | XXX | XX% | X.XXX^1,2^ |
|  | No | XXX | XX% | XXX | XX% | XXX | XX% | XXX | XX% |  |
| ARI | Yes | XXX | XX% | XXX | XX% | XXX | XX% | XXX | XX% | X.XXX^1,2^ |
|  | No | XXX | XX% | XXX | XX% | XXX | XX% | XXX | XX% |  |
| … | Yes | XXX | XX% | XXX | XX% | XXX | XX% | XXX | XX% | X.XXX^1,2^ |
|  | No | XXX | XX% | XXX | XX% | XXX | XX% | XXX | XX% |  |

1: Fisher test

2: Chi-square test

Table X. Distribution of OPD symptoms according to treatment group

| Variable | | Placebo  (XXX) | | SP/AS3  (XXX) | | LAPDAP  (XXX) | | AQ/AS3  (XXX) | | p-value |
| --- | --- | --- | --- | --- | --- | --- | --- | --- | --- | --- |
|  |  | n | % | n | % | n | % | n | % |  |
| Cough | Yes | XXX | XX% | XXX | XX% | XXX | XX% | XXX | XX% | X.XXX^1,2^ |
|  | No | XXX | XX% | XXX | XX% | XXX | XX% | XXX | XX% |  |
| Difficulty breathing | Yes | XXX | XX% | XXX | XX% | XXX | XX% | XXX | XX% | X.XXX^1,2^ |
|  | No | XXX | XX% | XXX | XX% | XXX | XX% | XXX | XX% |  |
| Diarrhea | Yes | XXX | XX% | XXX | XX% | XXX | XX% | XXX | XX% | X.XXX^1,2^ |
|  | No | XXX | XX% | XXX | XX% | XXX | XX% | XXX | XX% |  |
| Skinfold | Yes | XXX | XX% | XXX | XX% | XXX | XX% | XXX | XX% | X.XXX^1,2^ |
|  | No | XXX | XX% | XXX | XX% | XXX | XX% | XXX | XX% |  |
| Vomiting | Yes | XXX | XX% | XXX | XX% | XXX | XX% | XXX | XX% | X.XXX^1,2^ |
|  | No | XXX | XX% | XXX | XX% | XXX | XX% | XXX | XX% |  |
| Fontanel | Yes | XXX | XX% | XXX | XX% | XXX | XX% | XXX | XX% | X.XXX^1,2^ |
|  | No | XXX | XX% | XXX | XX% | XXX | XX% | XXX | XX% |  |
| Dehydration | Yes | XXX | XX% | XXX | XX% | XXX | XX% | XXX | XX% | X.XXX^1,2^ |
|  | No | XXX | XX% | XXX | XX% | XXX | XX% | XXX | XX% |  |
| Pallor | Yes | XXX | XX% | XXX | XX% | XXX | XX% | XXX | XX% | X.XXX^1,2^ |
|  | No | XXX | XX% | XXX | XX% | XXX | XX% | XXX | XX% |  |
| Jaundice | Yes | XXX | XX% | XXX | XX% | XXX | XX% | XXX | XX% | X.XXX^1,2^ |
|  | No | XXX | XX% | XXX | XX% | XXX | XX% | XXX | XX% |  |
| Edema | Yes | XXX | XX% | XXX | XX% | XXX | XX% | XXX | XX% | X.XXX^1,2^ |
|  | No | XXX | XX% | XXX | XX% | XXX | XX% | XXX | XX% |  |
| Ear discharge | Yes | XXX | XX% | XXX | XX% | XXX | XX% | XXX | XX% | X.XXX^1,2^ |
|  | No | XXX | XX% | XXX | XX% | XXX | XX% | XXX | XX% |  |
| Indrawing | Yes | XXX | XX% | XXX | XX% | XXX | XX% | XXX | XX% | X.XXX^1,2^ |
|  | No | XXX | XX% | XXX | XX% | XXX | XX% | XXX | XX% |  |
| Nasal flaring | Yes | XXX | XX% | XXX | XX% | XXX | XX% | XXX | XX% | X.XXX^1,2^ |
|  | No | XXX | XX% | XXX | XX% | XXX | XX% | XXX | XX% |  |
| Crepitant rales | Yes | XXX | XX% | XXX | XX% | XXX | XX% | XXX | XX% | X.XXX^1,2^ |
|  | No | XXX | XX% | XXX | XX% | XXX | XX% | XXX | XX% |  |
| Wheezing | Yes | XXX | XX% | XXX | XX% | XXX | XX% | XXX | XX% | X.XXX^1,2^ |
|  | No | XXX | XX% | XXX | XX% | XXX | XX% | XXX | XX% |  |
| Hepatomegaly | Yes | XXX | XX% | XXX | XX% | XXX | XX% | XXX | XX% | X.XXX^1,2^ |
|  | No | XXX | XX% | XXX | XX% | XXX | XX% | XXX | XX% |  |
| Splenomegalia | Yes | XXX | XX% | XXX | XX% | XXX | XX% | XXX | XX% | X.XXX^1,2^ |
|  | No | XXX | XX% | XXX | XX% | XXX | XX% | XXX | XX% |  |
| Stiff neck | Yes | XXX | XX% | XXX | XX% | XXX | XX% | XXX | XX% | X.XXX^1,2^ |
|  | No | XXX | XX% | XXX | XX% | XXX | XX% | XXX | XX% |  |
| Lethargy | Yes | XXX | XX% | XXX | XX% | XXX | XX% | XXX | XX% | X.XXX^1,2^ |
|  | No | XXX | XX% | XXX | XX% | XXX | XX% | XXX | XX% |  |
| Seizures | Yes | XXX | XX% | XXX | XX% | XXX | XX% | XXX | XX% | X.XXX^1,2^ |
|  | No | XXX | XX% | XXX | XX% | XXX | XX% | XXX | XX% |  |

1: Fisher’s test

2: Chi-square test

Table X. Person-years at Risk (PYAR) Ratio

|  | Placebo  (n= ) | SP/AS3  (n= ) | LAPDAP  (n= ) | AQ/AS3  (n= ) |
| --- | --- | --- | --- | --- |
| Events | XX | XX | XX | XX |
| PYAR | XX.X | XX.X | XX.X | XX.X |
| Rate | X.XX | X.XX | X.XX | X.XX |
| PE (95% CI) | Reference group | XX.X  (.X-.X%) | XX.X  (.X-.X%) | XX.X  (.X-.X%) |
| p-value | - | X.XXX | X.XXX | X.XXX |

Event: e.g. difficulty breathing (OPD)

Scope: from dose 1 until age 12 months

Note: p-value by LR-test

#### Hospitalizations (all-cause and malaria and anemia related)

Table X. Person-Years at Risk (PYAR) Ratio

|  | Placebo  (n= ) | SP/AS3  (n= ) | LAPDAP  (n= ) | AQ/AS3  (n= ) |
| --- | --- | --- | --- | --- |
| Events | XX | XX | XX | XX |
| PYAR | XX.X | XX.X | XX.X | XX.X |
| Rate | X.XX | X.XX | X.XX | X.XX |
| PE (95% CI) | Reference group | XX.X  (.X-.X%) | XX.X  (.X-.X%) | XX.X  (.X-.X%) |
| p-value | - | X.XXX | X.XXX | X.XXX |

Event: hospitalizations (all-cause and malaria and anemia related)

Scope: from dose 1 until age 12 months

Note: p-value by LR-test

Table X. Distribution of the number of all cause hospitalizations according to treatment group

1: Fisher’s test

| Variable | | Placebo  (XXX) | | SP/AS3  (XXX) | | LAPDAP  (XXX) | | AQ/AS3  (XXX) | | p-value |
| --- | --- | --- | --- | --- | --- | --- | --- | --- | --- | --- |
|  |  | n | % | n | % | n | % | n | % |  |
| IPD visits | 1 | XXX | XX% | XXX | XX% | XXX | XX% | XXX | XX% | X.XXX^1,2^ |
|  | 2 | XXX | XX% | XXX | XX% | XXX | XX% | XXX | XX% |  |
|  | … | XXX | XX% | XXX | XX% | XXX | XX% | XXX | XX% |  |
|  | n | XXX | XX% | XXX | XX% | XXX | XX% | XXX | XX% |  |

2: Chi-square test

Table X. Distribution of hospitalization diagnoses according to treatment group

| Variable | | Placebo  (XXX) | | SP/AS3  (XXX) | | LAPDAP  (XXX) | | AQ/AS3  (XXX) | | p-value |
| --- | --- | --- | --- | --- | --- | --- | --- | --- | --- | --- |
|  |  | n | % | n | % | n | % | n | % |  |
| URTI | Yes | XXX | XX% | XXX | XX% | XXX | XX% | XXX | XX% | X.XXX^1,2^ |
|  | No | XXX | XX% | XXX | XX% | XXX | XX% | XXX | XX% |  |
| Pneumonia | Yes | XXX | XX% | XXX | XX% | XXX | XX% | XXX | XX% | X.XXX^1,2^ |
|  | No | XXX | XX% | XXX | XX% | XXX | XX% | XXX | XX% |  |
| Gastroenteritis | Yes | XXX | XX% | XXX | XX% | XXX | XX% | XXX | XX% | X.XXX^1,2^ |
|  | No | XXX | XX% | XXX | XX% | XXX | XX% | XXX | XX% |  |
| Diarrhea | Yes | XXX | XX% | XXX | XX% | XXX | XX% | XXX | XX% | X.XXX^1,2^ |
|  | No | XXX | XX% | XXX | XX% | XXX | XX% | XXX | XX% |  |
| ARI | Yes | XXX | XX% | XXX | XX% | XXX | XX% | XXX | XX% | X.XXX^1,2^ |
|  | No | XXX | XX% | XXX | XX% | XXX | XX% | XXX | XX% |  |
| … | Yes | XXX | XX% | XXX | XX% | XXX | XX% | XXX | XX% | X.XXX^1,2^ |
|  | No | XXX | XX% | XXX | XX% | XXX | XX% | XXX | XX% |  |

1: Fisher test

2: Chi-square test

Table X. Distribution of hospitalization symptoms according to treatment group

| Variable | | Placebo  (XXX) | | | SP/AS3  (XXX) | | LAPDAP  (XXX) | | AQ/AS3  (XXX) | | p-value |
| --- | --- | --- | --- | --- | --- | --- | --- | --- | --- | --- | --- |
|  |  | n | | % | n | % | n | % | n | % |  |
| Cough | Yes | XXX | | XX% | XXX | XX% | XXX | XX% | XXX | XX% | X.XXX^1,2^ |
|  | No | XXX | | XX% | XXX | XX% | XXX | XX% | XXX | XX% |  |
| Difficulty breathing | Yes | XXX | | XX% | XXX | XX% | XXX | XX% | XXX | XX% | X.XXX^1,2^ |
|  | No | XXX | | XX% | XXX | XX% | XXX | XX% | XXX | XX% |  |
| Diarrhea | Yes | XXX | | XX% | XXX | XX% | XXX | XX% | XXX | XX% | X.XXX^1,2^ |
|  | No | XXX | | XX% | XXX | XX% | XXX | XX% | XXX | XX% |  |
| Skinfold | Yes | XXX | | XX% | XXX | XX% | XXX | XX% | XXX | XX% | X.XXX^1,2^ |
|  | No | XXX | | XX% | XXX | XX% | XXX | XX% | XXX | XX% |  |
| Vomiting | Yes | XXX | | XX% | XXX | XX% | XXX | XX% | XXX | XX% | X.XXX^1,2^ |
|  | No | XXX | | XX% | XXX | XX% | XXX | XX% | XXX | XX% |  |
| Fontanel | Yes | XXX | | XX% | XXX | XX% | XXX | XX% | XXX | XX% | X.XXX^1,2^ |
|  | No | XXX | | XX% | XXX | XX% | XXX | XX% | XXX | XX% |  |
| Dehydration | Yes | XXX | | XX% | XXX | XX% | XXX | XX% | XXX | XX% | X.XXX^1,2^ |
|  | No | XXX | | XX% | XXX | XX% | XXX | XX% | XXX | XX% |  |
| Pallor | Yes | XXX | | XX% | XXX | XX% | XXX | XX% | XXX | XX% | X.XXX^1,2^ |
|  | No | XXX | | XX% | XXX | XX% | XXX | XX% | XXX | XX% |  |
| Jaundice | Yes | XXX | | XX% | XXX | XX% | XXX | XX% | XXX | XX% | X.XXX^1,2^ |
|  | No | XXX | | XX% | XXX | XX% | XXX | XX% | XXX | XX% |  |
| Edema | Yes | XXX | | XX% | XXX | XX% | XXX | XX% | XXX | XX% | X.XXX^1,2^ |
|  | No | XXX | | XX% | XXX | XX% | XXX | XX% | XXX | XX% |  |
| Ear discharge | Yes | XXX | | XX% | XXX | XX% | XXX | XX% | XXX | XX% | X.XXX^1,2^ |
|  | No | XXX | | XX% | XXX | XX% | XXX | XX% | XXX | XX% |  |
| Indrawing | Yes | XXX | XX% | | XXX | XX% | XXX | XX% | XXX | XX% | X.XXX^1,2^ |
|  | No | XXX | XX% | | XXX | XX% | XXX | XX% | XXX | XX% |  |
| Nasal flaring | Yes | XXX | XX% | | XXX | XX% | XXX | XX% | XXX | XX% | X.XXX^1,2^ |
|  | No | XXX | XX% | | XXX | XX% | XXX | XX% | XXX | XX% |  |
| Crepitant rales | Yes | XXX | XX% | | XXX | XX% | XXX | XX% | XXX | XX% | X.XXX^1,2^ |
|  | No | XXX | XX% | | XXX | XX% | XXX | XX% | XXX | XX% |  |
| Wheezing | Yes | XXX | XX% | | XXX | XX% | XXX | XX% | XXX | XX% | X.XXX^1,2^ |
|  | No | XXX | XX% | | XXX | XX% | XXX | XX% | XXX | XX% |  |
| Hepatomegaly | Yes | XXX | XX% | | XXX | XX% | XXX | XX% | XXX | XX% | X.XXX^1,2^ |
|  | No | XXX | XX% | | XXX | XX% | XXX | XX% | XXX | XX% |  |
| Splenomegalia | Yes | XXX | XX% | | XXX | XX% | XXX | XX% | XXX | XX% | X.XXX^1,2^ |
|  | No | XXX | XX% | | XXX | XX% | XXX | XX% | XXX | XX% |  |
| Stiff neck | Yes | XXX | XX% | | XXX | XX% | XXX | XX% | XXX | XX% | X.XXX^1,2^ |
|  | No | XXX | XX% | | XXX | XX% | XXX | XX% | XXX | XX% |  |
| Lethargy | Yes | XXX | XX% | | XXX | XX% | XXX | XX% | XXX | XX% | X.XXX^1,2^ |
|  | No | XXX | XX% | | XXX | XX% | XXX | XX% | XXX | XX% |  |
| Seizures | Yes | XXX | | XX% | XXX | XX% | XXX | XX% | XXX | XX% | X.XXX^1,2^ |
|  | No | XXX | | XX% | XXX | XX% | XXX | XX% | XXX | XX% |  |

1: Fisher test

2: Chi-square test

## D Other sub-analyses

Repeat analysis B for children who met the primary outcome at IPTi visits

Assess compliance of delivering IPTi concurrently with EPI schedule

Appendix 1

xxxx

Infants randomized

xxxx

Assigned to placebo

xxxx

Assigned to one of 3 treatments

xxxx

Receive 1st dose

xxxx

Receive 1st dose

xxxx

xxxx

xxxx

deaths migrations

withdrawals

xxxx

do not receive 2nd dose or 3rd dose

xxxx

do not receive 2nd dose

xxxx

receive 2nd dose

xxxx

xxxx

xxxx

deaths migrations

withdrawals

xxxx

xxxx

xxxx

deaths migrations

withdrawals

xxxx

do not receive 2nd dose or 3rd dose

xxxx

do not receive 2nd dose

xxxx

receive 2nd dose

xxxx

receive 3rd dose

xxxx

Followed up until age 1 year

xxxx

do not receive 3rd dose

xxxx

xxxx

xxxx

deaths migrations

withdrawals

xxxx

xxxx

xxxx

deaths migrations

withdrawals

xxxx

xxxx

xxxx

deaths migrations

withdrawals

xxxx

receive 3rd dose

xxxx

do not receive 3rd dose

xxxx

Followed up until age 1 year

Appendix 2

xxxx

infants randomized

xxxx

assigned to placebo

xxxx

receive 1st dose

xxxx

xxxx

xxxx

deaths migrations

withdrawals

xxxx

do not receive 2nd dose

xxxx

receive 2nd dose

xxxx

xxxx

xxxx

deaths migrations

withdrawals

xxxx

receive 3rd dose

xxxx

Followed up until age 1 year

xxxx

do not receive 3rd dose

xxxx

xxxx

xxxx

deaths migrations

withdrawals

xxxx

assigned to one of 3 treatments

xxxx

receive 1st dose

xxxx

receive 2nd dose

xxxx

receive 3rd dose

xxxx

Followed up until age 1 year

xxxx

xxxx

xxxx

deaths migrations

withdrawals

xxxx

do not receive 2nd dose

xxxx

xxxx

xxxx

deaths migrations

withdrawals

xxxx

do not receive 3rd dose

xxxx

xxxx

xxxx

deaths migrations

withdrawals
