# Supplementary material for: Intermittent Preventive Treatment in Infants for the Prevention of Malaria in Rural Western Kenya: A Randomized, Double-Blind Placebo-Controlled Trial
Source: PLoS One. 2010 Apr 2;5(4):e10016. doi: 10.1371/journal.pone.0010016 (PMC2848869; doi:10.1371/journal.pone.0010016)
Supplement: Protocol S1 — Trial Protocol (0.27 MB DOC) [file pone.0010016.s003.doc]

**Efficacy and safety of pediatric immunization-linked preventive intermittent treatment with antimalarials in decreasing anemia and malaria morbidity in rural western Kenya**

**Investigators**

## Centers for Disease Control and Prevention - Atlanta

Dr. Robert D. Newman

Dr. Mary Hamel

Dr. Daniel Feikin

Dr. Richard W. Steketee

Ms. Lauren Singer

Ms. Elizabeth Peterson

Dr. Feiko ter Kuile

Dr. John Williamson

Dr. Meghna Desai

Ms. Annett Hoppe

CDC Kenya

Dr. Laurence Slutsker

Dr. Kim Lindblade

Dr. Ya Ping Shi

## Kenya Medical Research Institute

Dr. John M. Vulule

Mr. Frank Odhiambo

Ms. Jane Alaii

## Kenya Ministry of Health

Mr. Amos Odhacha

**Role of CDC Investigators**

# Dr. Robert D. Newman will serve as the co-principal investigator along with Dr. Laurence Slutsker. They will be responsible for overall design of the trial, management, data collection and data analysis. Dr. Mary Hamel will advise on development of study protocol, study training, supervision of the study personnel, analysis and interpretation of data. Dr. Daniel Feikin will advise on development of study protocol with regard to the impact of routine antimalarial usage on response to the conjugate vaccine against *Haemophilus influenzae* type b. Dr. Richard Steketee will advise on development of the study protocol, analysis and interpretation of data. Dr. Kim Lindblade will advise on development of study protocol and assist with supervision of field staff, analysis and interpretation of data. Dr. Ya Ping Shi will oversee laboratory testing and quality control. Ms. Lauren Singer will help with study preparations and protocol development. Ms. Elizabeth Peterson will help with study preparations, staff training, and will provide day-to-day supervision of field staff. Dr. Feiko ter Kuile will assist with supervision of the study and with data analysis and report writing. Dr. John Williamson has assisted with statistical design, and will continue to provide ongoing statistical guidance to the study. Dr. Meghna Desai will assist with supervision of the study and with data analysis and report writing.

# Abbreviations

AQ Amodiaquine

AQ/AS3 Amodiaquine + 3 days (doses) of artesunate

AS Artesunate

CDC Centers for Disease Control and Prevention

CE Cost effectiveness

CQ Chloroquine

CTX Cotrimoxazole

DALY Disability adjusted life year

DPT Diphtheria, pertussis, tetanus vaccination

DPT-HepB/Hib Diphtheria, pertussis, tetanus, hepatitis B, *Haemophilus influenzae* type B

vaccination

DSS Demographic surveillance system

EIR Entomological inoculation rate

EPI Expanded Program on Immunization

GAVI Global Alliance for Vaccines and Immunization

G6PD Glucose-6 phosphate dehydrogenase

HCW Health care worker

Hb Hemoglobin

HCS Hemoglobin color scale

Hib *Haemophilus Influenzae* type b

IEC Information, education, communication

IPD Inpatient department

IPTi Intermittent Preventive Treatment for Infants

# ITN Insecticide treated net

KEMRI Kenya Medical Research Institute

Lapdap Chlorproguanil-dapsone

MIC Minimum inhibitory concentration

# MOH Ministry of Health

MUAC Mid upper arm circumference

OPD Outpatient department

OPV Oral polio vaccine

PRP Polyribosylribitol phosphate (capsule of *H. influenzae*)

RCT Randomized controlled trial

RBM Roll Back Malaria

RDT Rapid diagnostic test

# SP Sulfadoxine-pyrimethamine

SP/AS3 Sulfadoxine pyrimethamine + three doses of artesunate

STGG Skim milk, glycerol and glucose

VR Village reporter

WHO World Health Organization

# Executive Summary

# Approximately three quarters of preschool children in eastern Africa suffer from anemia, defined as a hemoglobin (Hb) concentration below 11 g/dL [1]. For children < 5 years of age, the overall incidence of severe malarial anemia (Hb < 5 g/dl) is estimated at 15-60 cases per 1,000 children per year [2]. Other studies have confirmed that the burden of malaria-related anemia falls primarily on infants and young children [3, 4]. Recently, Schellenberg and colleagues, working in an area of Tanzania with a low to moderate level of *Plasmodium falciparum* transmission and a low level of sulfadoxine-pyrimethamine (SP) resistance, demonstrated that by linking intermittent prophylaxis to routine immunization visits through the national Expanded Program on Immunization (EPI), SP could be administered to children at 2,3, and 9 months of age, resulting in a 59% reduction in rates of clinical malaria and a 50% reduction in the rate of severe anemia (Hb<8 g/dl) compared to those receiving placebo[5]. We propose to conduct randomized double blind placebo-controlled trial to estimate the efficacy of Intermittent Preventive Treatment for Infants (IPTi) with SP + three doses of artesunate (AS) (SP/AS3) given in combination with iron supplementation from 2-6 months of age at routine EPI visits on the prevention of clinical malaria, moderate anemia, and severe anemia in the first 12 months of life in an area with intense malaria transmission and near universal ownership of insecticide treated nets (ITNs). The primary objective is to compare the efficacy of iron supplementation and IPTi with one of 3 antimalarial regimens (SP/AS3, chlorproguanil-dapsone (Lapdap), or AQ/AS3) given at routine EPI visits with iron supplementation alone (+ placebo) on the prevention of clinical malaria in the first year of life. Specific secondary objectives are: 1) Compare the efficacy of iron supplementation plus IPTi with one of 3 antimalarial regimens (SP/AS3, Lapdap [chlorproguanil-dapsone], or AQ/AS3) given at routine EPI visits with iron supplementation alone (+ placebo) on the prevention of moderate and severe anemia in the first year of life; 2) Assess the impact of IPTi with the aforementioned regimens on serologic responses to EPI vaccines (Polio, Diphtheria, Tetanus, Pertussus, Hepatitis B, *Hemophilus Influenzae* type B, and Measles; 3) Assess the impact of IPTi with the aforementioned regimens (particularly SP/AS3) on the nasal carriage rates of *Haemophilus influenza* type b; and 4) Compare the efficacy of iron supplementation and IPTi with one of 3 antimalarial regimens (SP/AS3, Lapdap [chlorproguanil-dapsone], or AQ/AS3) given at routine EPI visits with iron supplementation alone (+ placebo) on the prevention of all-cause hospitalization in the first year of life. This trial will generate important public health information on the efficacy of IPTi in preventing anemia and clinical malaria among infants in an area with intense malaria transmission and ongoing prevention efforts through the use of insecticide treated nets. This trial will contribute towards understanding IPTi’s mechanism of action (i.e. through intermittent clearance of parasites vs. a chemoprophylactic effect afforded through the use of an antimalarial with a long half-life). The information gained will be useful to determine the safety of IPTi, and to decide what sort of antimalarials are appropriate for IPTi, and ultimately will help to direct child survival and malaria control policy in African countries. If alternative drug regimes to SP prove effective, that information will be valuable to policymakers as levels of *P. falciparum* resistance to SP rise with increased usage in east Africa.

# Introduction and Background

## The burden of malaria in children

Malaria is a preventable and treatable disease that continues to cause between 700,000 and 2.7 million deaths each year, 75% of which occur among African children < 5 years of age [6]. Malaria and anemia are two of the leading causes of pediatric hospital admissions and mortality in many African countries [7]. Acute febrile illnesses are responsible for 400 to 900 million hospitalizations per year among African children < 5 years of age living in malaria-endemic areas [6], the majority of which are likely due to malaria.

## The burden of anemia in children

Approximately three quarters of preschool children in eastern Africa suffer from anemia, defined as a hemoglobin (Hb) concentration below 11 g/dL [1]. Anemia in early childhood leads to impaired physical growth and mental development, decreased physical fitness and resistance to infections [8-12], increased risk of subsequent obstetric complications in girls[13] and, when blood transfusions are needed, increased risk of HIV infection and other blood-borne pathogens [14].

# Iron deficiency and malaria as risk factors for anemia

Iron deficiency and malaria have been regarded as the predominant causes of anemia among African children. Their relative contribution depends on age, season and geographical area, reflecting local and time-dependent patterns in the availability of iron-containing foods and malaria transmission. Children are at highest risk for either iron deficiency anemia or malarial anemia between 6 and 36 months of age.

In preschool children, iron deficiency predominantly results from a poor dietary intake of iron coupled with rapid body growth. Although hookworm and schistosomiasis may be contributory factors in children in this age range, during infancy the prevalence and intensity of these infections is low in western Kenya: 4.0% for hookworm, and 0% for *Schistosoma mansoni* in children aged <12 months (Dr. Kim Lindblade, CDC/KEMRI, personal communication).

For children < 5 years of age, the overall incidence of severe malarial anemia (Hb < 5 g/dl) is estimated at 15-60 cases per 1,000 children per year [2]. In addition, malaria-associated severe anemia is an important cause of hospital admissions in malaria-endemic areas. In one hospital in Malawi, nearly 10% of admissions were for malaria-related severe anemia; the peak prevalence occurred among infants aged 6-11 months (15%) [7]. These rates were lower in children admitted to a hospital in an area not holoendemic for malaria [7]. Other studies have confirmed that the burden of malaria-related anemia falls primarily on infants and young children [3, 4]. Infants enrolled in a cohort study in a holendemic area in western Kenya had a hemoglobin nadir at 9-12 months of age which was 3.0 g/dL below the mean hemoglobin for age among children in non malarious areas [4]. In that same study, nearly one-third of infants 7-12 months of age had moderate or severe anemia (hemoglobin <8 g/dL), and more than three- quarters had some degree of anemia (hemoglobin < 11 g/dL) [4]. Further evidence from this same cohort suggests that infants and young children have a significant risk of anemia even with low density parasitemia, which may not be associated with fever or clinical illness [15]. It is increasingly recognized that in areas with intense malaria transmission the slow insidious process of repeated, often ‘asymptomatic’ (not associated with fever) malaria infections, leads to chronic anemia and is a major cause of severe anemia and malaria-associated death [16].

Together, these data suggest that malaria related anemia is an enormous public health problem in sub-Saharan Africa, that infants bear the greatest burden of this anemia, and that because the anemia is often associated with asymptomatic malaria infection, malaria prevention rather than treatment strategies are essential for combating the problem.

## Possible interventions to combat malaria and anemia in infancy

*Treatment*

The cornerstone of most malaria control programs is the case management of clinical or microscopy-proven malaria. However, data on the efficacy of case management in reducing mortality have been conflicting [17-19].

An alternative strategy to prevent malaria-related morbidity is to treat anemic children for malaria rather than just treating those with febrile illnesses. A recently completed randomized controlled trial (RCT) in Kisumu, western Kenya compared intermittent SP (monthly for 3 months) with or without supervised daily iron therapy (also for 3 months) to placebo for the treatment of mild anemia (7 < Hb < 10.9) in children <3 years of age. The results showed that supervised daily iron was more effective than intermittent SP for the treatment of mild anemia, and that the combination of the two interventions was slightly more effective than iron supplementation alone (M. Desai, Centers for Disease Control and Prevention, personal communication). A second phase of this study (children with 5 < Hb < 10.9) showed that supervised daily iron was superior to supervised twice-weekly iron for the treatment of mild anemia (M. Desai, Centers for Disease Control and Prevention, personal communication).

###### Prevention

Although treatment of anemic children for malaria could have great public health benefits in sub-Saharan Africa, there are enormous operational difficulties in screening children for anemia, as it is not a widely adopted practice. These difficulties, coupled with the near universality of anemia in infants and young children living in malaria-endemic areas of sub-Saharan Africa suggest the need for a preventive rather than a curative or case management approach.

Recognizing this need, trials of malaria chemoprophylaxis have been undertaken in African countries among school-age children. However the population that is often most at risk, children under 5 years of age and especially infants, is not readily accessible through schools or most community programs. At least one operational research project that relied on community health workers to distribute antimalarials was associated with decreased childhood mortality [20]. Unfortunately, community-based chemoprophylaxis programs have been difficult to implement on a wide-scale basis because of the logistic complexity and cost of most regimens. In addition, there is evidence that infants who receive chemoprophylaxis for malaria have a rebound increase in both severe anemia and clinical malaria morbidity once the intervention is stopped [21, 22]. Moreover, increasing concern about rising antimalarial resistance has made routine chemoprophylaxis less appealing. Currently, Kenya does not have a national policy of chemoprophylaxis for children or infants.

Recently, Schellenberg and colleagues, working in an area of Tanzania with a low to moderate level of  *Plasmodium falciparum* transmission and a low level of sulfadoxine-pyrimethamine (SP) resistance, demonstrated that by linking intermittent prophylaxis to routine immunization visits through the national Expanded Program on Immunization (EPI), SP could be administered under direct observation to children at 2,3, and 9 months of age. Two-month supplies of ferrous sulfate were provided to caretakers at the 2-month EPI visit and the 4-month weighing visit (total 4 months of daily iron supplementation) as a daily supplement to be administered in the home. Children receiving intermittent prophylaxis with SP had a 59% reduction in rates of clinical malaria and a 50% reduction in the rate of severe anemia (Hb<8 g/dl) compared to those receiving placebo.[5] In contrast to the increase in clinical malaria that has been observed among children following discontinuation of chemoprophylaxis [21, 22], no rebound effect was seen after discontinuation of SP in rates of clinical malaria or severe anemia. Thus, study investigators were able to demonstrate a clinically and statistically significant decrease in malaria-related morbidity by using an existing public health intervention that serves as a routine contact point for health-workers and children world-wide.

Although the results of this study were impressive, there are several features about its setting in Ifakara, Tanzania, that limit its generalizability to the rest of sub-Saharan Africa. First, the rate of parasitemia among the control group was very low, suggesting that transmission rates during the study period were low. Second, resistance to SP is low in Ifakara. In some other areas of east Africa, rates of resistance of *P. falciparum* to SP are rising or already high. Because many of the drugs that might some day replace SP for both treatment and prevention strategies are likely to have a shorter half-life than SP, it is important to understand whether the chemoprophylactic effect afforded by a long half-life drug such as SP is essential to the success of the intervention. Third, data on the usage of insecticide treated nets (ITNs) were limited in the Tanzania study. It is unclear what the additional benefit of intermittent preventive treatment might be in area with good coverage of ITNs.

Therefore, this innovative, effective, and potentially sustainable approach to anemia reduction (intermittent preventive treatment in infants, or IPTi) needs to be tested in an area with greater malaria transmission intensity, where SP use is already commonplace, and where ITN coverage is high and well measured. In addition, the efficacy of alternative antimalarial drugs in areas with high or increasing SP resistance should be measured.

## Potential alternative drug regimens to SP for IPTi

### Chlorproguanil-dapsone (Lapdap)

Chlorproguanil (Lapudrine*) in combination with dapsone (in development as a fixed combination: Lapdap) has been shown to be efficacious in treating *P. falciparum* malaria in a small study in Kenya (100% cure on day 7) [23], but less efficacious in the treatment of acute uncomplicated falciparum malaria in Thailand where resistance to SP is more common (14% cure rate) [24]. Another study in Kenya found that although clearance of parasitemia was good following both 1- and 3-course chlorproguanil-dapsone treatments (93.4% and 98.0%), re-infection rates were just as rapid as those seen in community surveillance [25]. A trial in pregnant women in Kenya comparing a single treatment course with SP, CQ, or chlorproguanil (1.2 mg/kg) and dapsone (2.4 mg/kg) given as a single dose found that chlorproguanil-dapsone was as effective as SP in clearing initial parasitemia by day 7, but less effective in maintaining parasite clearance by day 28 (67% parasitemic for chlorproguanil-dapsone vs. 19% for SP)[26]. A rise in Hb concentrations was observed in all 3 groups, but was sustained until day 42 only among those women who remained free of malaria parasites. There is evidence that the shorter half-life of chlorproguanil-dapsone may exert less selective pressure for drug resistance than longer-acting drugs like SP [27], and that although it may select for dihydrofolate reductase (DHFR) mutations, it does not select for dihydropteroate synthetase (DHPS) mutations [28]. Therefore, Lapdap is less likely to drive the evolution of highly resistant *P. falciparum* as quickly as other drugs such as SP.

### Amodiaquine

Amodiaquine (AQ) is a 4-aminoquinoline related to CQ. In persons taking AQ as weekly chemoprophylaxis, there have been reports of agranulocytosis and granulocytopenia [29, 30], hepatitis [31, 32], and increases in serum aspartate aminotransferase levels [33]. However, a recent systematic review of studies of AQ for the treatment of malaria found no increase in adverse events when compared with CQ or SP, and found that all adverse events were minor or moderate, and not life threatening [34]. A recently completed 3-country trial in Africa found no cases of clinical hepatitis among children >10 years receiving either AQ or AQ + artesunate, and no other amodiaquine-related serious adverse events, but did find that 6% of children developed neutropenia (neutrophil count <1000/μl) and that 60% of children experienced a decline in serial neutrophil counts [35]. In a smaller study involving younger (6-59 month) Tanzanian children, no serious adverse effects of AQ were noted, and no increase in neutropenia was noted when compared with children receiving SP [36].

In some settings with CQ resistance, AQ has been found to be more efficacious than CQ for treatment of malaria [37, 38]. A recently completed trial of AQ given as IPT (not-linked to EPI visits) found that the protective efficacy of AQ + iron supplementation in preventing malaria fevers and anemia was 64.7% and 67.0% respectively [39].

### Amodiaquine or SP in combination with artesunate

A recent multicenter African trial in children found improved efficacy in treating falciparum malaria in two of three countries among those treated with AQ + artesunate when compared with AQ alone [35]. AQ and artemisinins appear to have a synergistic antimalarial effect when examined in vitro [40].Artesunate given in combination with SP has similarly been shown to be effective and safe for the treatment of acute *P. falciparum* infections [41-43]. In addition to increasing the efficacy of SP and AQ, AS has been demonstrated to reduce gametocyte carriage, and substantially reduce the increase in gametocyte carriage often seen in persons treated with SP alone. [41-43].

### Pharmacokinetic differences among alternative regimens

The half-lives of the potential drugs for IPTi are markedly different. For SP, the range for the half-life of sulfadoxine is 100-231 hours (mean 169 hours), and of pyrimethamine is 54-148 hours (mean 111 hours) [44]. For Lapdap, the half-lives of the two components are 12.6 hours (SD 6.3) for chlorproguanil and 24.5 hours (SD 6 hours) for dapsone [45] For AQ, the half life of the parent compound is short (<10 hours), but the half life of its active metabolite, monodestethyl-amodiaquine, is similar to that of chloroquine [40].

Adding AS to either SP or AQ, while likely to improve efficacy in the clearance of parasitemia, will not extend the half-life of the combination beyond that of the primary drug, as the half-life of AS is very short (estimated at 4-11 hours) [46].

By comparing the efficacy of these drug regimens with components that have markedly different half lives, it may be possible to understand the mechanism of anemia prevention shown in the Tanzania study; that is, how much was due to the intermittent clearance of parasites, and how much to the prophylactic effect from the very long half-life of SP. If the effect found in Tanzanian infants was due to the intermittent clearance of parasites by the antimalarial, then one could hypothesize that a drug (or drug combination) with a shorter half-life would be just as efficacious in preventing anemia as SP, given comparable levels of drug resistance. If, however, the effect seen in the Tanzania study was due to a prophylactic effect of SP (given its long half-life), then the shorter acting drug under consideration (Lapdap) for this study may prove less efficacious for the prevention of anemia. It is also possible that the benefits observed in the Tanzania study will not be found in this setting with higher malaria transmission and greater coverage of ITNs. Because the benefits of this novel intervention have not yet been convincingly demonstrated (only one trial completed to date), it is important that a placebo arm be included in the study. All children in the study should benefit from iron sulfate administration in reducing anemia, regardless of the group to which they have been assigned.

### Availability and cost of alternative regimens

Sulfadoxine-pyrimethamine, amodiaquine, and artesunate are available and licensed for use in Kenya. Only SP is widely available in government health facilities. The cost of SP is low – approximately $ 0.08 per adult treatment course. The cost of a pediatric AS treatment course is difficult to determine, but is likely less than $ 2.00 (a full adult treatment course would be about $2). The cost of AQ for an adult treatment dose is approximately $0.15. The cost of Lapdap is less than $0.50. Lapdap is now commercially available in Kenya,.

If any or all of these drugs prove efficacious, wide scale programmatic implementation should be feasible.

Impact of routine antimalarial usage on carriage of *Haemophilus influenzae* type b (Hib)

While the antibody concentration for long-term protection against invasive Hib disease is 1 μl/ml, the IgG concentration needed for protection against Hib carriage is 5 μl/ml. This is because the mucosal antibodies needed to block carriage are from transudation from serum. Because of this difference in protective thresholds (invasive disease vs. carriage), it is possible that malaria may decrease the immune response to Hib conjugate vaccine to a level that will allow Hib carriage despite protection against invasive disease. If this is the case, the herd immunity achieved by mass vaccination with a conjugate vaccine may not be achieved because there will still be carriage among children in malarious areas. Unvaccinated children or poor responders will still be subject to acquiring invasive Hib disease. For this reason, it is important to measure the effect of malaria and PIPIT on Hib carriage among children.

## Impact of routine antimalarial usage on response routine vaccinations

In order for this intervention to have a programmatic future, it must be convincingly demonstrated that IPTi does not have a negative effect on the development of antibodies to routinely administered vaccinations. This will be achieved by comparing the proportion of infants in the intervention (IPTi) and placebo groups that attain post-vaccination geometric mean antibody titers (GMTs) above a pre-defined protective level for each EPI vaccine.

# Justification

This trial will generate important public health information on the efficacy of IPTi in preventing anemia and clinical malaria among infants in an area with intense malaria transmission and ongoing prevention efforts through the use of insecticide treated nets. This trial will contribute towards understanding the mechanism of action through which IPTi achieves reduction in anemia and clinical malaria (i.e. through intermittent clearance of parasites vs. a chemoprophylactic effect afforded through the use of an antimalarial with a long half-life). The information gained will be useful to decide what sort of antimalarials are appropriate for IPTi, and ultimately will help to direct child survival and malaria control policy in African countries. If alternative drug regimes to SP prove effective, that information will be valuable to policymakers as levels of *P. falciparum* resistance to SP rise with increased usage in east Africa.

Information from this study may prove useful to policymakers at WHO, UNICEF, and the Kenya MOH for evaluating the safety of IPTi, particularly with relation to response to EPI vaccines.

**Objectives**

## Primary objective

Compare the efficacy of iron supplementation and IPTi with one of 3 antimalarial regimens (SP/AS3, Lapdap [chlorproguanil-dapsone], or AQ/AS3) given at routine EPI visits with iron supplementation alone (+ placebo) on the prevention of clinical malaria in the first year of life.

## Secondary objectives

1. Compare the efficacy of iron supplementation plus IPTi with one of 3 antimalarial regimens (SP/AS3, Lapdap [chlorproguanil-dapsone], or AQ/AS3) given at routine EPI visits with iron supplementation alone (+ placebo) on the prevention of moderate and severe anemia in the first year of life.
2. Assess the impact of IPTi with the aforementioned regimens on serologic responses to EPI vaccines (Polio, Diphtheria, Tetanus, Pertussus, Hepatitis B, *Hemophilus Influenzae* type B, and Measles).
3. Assess the impact of IPTi with the aforementioned regimens (particularly SP/AS3) on the nasal carriage rates of *Haemophilus influenza* type b.
4. Compare the efficacy of iron supplementation and IPTi with one of 3 antimalarial regimens (SP/AS3, Lapdap [chlorproguanil-dapsone], or AQ/AS3) given at routine EPI visits with iron supplementation alone (+ placebo) on the prevention of all-cause hospitalization in the first year of life.

# Design and Methods

## Study Design

We propose to conduct a double blind, placebo controlled, randomized controlled efficacy trial (RCT) among children under longitudinal observation to examine the above-mentioned questions. Children will be recruited during the first EPI visit at 6 weeks of age and will be followed prospectively with collection of epidemiologic, clinical, and laboratory data. Routine EPI visits in Kenya occur at 6, 10, and 14 weeks, and again at 9 months of age. IPTi will be provided under supervision at 10 and 14 weeks and again at 9 months with one of 3 drug regimens or placebo. From ages 2-6 months, supplementation with ferrous sulfate will be given as part of the intervention package.

The 4 arms of the study are as follows

I Intermittent SP/AS3 treatment with daily iron supplementation

II Intermittent Lapdap treatment with daily iron supplementation

III Intermittent AQ/AS3 treatment with daily iron supplementation

IV Intermittent placebo treatment with daily iron supplementation

## Study site

The study will take place in Asembo (Rarieda Division), western Kenya; the total population is approximately 55,000 persons in 76 villages over 200 square kilometers. If there is difficulty in recruiting the full cohort from Asembo, one clinic from neighboring Gem (wagai division, total population approximately 56,000 persons in 105 villages over 172 square kilometers) may be added. This is an area of intense perennial transmission. Entomological inoculation rates in this part of western Kenya were once high, ranging from 237-299 infective bites per person per year.[47]. At present, the high rate of coverage with ITNs has reduced the EIR to approximately 10 infective bites per person per year. Although transmission is perennial, there are two rainy seasons and therefore some seasonal variation in transmission intensity.

In western Kenya, pediatric mortality rates continue to be very high; 32/1,000 live births for neonates, 176/1,000 live births for infants, and 257/1,000 live births for children < 5 years of age.[48] Based on the results of an ITN study in this area, it is estimated that approximately 20% of infant deaths are malaria-related (F. ter Kuile, Centers for Disease Control, and P. Phillips-Howard, personal communication).

ITN ownership and usage rates are high in the study area following several years of intense distribution as part of a research study. Nearly 100% of households have an ITN, and usage has been estimated at 70% (F. ter Kuile, Centers for Disease Control and Prevention and P. Phillips-Howard, personal communication).

EPI coverage in Nyanza province is moderate: 91-92% of children receive the first dose of diphtheria pertussis tetanus vaccination (DPT) and oral polio vaccination (OPV), 80-82% receive the second doses of those two vaccines, and 66% receive the third dose. Measles vaccination, given at 9 months of age, is received by 62% of children. [49] These data have recently been confirmed through a cross-sectional survey in the study area showing a rate of 79.4% for the second dose of DTP and OPV. The normal immunization schedule is BCG immunization soon after birth, DTP and OPV at 6, 10 and 14 weeks of age, and measles vaccination at 9 months. Recently (December, 2001), the Kenya MOH began administering a pentavalent vaccine (diphtheria, tetanus, pertussis, hepatitis B, and Hib) in place of DTP. All clinics in the study area now use this vaccine; the vaccination schedule has remained the same.

SP has been used extensively in trials in the study area since 1992; SP was adopted as first line therapy by the Kenya MOH in 1998. Recent data suggest that there is parasitologic treatment failure rate of approximately 28% at day 7 among children < 2 years of age treated with SP (Dr. Feiko ter Kuile, Centers for Disease Control, personal communication).

CDC has a longstanding collaboration with the Center for Vector Biology and Control Research, KEMRI in Kisumu, and there is an excellent research infrastructure already in place. There is full-time CDC staff employed at the KEMRI-CDC research station. In addition, there is a demographic surveillance system in place that may allow for the measurement of impact of a study intervention on mortality.

The area is served by 2 district hospitals (Bondo and Kisumu) between 10 and 60 km from the study area, and 1 mission hospital. There are 6 peripheral health units located in Asembo. These units are comprised of government, community, private, and mission facilities, and are normally staffed by an enrolled community health nurse. Only one of the peripheral health units currently has diagnostic facilities for malaria.

## Demographic Surveillance System

A demographic surveillance system (DSS) is a process that involves continuous monitoring of a geographically defined population to generate high quality, population-based, longitudinal health and demographic data. A well-developed DSS can provide two basic kinds of information

1) General demographic and health information. This would include such elements as population (age structure and density), pregnancies (fertility rate), births, in- and out-migrations, patterns of health care access and utilization, morbidity (including hospitalizations) and overall and cause-specific mortality.

2) Disease or intervention specific information. DSS also provides an invaluable platform on which to base a range of studies to examine specific disease issues, including descriptive epidemiology, diagnosis, treatment, prevention, behavior, and economics. These studies can take advantage of the sampling frame inherent in a DSS, whether at individual, household/compound or neighborhood level, in that a whole population sample removes many of the biases of other study designs. For example, high quality DSS data are an essential component to assess the impact on mortality of malaria control measures such as IPTi.

From 1996-2001, investigators working at the KEMRI-CDC research station have been conducting a form of demographic surveillance in selected populations. This surveillance activity has been conducted in the context of a large trial examining the immediate and long-term effect of ITNs on all-cause mortality in children. At present, approximately 55,000 persons are under surveillance in the Asembo area. Demographic surveillance has been conducted through biannual house-to-house censuses. Facility-based surveillance at two district hospitals and 6 outpatient clinics in Asembo have provided information on types of illnesses in children in this area that result in health-care seeking behavior and hospitalization. Systematic and continuous entomologic monitoring of anophelines has provided ongoing estimates of the entomologic inoculation rate (EIR); EIR data provide critical information on the intensity of malaria transmission. Ongoing assessment of EIR is necessary to understand if changes in malaria morbidity and mortality are due to an intervention or to natural variation in the intensity of transmission pressure. Complete GIS mapping of the study area has been conducted. Enrollment forms collect information on new residents.

In late 2001, all households and residents in the surveillance area were registered into an upgraded DSS system. Continuous DSS fieldwork is conducted on a 4-month cycle. This cycle begins the first workday of each January, May, and September. Each cycle begins with one week of training, followed by 11 weeks in the field during which every registered household is visited, followed by one week of vacation for the field teams, and finally 4 weeks to resolve discrepancies in the data, catch up with data management and report writing, and conduct special surveys.

During routine DSS interviews, an adult household member is asked about demographic, health and other events within the household. All information is recorded in a household record book and on individual event forms.

To increase accuracy and timeliness of information on births and deaths, every village has a village reporter (VR), a resident who volunteers to report information on every birth or death among households in his/her village.

As part of the DSS activities, there is monitoring of both outpatient (OPD) and inpatient (IPD) visits for children in Asembo. 7 outpatient health facilities, and 2 inpatient facilities have an assigned DSS staff member who interviews all patients and records information onto a standardized form. Children who attend outpatient clinics are linked to the DSS using their permanent IDs cataloged in registers at each health facility. We estimate that approximately 80% of children or their mothers can be linked to their records in the DSS.

## Study population

*Inclusion criteria: Infants attending any one of the 4 study clinics (Abidha, Ongielo, Lwak, and Saradidi or another comparable clinic in Asembo or Gem) for immunization at the 6-week visit will be offered enrollment in the study.*

1. Age 5 weeks to 16 weeks

2. Parent or guardian currently resident in Asembo (or Gem).

3. Parent or guardian has given permission for their child to participate

### Exclusion criteria

1. Known allergy to any of the study drugs

2. Current Cotrimoxazole prophylaxis

3. Concomitant disease requiring hospitalization or transfusion

4. Plans to be away from the study area for more than 6 months during the next year

## Sample Size

This study primarily aims to determine if routine intermittent treatment with antimalarials results in a reduction in childhood clinical malaria, by assessing time to first episode of clinical malaria.

We estimate that the median time to first episode of clinical malaria in the placebo group receiving iron is 18 months (68 weeks after the first intervention visit). The sample size required for 90% power to detect a protective efficacy of 40%, assuming α=0.017, is 250 per arm. Alpha has been adjusted to 0.017 in order to account for multiple comparisons (3 arms each compared to the placebo group, Bonferroni adjustment 0.05/3 tests=0.017). Therefore, the study would require a total sample of 1000 infants (250 in each of 4 groups). We expect that 11% of infants will have the outcome measure at baseline and thus will be excluded from the primary analysis. Including this decrease and adjusting for a 10% loss to follow up and 13% mortality, the study will require 1516 infants (379 in each arm).

## Sampling procedure

All infants attending one of the study clinics for 6-week vaccination visit (the first post-birth scheduled EPI visit in Kenya) will be screened for enrollment. The parents or guardians of infants meeting the study inclusion criteria and not meeting any of the exclusion criteria will be offered enrollment in the study.

## Assignment to interventions

All infants whose parents or guardians agree to participate in the study will be assigned to one of the 4 treatment groups through permuted block randomization[50]. The randomization code will be prepared before the trial starts.

These randomization procedures are expected to minimize the possible influence of geographical location and seasonal malaria variations.

# Clinical Procedures

## Enrollment

At enrollment, capillary blood samples will be taken using finger or heel prick and collected into capillary tubes for later determination of antibodies to polio, diphtheria, tetanus, hepatitis B, and Hib.

It will take approximately 15 minutes for mothers of enrolled infants to respond to the enrollment questionnaire, which will be administered face to face by a trained interviewer, and for the capillary blood sample to be obtained.

The mother of each enrolled child will be issued an identity card with a photo of the infant and mother that will be used to confirm identity of the child at both scheduled and unscheduled visits for the duration of the study.

## Study drugs

As this is an efficacy study, all doses of antimalarials (or placebo) will be supervised. All enrolled infants will receive Day 1 dose(s) of study drugs during the 10 and 14 week intervention visits and again at the 9 month intervention visit. On days 2 and 3, parents will either be asked to return to the clinic for study drug administration, or the dose will be administered in the home by a compliance monitor. In the case of infants whose guardians were asked to return to clinic to receive the 2^nd^ and 3^rd^ doses of medication and who do not appear by noon of the appointed day, a compliance monitor will visit the home to administer the dose of medication. All medications will be dispensed as crushed tablets, not as commercial suspensions, in order to ensure accurate dosing. Mixing with syrup will be done at the moment of delivery to make the medicines more palatable.

*Sulfadoxine-pyrimethamine (SP) and artesunate*

SP (DAFRA Pharma, Belgium) will be given by the study team as crushed tablets suspended in syrup. The dose-equivalent will be one ½-strength tablet (250 mg sulfadoxine, 12.5 mg pyrimethamine) for all infants given a single time on the first day of treatment. The artesunate dose will be one 25 mg tablet, once daily for 3 days, also given as a crushed tablet in syrup.

*Chlorproguanil-dapsone (Lapdap)*

Lapdap (Glaxo-Smithkline, United Kingdom) will also be given as crushed tablets suspended in syrup. One pediatric LapDap caplet (15 mg chlorproguanil and 18.75 mg of dapsone) will be administered once daily for 3 days.

*Amodiaquine + artesunate* (DAFRA Pharma, Belgium)

Amodiaquine will be given as crushed tablets suspended in syrup. The dose will be one 67.5 mg tablet daily for 3 days. Artesunate will be given as for the SP arm.

*Placebos* (DAFRA Pharma, Belgium)

To ensure masking, placebo (tablets of lactose and maize starch) and treatments will be indistinguishable in terms of color and appearance, and will be provided pre-crushed in identical tubes that will be grouped as 6 tubes for each child. In the case of SP/AS3, the first day will have two tubes in the pack containing study drug (one containing SP and one containing AS), and the remaining days will have one tube with placebo (for SP) and one tube with AS. In the case of AQ, the first, third, and fifth tubes in the pack will contain AQ and the all other tubes will contain AS. In the case of Lapdap, the first, third, and fifth tubes will be study drug, and the other tubes will contain placebo. All tubes will contain placebo for the placebo arm.

### Iron

Twelve mg/day of oral iron (roughly 2 mg/kg/day) will be given to all study children for 4 months (from recruitment at 10 weeks until 6 ½ months of age). Iron will be given as drops of a concentrated solution of ferrous sulfate containing 25 mg/ml. A one month supply will be given at the 10-week visit, an additional one month supply will be given at the 14-week visit, and a 2 month supply will be given at the 18 week visit. Iron will be dispensed in bottles with a dropper device in the neck to facilitate accurate dispensing and with a childproof closure. Compliance with iron therapy will be assessed by using a measuring stand to determine quantity used at the 14 week, 18 week, and 6 ½ month study follow-up visits (D. Schellenberg, personal communication).

### Drug quality

We will obtain the manufacturer’s certificate of analysis for one lot of each study drug.

## Children who are febrile and parasitemic at enrollment

Infants who have documented fever (> 37.5 C by axillary measurement) or history of recent fever (in the previous 48 hours) will have a rapid diagnostic test (RDT) for malaria performed. If the RDT is positive, the infant will be given antimalarial treatment in accordance with national policy. A blood film will also be performed; febrile infants who had a negative RDT, but on later blood film examination have parasitemia will be given antimalarial treatment in accordance with national policy. These results and the antimalarial therapy will be delivered to the infant’s home only if the results of the blood film are positive while the RDT was negative. These patients will be included in the study.

## Intervention clinic visits

Enrolled infants returning to clinic at 10 weeks will be given study drugs and will be measured for recumbent length, weight, and mid-arm upper arm circumference. Capillary blood samples will be taken using finger or heel prick for later G6PD enzyme level testing. Blood will be collected on filter paper at the 10 week visit for later testing for genetic markers for drug resistance. Infants returning to the clinic at14 weeks will be given the 2^nd^ dose of the study drugs. At the 9-month visit, infants will be given the final dose of the study drugs and a capillary blood sample will be taken for later measles serology testing.

A questionnaire soliciting information about overall health of the baby, febrile episodes, other intercurrent illnesses, use of other medications, ITN usage, and compliance with iron will be administered at each clinic visit. The form will be based on the OPD surveillance form used in the DSS, but will solicit more detailed information. Infants whose caretakers state that their child has been given CTX in the past 2 weeks will either a.) not be given the dose of intervention drug until 2 weeks after CTX was given, or b.) be withdrawn from the study (if CTX is given as a daily dose).

If the infant is reported or observed to be ill, a full clinical exam will be performed. Infants at the 10- and 14-week visits who have documented fever (> 37.5 C by axillary measurement) or history of recent fever (previous 48 hours) will have an RDT for malaria performed. If the RDT is positive, in order to maintain blinding of the study groups, there will be a treatment regimen paired to each of the study arms. In the case of the three arms that include an antimalarial (SP/AS, AQ/AS and Lapdap), the “treatment” drug will in fact be a placebo. Each of the three non-placebo arms of the study involves the administration of a drug that is adequate and appropriate treatment for malaria. In the case of children enrolled in the placebo arm of the trial, the treatment drug will be a 7-day course of quinine, which is one of the MOH recommended drugs for treatment of malaria. The guardians of all febrile children will also be provided with a 3-day supply of acetaminophen (paracetomol). A blood film will also be performed on all infants who have an RDT performed. Any infant who was febrile, but had a negative RDT and is found to have parasitemia by blood film will be given antimalarial treatment in accordance with the paired scheme above. Infants who appear pale will have their hemoglobin measured. Infants with Hb<8 g/dl at the 9 month visit (as infants at the 10 and 14-week visits will already be taking iron) will receive iron per the national policy.

At each of the study clinics, compliance monitors will work in conjunction with a village recorder to remind enrolled families (with an oral and written message delivered to the home) about upcoming study clinic visits. A compliance monitor will visit the houses of families who fail to appear within one week of an intervention visit (10 weeks, 14 weeks, and 9 months).

## Follow-up clinic visits (non study drug administration visits)

Parents will be asked to bring enrolled infants back to the clinic for follow up visits at 18 weeks, and at 12, 18, and 24 months. A compliance monitor will visit the houses of families who fail to appear within 2 weeks of a follow-up visit. To facilitate the work of compliance monitors, a photograph of each mother and child will be made at enrolment, and that photograph kept with a map locating the place of residence. These documents will be returned to the mother at the conclusion of the study or destroyed.

At the 18-week visit, blood (from the same finger prick) will be obtained and frozen at –70°C for later determination of post-vaccination serologic testing for antibodies to polio, diphtheria, tetanus, hepatitis B, and Hib. At the 12-month visit, blood will be obtained for determination of sickle cell trait and frozen for later determination of measles antibody titer. Blood will also be collected on filter paper for later testing of genetic markers for drug resistance.

A questionnaire (identical to that administered during the intervention visits) soliciting information about overall health of the baby, febrile episodes, other intercurrent illnesses, use of other medications, ITN usage, and compliance with iron will be administered.

The algorithm for antimalarial treatment of children who are febrile at the 18 week, and at 12, 18, and 24 month visits will be the same as for the intervention visits, with the exception that all infants who need treatment will be provided quinine (18-week visit) or Coartem (12, 18, and 24-month visit if the child weighs at least 5 kg), as no children will be receiving study drugs at these visits. Children weighing less than 5 kg requiring antimalarial treatment will receive quinine. Children with severe malaria will treated with quinine, regardless of age. Infants with Hb<8 g/dl at the 12, 18, or 24-month visits will be treated with iron per the national policy.

#### Note: Coartem (Artemether-Lumefantrine [AL])

Coartem is being provided as a treatment for clinical malaria in children who have made their 12 month visit. It is not an experimental drug, and is not being evaluated in this study. It does not constitute part of the experimental procedures of the study.

Coartem is a highly efficacious antimalarial drug, and is a combination of Lumefantrine, is a novel aryl amino alcohol antimalarial and artemether, one of the artemesinin drugs (that includes artesunate). Coartem is more costly than SP - a subsidized adult course costs approximately 200 KSh, or $2.40, while an unsubsidized adult course of Coartem cost 400 Ksh, or 5 USD. Coartem is a registered product in Kenya. Coartem is a first-line drug for the treatment of malaria in Kenya. Coartem is the primary antimalarial recommended by the World Health Organization for treatment of uncomplicated malaria in countries with antimalarial drug resistance.

Existing data suggest that this ACT is safe and that the efficacy of Coartem is high (Hassan Alin, Bjorkman et al. 1999; van Vugt, Ezzet et al. 1999; Ezzet, van Vugt et al. 2000). There have been no significant side effects reported from the use of artemether in humans. Most studies report adverse events as mild or moderate in severity (including dizziness, headache, anorexia, nausea and abdominal pain), noting that these events may also be attributable to malaria (Hatz, Abdulla et al. 1998; van Vugt, Ezzet et al. 1999; van Vugt, Looareesuwan et al. 2000)

Home visit

At 6 months of age, a home visit will be made to check on compliance with iron administration. At this visit, the compliance monitor will ask questions about iron administration, and will measure the amount of iron remaining in the bottles.

## Unscheduled visits

Parents of enrolled infants will be instructed to take them to one of the study health facilities for evaluation and treatment of all illnesses. Study infants who make such unscheduled visits for febrile illness during the study period will have a malaria RDT performed. Those with a positive RDT will be treated with a treatment course of quinine if they present before their 12-month visit. Treatment with quinine (instead of SP or Coartem) will avoid the possibility of giving doses of SP or artemisinin that are too closely spaced in time while the child is receiving study drugs. Those with a positive RDT at or after their 12-month visit will receive Coartem, unless they weigh less than 5 kg, in which case, they will receive quinine. All children who are febrile will also have a malaria blood film performed at the same time. All children who have a blood sample taken for malaria diagnosis or have signs or anemia will also have a hemoglobin measurement performed. If any infant who had documented fever but was negative for malaria by RDT is later found to have malaria by microscopy, the results and a treatment course of quinine (or Coartem) will be delivered to the infant's home. Blood from the same finger-stick blood sample will be dried on to filter paper for analysis on molecular markers of antimalarial drug resistance.

During weekends and evenings, a nurse will be available to assist IPTi participants who are severely ill. The nurse will be stationed at Lwak hospital, our most central study clinic. In cases where the infant must be hospitalized, the nurse will provide stabilizing treatment and the IPTi Field Supervisor, Coordinator, or designee will arrange transport to Bondo or Provincial hospital.

Any child that has an allergic reaction (severe cutaneous reaction, anaphylaxis, or other severe reaction) associated with any study drug will be withdrawn from receiving further study drug. The study code will be broken for that child, so that a determination of possible drug allergy can be determined. Parents will be informed about the possible allergy, and that information will be recorded on the child health card. If future treatment for malaria is needed, parents will be informed that it should be carried out with a drug other than one in the class to which the child had a reaction. The child will continue to be followed as per protocol with the exception that the child will not receive any additional doses of study medication. The parent or guardian maintains the right to completely withdraw the child from the trial at any time (meaning that no further follow-up would occur).

# Laboratory procedures

Thick blood smears made from capillary blood will be stained with 2-3% Giemsa stain (pH 7.2) for 45 minutes and examined for parasites. Parasites and leukocytes will be counted in the same fields until 500 leukocytes are counted. Parasite densities will be estimated using an assumed leukocyte count of 8,000 leukocytes per μl of blood. The limits of detection are estimated at <10 parasites/μl of blood.

Dried filter papers will be sent to the London School of Tropical Medicine and Hygiene for analysis of molecular markers of resistance. Although capacity exists in Kenya to conduct this analysis, this central reference lab will be used by all study sites for this analysis, to ensure quality assurance and comparability of results across IPTi sites. To further develop molecular analysis capacity at KEMRI/CDC, one laboratory technician from KEMRI/CDC will travel to London to analyze the samples in the central laboratory.

Capillary blood will be collected in a microcuvette and examined in a Hemocue machine to determine the Hb level. Capillary blood will also be collected into a Microtainer tube for sickle cell testing, and EPI vaccine antibody testing. Analysis of serologic response to EPI vaccines will be performed by the Pasteur Institute in France or another comparable laboratory selected by WHO.

Sickle cell testing will be done in Kenya using a standard Hb electrophoresis technique. Testing for glucose-6-phosphate dehydrogenase genotype will be performed by polymerase chain reaction (PCR). For the primary analysis of the immune response to Hib vaccine, concentrations of anti-PRP antibody will be compared in the SP and placebo groups at 18 weeks. As part of the secondary analysis, the immune response to vaccine among children in the placebo group who have symptomatic malaria, asymptomatic malaria, and no malaria will be evaluated.

Immediately after nasopharyngeal swabs are obtained they will be placed in a culture media, consisting of skim milk, glycerol and glucose (STGG). The swab tip will be cut off into the STGG media and the media will be refrigerated until ready for transport. Cryotubes with the swabs in the STGG media will be frozen at -70^o^C at the laboratory in Kisian. When ready for processing, 100 μl of the STGG-bacteria mix will be thawed and spread on a Hib halo plate, allowing the fluid to be absorbed before inverting the plate. The Hib halo agar is made as a chocolate agar medium with the addition of bacitracin and 1 ml of anti-Hib serum. In order to transfer technology and build KEMRI/CDC laboratory capacity, we propose to sponsor one or two KEMRI/CDC laboratory staff to come Atlanta to acquire the technical skills to conduct this testing. This training will then be followed up by visits to the KEMRI/CDC laboratory from senior CDC/Atlanta staff for quality assurance.

#

## Quality control

A randomly selected 10% sample of malaria blood films will be re-read by an independent microscopist for quality control purposes.

The Hemocue machine will be compared against a known standard after every 20 samples. The Hemocue machine will be calibrated weekly or whenever it reads a difference of +/0.3 g/dl from the known standard.

# Definitions

# A clinical malaria episode in an infant will be defined as an axillary temperature of at least 37.5°C in combination with asexual *P. falciparum* parasitemia of any density . Mild-to-moderate anemia will be defined as Hb <11.0 and ≥8. Severe anemia will be defined as Hb <8 and ≥5. Critical anemia will be defined as Hb <5.

**Data Management**

Data cleaning and storage:

Quantitative data will be entered using scan-ready forms and an optical scanner. Data will be entered and edited in Kenya. Images of scan forms will be kept on a secure server with access limited to study personnel. Data analysis will be undertaken collaboratively in Kenya and at CDC in Atlanta. Access to study computers will be restricted to study personnel, and paper records will be secured in locked cabinets in Kenya. Paper records will not be transported to Atlanta. Electronic files will be transferred to Atlanta either as password-protected e-mail attachments or as password protected files on zip disks or CDs.

Sample storage

Specimens will be stored in locked freezers or cabinets with identifiers at KEMRI and at CDC, to which only study personnel will have access.

In accordance with WHO advice, testing for serological responses to EPI antigens will be performed at a single commercial reference laboratory. The commercial laboratory will only have coded specimens, and will not be engaged in the research. Any remaining sera will be shipped back to Kenya.

Statistical analysis:

SAS software (SAS Institute, Cary, NC), and STATA software (STATA corporation, College Station, TX) will be used for quantitative data analysis. The primary analysis will be based on intention to treat. The analysis will be time to first clinical malaria comparing each of the study drug arms to the placebo arm. Mild, moderate, and severe anemia will also be compared between each study drug arm and the placebo arm.

**Time Frame**

Preparatory time

Preparatory time of approximately one month will be required to train facility-based study staff in clinical procedures and questionnaire administration, and to train field-based staff in patient tracking, cross-sectional surveillance, and the administration of household-based follow-up questionnaires.

Data collection

We estimate that there will be on average 139 visits/month at the 4 clinics at which enrollment will take place (Lwak, On’gielo, Abidha, and Saradidi or another comparable clinic in Asembo or Gem), based on 2001 utilization rates (Kim Lindblade, personal communication). If 80% of women offered accept enrollment into the study, then it should take approximately 14 months to enroll 1516 infants into the study. Since follow up will be for 2 years after the last child is enrolled, we estimate that data collection should take approximately 3.5 years. To allow for the possibility of slower than anticipated enrollment, we are budgeting 3 ½ years for complete data collection.

Preliminary data analysis and report preparation should require 6 months. Final manuscript preparation should require an additional 6 months.

Study results will be presented to local health authorities and to the participating communities at the conclusion of the study and analysis.

Good Clinical Practice

This conduct of this trial shall be guided by the principles of Good Clinical Practice (GCP).

##### Study Schedule

|  | 6  w | 10 w | 14 w | 18 w | 6 m | 9 m | 12 m | 18 m | 24 m |
| --- | --- | --- | --- | --- | --- | --- | --- | --- | --- |
| Enrollment | X |  |  |  |  |  |  |  |  |
| Clinic visit | X | X | X | X |  | X | X | X | X |
| Sickle Cell screening |  |  |  |  |  |  | X |  |  |
| Blood draw for G6PD testing |  | X |  |  |  |  |  |  |  |
| Study drug given |  | X | X |  |  | X |  |  |  |
| Blood film |  |  |  |  |  |  | X | X | X |
| Hemoglobin |  |  |  |  |  |  | X | X | X |
| Anthropomorphic measurements |  | X |  |  |  |  | X | X | X |
| Vaccinations | X | X | X |  |  | X |  |  |  |
| Blood for EPI antibody testing) | X |  |  | X |  | X | X |  |  |
| NP swab Hib |  |  |  |  |  | X |  |  |  |
| Iron Compliance |  |  |  |  | X |  |  |  |  |
| Filter paper |  | X |  |  |  |  | X |  |  |

#### Ethical Considerations

Informed consent procedures

All mother’s/guardians of children who are eligible for the study will be informed about the reason for the study. Each activity and requirement of the study will be explained to them. Informed consent forms will be translated into the local language (luo), and backtranslated to check for accuracy. Informed parental permission forms will be signed or thumb-printed (for women who cannot sign their names) for documentation of willingness to participate. If the woman is illiterate, a witness (not connected with the research study) will sign the permission form, attesting to the fact that the permission was accurately read or interpreted, and that participation and thumb-printing were given willingly without coercion. All women who are approached will be informed that their children’s entry into the study is voluntary and that they may withdraw from the study at any time.

Confidentiality

In order to protect the privacy of patients, only study personnel will have access to paper records, and those records will be kept in a locked file in Kisian. Computer records will be password protected, and only authorized study personnel will have access to those files.

## Risk/benefit information

Benefits to participating infants include free health care for routine childhood illnesses (malaria, anemia, diarrhea, and acute upper and lower respiratory infections) for a period of 24 months starting from the first day of that child’s enrollment in the study. The IPTi study ID card will also serve as a “health passport” which allows the child to receive free treatment for these conditions in one of the study health facilities for this period. The child will be seen by a dedicated study clinician, which may result in shorter wait times for medical care. Inpatient hospital costs at Lwak and Abidha hospitals will also be covered for enrolled children. Any other medical costs (e.g. specialty care) are not covered by the project unless the illness is deemed to have been caused by one of the study medications. All children will receive free iron, which has demonstrated benefits in reducing anemia. Not all children will receive routine antimalarials (i.e. the IPTi intervention) because the benefit of IPTi is not known in this setting. Because enrolled children will visit the clinic 5 extra times during the study, they are likely to have malaria and severe anemia detected and treated more quickly than usual. In addition, enrolled children will be receiving RDT and blood film diagnosis for febrile illnesses, which may allow for more accurate diagnosis and appropriate treatment of these illnesses. Guardians will be informed if an enrolled child has sickle cell disease deficiency, which might help to prevent adverse outcomes.

During weekends and evenings, a nurse will be available to assist IPTi participants who are severely ill. The nurse will be stationed at Lwak hospital, our most central study clinic. In cases where the infant must be hospitalized, the nurse will provide stabilizing treatment and the IPTi Field Supervisor, Coordinator, or designee will arrange transport to Bondo or Provincial hospital. This may benefit very ill infants. Guardians of infants who are critically anemic (Hb < 5) will be provided transport to a hospital (Kisumu or Siaya) for evaluation and treatment.

So that no costs are incurred by participating in this study, parents or guardians will be given a standard reimbursement for transport costs from their homes to the clinic and back when they bring their children for scheduled study visits. Parents or guardians will also be reimbursed for travel when it is necessary to bring the child to one of the study clinics after vomiting a dose of the study medication administered at home by one of the study compliance monitors. Standard reimbursement will be the average cost of a 'Boda-Boda' bicycle ride to and from the clinic, which is between 50 and 100 Kenyan Shillings, depending on distance

There a small risk from study medicines. Some children do not like the taste of iron. Some children complain of nausea and do not feel like eating. In some children the teeth will become darker. In other children the stool will become darker or greener than normal. This is a normal reaction to treatment with iron. The teeth and stool color will become normal again after the iron treatment is completed.

When given weekly as prophylaxis, SP has been associated with extremely rare severe cutaneous reactions such as toxic epidermal necrolysis and Stevens-Johnson syndrome [51].

Amodiaquine has rarely been associated with hepatitis and agranulocytosis in adults receiving the drug as weekly chemoprophylaxis [29-32], but a recent systematic review of studies of AQ for the treatment of malaria found no increase in adverse events when compared with CQ or SP, and found that all adverse events were minor or moderate, and not life threatening [34]. In children given AQ as a treatment dose, the drug appears safe [35, 36, 39], although an incidental finding of a decrease in neutrophil counts has been reported, without any associated clinical significance [35].

There are no reports of severe adverse reactions among children in trials using Lapdap for treatment of *P. falciparum* malaria [23, 25]. Occasional reports have been received of adult travelers developing agranulocytosis following Maloprim (a combination of dapsone and pyrimethamine) chemoprophylaxis, although usually after twice-weekly dosing, which is no longer recommended [52]. However, a study of chemoprophylaxis of Tanzanian infants with dapsone in combination with pyrimethamine (Deltaprim) found no evidence of toxicity [22].If any severe adverse reaction occurs among any enrolled child, the study code will be broken, the medication stopped, the reaction treated, and a note about drug allergy made on the child’s health card. Future episodes of malaria will therefore need to be treated with a different drug. Such reactions will be reported to CDC and KEMRI and to the institutional review boards of both institutions.

A small bruise or mild pain on the finger or the heel from where the blood (maximum 1.5 cc) is taken may develop. There is also a small chance of infection when blood is drawn. There is a small chance of temporary bleeding from the nasal swab.

Alternatives: Parents or guardians may choose not to have their child in the study. Children whose parent or guardian elect not to have their child enrolled in the study will be seen by the regular staff in the clinic. He/she will get the standard MOH care and pay any standard fees. No further visits would be required for these children.

**Adverse Event Reporting.**

Adverse Events: Any unexpected medical occurrence in a participant during the trial will be recorded as an adverse event. There are certain events detailed below which are anticipated to have a baseline rate, and are therefore not unexpected. These events will be reported in a summary statement as part of the yearly continuation. If the study team recognizes an increase above an expected rate, the information will be provided earlier than this annual report.

Because the postneonatal infant mortality rate in the IPTi study area is approximately 120 per 1000 infants, it is anticipated that approximately 170 children will die during the course of the study between enrollment (at 6 weeks of age) and the end of the primary study outcome period (at 12 months of age). Therefore, infant deaths from common causes (including malaria, diarrhea, pneumonia, malnutrition, bacterial sepsis, meningitis, other common childhood infections, injuries, prematurity, and congenital conditions) will be considered anticipated adverse events. Hospitalizations from such illnesses will also be considered expected adverse events. Deaths or hospitalizations from unexpected or unusual causes (examples: allergic reactions, non-viral hepatitis, Stevens-Johnson syndrome, toxic epidermal necrolysis, sudden unexplained death) or that in the judgment of the local PI are possibly or probably related to study drug administration will NOT be consideredexpected adverse events, and will be reported to the CDC as outlined below.

Adverse events that are serious, unexpected, and at least possibly related to study drug:

Adverse events that are serious (that is, results in death, is life-threatening (at the time of the event), requires inpatient hospitalization or prolongation of existing hospitalization, or results in persistent or significant disability), unexpected (meaning previously unobserved or undocumented in humans under the research intervention or one substantially similar), and at least possibly related to study drug administration (meaning the cause of the event is explained at least as well by the research procedures as any other cause) requires prompt reporting to the CDC and KEMRI IRBs. In such cases, initial informal notification of such an event shall occur by e-mail within 2 days of one of the principal investigators becoming aware of the event. Formal reporting (using CDc form 1254 and 1254s (where required) will occur within 2 weeks of the PI becoming aware of the event.

## Data Safety and Monitoring Board

Reporting requirements for the DSMB are slightly different from those for the CDC and KEMRI IRBs

Adverse events are reported to the DSMB based on the following criteria:

- All deaths are reported in one week.
- All hospitalizations from unexpected causes and/or events that could possibly be related to the administration of study drug are reported within one week. Hospitalizations due to critical anemia (Hb <5 g/dL) while not unexpected are nevertheless reported within one week.

All hospitalizations from expected causes are reported in the quarterly report to the DSMB.

* Use of trade names is for identification only, and does not imply endorsement by the Public Health Service or by the U.S. Department of Health and Human Services.

**References**

1. DeMaeyer E, Adiels-Tegman M. The prevalence of anaemia in the world. World Health Statistics Quarterly - Rapport Trimestriel de Statistiques Sanitaires Mondiales 1985;38:302-16

2. Murphy SC, Breman JG. Gaps in the childhood malaria burden in Africa: Cerebral malaria, neurologic sequellae, anemia, repiratory distress, hypoglycemia, and complications of pregnancy. American Journal of Tropical Medicine & Hygiene 2001;64:57-67

3. Shiff C, Checkley W, Winch P, Premji Z, Minjas J and Lubega P. Changes in weight gain and anaemia attributable to malaria in Tanzanian children living under holoendemic conditions. Transactions of the Royal Society of Tropical Medicine & Hygiene 1996;90:262-5

4. McElroy PD, Lal AA, Hawley WA, et al. Analysis of repeated hemoglobin measures in full-term, normal birth weight Kenyan children between birth and four years of age. III. The Asemobo Bay Cohort Project. American Journal of Tropical Medicine & Hygiene 1999;61:932-40

5. Schellenberg D, Menendez C, Kahigwa E, et al. Intermittent treatment for malaria and anaemia control at time of routine vaccinations in Tanzanian infants: a randomised, placebo-controlled trial. Lancet 2001;357:1471-7

6. Breman JG. The ears of the hippopotamus: manifestations, determinants, and estimates of the malaria burden. American Journal of Tropical Medicine & Hygiene 2001;64:1-11

7. Slutsker L, Taylor TE, Wirima JJ and Steketee RW. In-hospital morbidity and mortality due to malaria-associated severe anaemia in two areas of Malawi with different patterns of malaria infection. Transactions of the Royal Society of Tropical Medicine & Hygiene 1994;88:548-51

8. Lozoff B, Brittenham GM, Wolf AW, et al. Iron deficiency anemia and iron therapy effects on infant developmental test performance. Pediatrics 1987;79:981-95

9. Lozoff B, Jimenez E and Wolf AW. Long-term developmental outcome of infants with iron deficiency. New England Journal of Medicine 1991;325:687-94

10. Idjradinata P, Pollitt E. Reversal of developmental delays in iron-deficient anaemic infants treated with iron. Lancet 1993;341:1-4

11. Lawless JW, Latham MC, Stephenson LS, Kinoti SN and Pertet AM. Iron supplementation improves appetite and growth in anemic Kenyan primary school children. Journal of Nutrition 1994;124:645-54

12. Chwang L-C, Soemantrai AG and Pollitt E. Iron supplementation and physical growth of rural Indonesian children. American Journal of Clinical Nutrition 1988;47:496-501

13. Gillespie S, Kevany J and Mason J. Controlling Iron Deficiency. A report of an ACC/SCN Workshop. Geneva: UN ACC/Subcommittee on Nutrition, 1991

14. Kerouedan D, Bontez W, Bondurand A, Abisse S and Konate S. Reflexions sur la transfusion sanguine en Afrique au temps de l'epidemie de SIDA. Etat des lieux et perspectives en Cote-d'lvoire. Sante 1994;4:37-42

15. Bloland PB, Boriga DA, Ruebush TK, et al. Longitudinal cohort study of the epidemiology of malaria infections in an area of intense malaria transmission II. Descriptive epidemiology of malaria infection and disease among children. American Journal of Tropical Medicine & Hygiene 1999;60:641-8

16. Campbell CC. Challenges facing antimalarial therapy in Africa. Journal of Infectious Diseases 1991;163:1207-11

17. Kidane G, Morrow RH. Teaching mothers to provide home treatment of malaria in Tigray, Ethiopia: a randomised trial. Lancet 2000;356:550-5

18. Spencer HC, Kaseje DC, Mosley WH, Sempebwa EK, Huong AY and Roberts JM. Impact on mortality and fertility of a community-based malaria control programme in Saradidi, Kenya. Annals of Tropical Medicine & Parasitology 1987;81 Suppl 1:36-45

19. Greenwood BM, Greenwood AM, Bradley AK, et al. Comparison of two strategies for control of malaria within a primary health care programme in the Gambia. Lancet 1988;1:1121-7

20. Menon A, Snow RW, Byass P, Greenwood BM, Hayes RJ and N'Jie AB. Sustained protection against mortality and morbidity from malaria in rural Gambian children by chemoprophylaxis given by village health workers. Transactions of the Royal Society of Tropical Medicine & Hygiene 1990;84:768-72

21. Greenwood BM, David PH, Otoo-Forbes LN, et al. Mortality and morbidity from malaria after stopping malaria chemoprophylaxis. Transactions of the Royal Society of Tropical Medicine & Hygiene 1995;89:629-33

22. Menendez C, Kahigwa E, Hirt R, et al. Randomised placebo-controlled trial of iron supplementation and malaria chemoprophylaxis for prevention of severe anaemia and malaria in Tanzanian infants. Lancet 1997;350:844-50

23. Watkins WM, Brandling-Bennett AD, Nevill CG, et al. Chlorproguanil/dapsone for the treatment of non-severe *Plasmodium falciparum* malaria in Kenya: a pilot study. Transactions of the Royal Society of Tropical Medicine & Hygiene 1988;82:398-403

24. Wilairatana P, Kyle DE, Looareesuwan S, et al. Poor efficacy of antimalarial biguanide-dapsone combinations in the treatment of acute, uncomplicated, falciparum malaria in Thailand. Annals of Tropical Medicine & Parasitology 1997;91:125-32

25. Amukoye E, Winstanley PA, Watkins WM, et al. Chlorproguanil-dapsone: effective treatment for uncomplicated falciparum malaria. Antimicrobial Agents & Chemotherapy 1997;41:2261-4

26. Keuter M, van Eijk A, Hoogstrate M, et al. Comparison of chloroquine, pyrimethamine and sulfadoxine, and chlorproguanil and dapsone as treatment for falciparum malaria in pregnant and non-pregnant women, Kakamega District, Kenya. BMJ 1990;301:466-470

27. Nzila AM, Nduati E, Mberu EK, et al. Molecular evidence of greater selective pressure for drug resistance exerted by the long-acting antifolate pyrimethamine/sulfadoxine compared with the shorter-acting chlorproguanil/dapsone on Kenyan *Plasmodium falciparum*. Journal of Infectious Diseases 2000;181:2023-2028

28. Curtis J, Duraisingh MT and Warhurst DC. In vivo selection for a specific genotype of dihydropteroate synthetase of *Plasmodium falciparum* by pyrimethamine-sulfadoxine but not chlorproguanil-dapsone treatment. Journal of Infectious Diseases 1998;177:1429-33

29. Neftel KA, Woodtly W, Schmid M, Frick PG and Fehr J. Amodiaquine induced agranulocytosis and liver damage. British Medical Journal 1986;292:721-723

30. Hatton CSR, Peto TEA, Bunch C, et al. Frequency of severe neutropenia associated with amodiaquine prophylaxis against malaria. Lancet 1986;1(8478):411-414

31. Larrey D, Castot A, Pessayre D, et al. Amodiaquine-induced hepatitis. A report of seven cases. Annals of Internal Medicine 1986;104:801-803

32. Bernuau J, Larrey D, Campillo B, et al. Amodiaquine-induced fulminant hepatitis. Journal of Hepatology 1988;6:109-112

33. Sturchler D, Schar M and Gyr N. Leucopenia and abnormal liver function in travellers on malaria chemoprophylaxis. Journal of Tropical Medicine and Hygiene 1987;90:239-243

34. Olliaro P, Mussano P. Amodiaquine for treating malaria. Cochrane Database of Systematic Reviews 2000:CD000016

35. Adjuik M, Agnamey P, Babiker A, et al. Amodiaquine-artesunate versus amodiaquine for uncomplicated *Plasmodium falciparum* malaria in African children: a randomised, multicentre trial. Lancet 2002;359:1365-1372

36. Schellenberg D, Kahigwa E, Drakeley C, et al. The safety and efficacy of sulfadoxine-pyrimethamine, amodiaquine, and their combination in the treatment of uncomplicated *Plasmodium falciparum* malaria. American Journal of Tropical Medicine and Hygiene 2002;67:17-23

37. Brasseur P, Guiguemde R, Diallo S, et al. Amodiaquine remains effective for treating uncomplicated malaria in west and central Africa. Transactions of the Royal Society of Tropical Medicine and Hygiene 1999;93:645-650

38. Olliaro P, Nevill C, LeBras J, et al. Systematic review of amodiaquine treatment in uncomplicated malaria. Lancet 1996;348:1196-1201

39. Massaga J, Kitua A, Lemnge M, et al. Amodiaquine intermittent treatment reduces the incidence of anaemia and malaria fevers during the first year of life in a district of Tanzania highly endemic for drug resistant malaria. In: Third MIM Pan-African Malaria Conference. Arusha, Tanzania, 2002

40. Gupta S, Thapar MM, Mariga ST, Wernsdorfer WH and Bjorkman A. *Plasmodium falciparum*: in vitro interactions of artemisinin with amodiaquine, pyronaridine, and chloroquine. Experimental Parasitology 2002;100:28-35

41. Doherty JF, Sadiq AD, Bayo L, et al. A randomized safety and tolerability trial of artesunate plus sulfadoxine--pyrimethamine versus sulfadoxine-pyrimethamine alone for the treatment of uncomplicated malaria in Gambian children. Transactions of the Royal Society of Tropical Medicine & Hygiene 1999;93:543-6

42. von Seidlein L, Milligan P, Pinder M, et al. Efficacy of artesunate plus pyrimethamine-sulphadoxine for uncomplicated malaria in Gambian children: a double-blind, randomised, controlled trial. Lancet 2000;355:352-7

43. Targett G, Drakeley C, Jawara M, et al. Artesunate reduces but does not prevent posttreatment transmission of Plasmodium falciparum to *Anopheles gambiae*. Journal of Infectious Diseases 2001;183:1254-9

44. Prescribing information: Fansidar brand of sulfadoxine and pyrimethamine tablets. Nutley, New Jersey: Roche Pharmaceuticals, 1993

45. Winstanley P, Watkins W, Muhia D, Szwandt S, Amukoye E and Marsh K. Chlorproguanil/dapsone for uncomplicated *Plasmodium falciparum* malaria in young children: pharmacokinetics and therapeutic range. Transactions of the Royal Society of Tropical Medicine & Hygiene 1997;91:322-7

46. The use of antimalarial drugs: report of a WHO informal consultation. Geneva, Switzeralnd: World Health Organization, 2001

47. Beier JC, Perkins PV, Onyango FK, et al. Characterization of malaria transmission by Anopheles (Diptera: Culicidae) in western Kenya in preparation for malaria vaccine trials. Journal of Medical Entomology 1990;27:570-577

48. McElroy PD, ter Kuile F, Hightower AW, et al. All-cause mortality among young children in western Kenya. IV. The Asembo Bay cohort project. American Journal of Tropical Medicine & Hygiene 2001;64:18-27

49. Kenya Demographic and Health Survey 1998. Calverton, Maryland: Macro International, Inc., 1999

50. Matthews JNS. An Introduction to Randomized Controlled Clinical Trials. London: Arnold, 2000

51. Miller KD, Lobel HO, Satriale RF, Kuritsky JN, Stern R and Campbell CC. Severe cutaneous reactions among American travelers using pyrimethamine-sulfadoxine (Fansidar) for malaria prophylaxis. American Journal of Tropical Medicine and Hygiene 1986;35:451-458

52. Bruce-Chwatt LJ, Hutchinson DB. Maloprim and agranulocytosis. Lancet 1983;2(8365-66):1487-1487
