# Supplementary figures and images for: Intermittent Preventive Treatment in Infants for the Prevention of Malaria in Rural Western Kenya: A Randomized, Double-Blind Placebo-Controlled Trial
Source: PLoS One. 2010 Apr 2;5(4):e10016. doi: 10.1371/journal.pone.0010016 (PMC2848869; doi:10.1371/journal.pone.0010016)

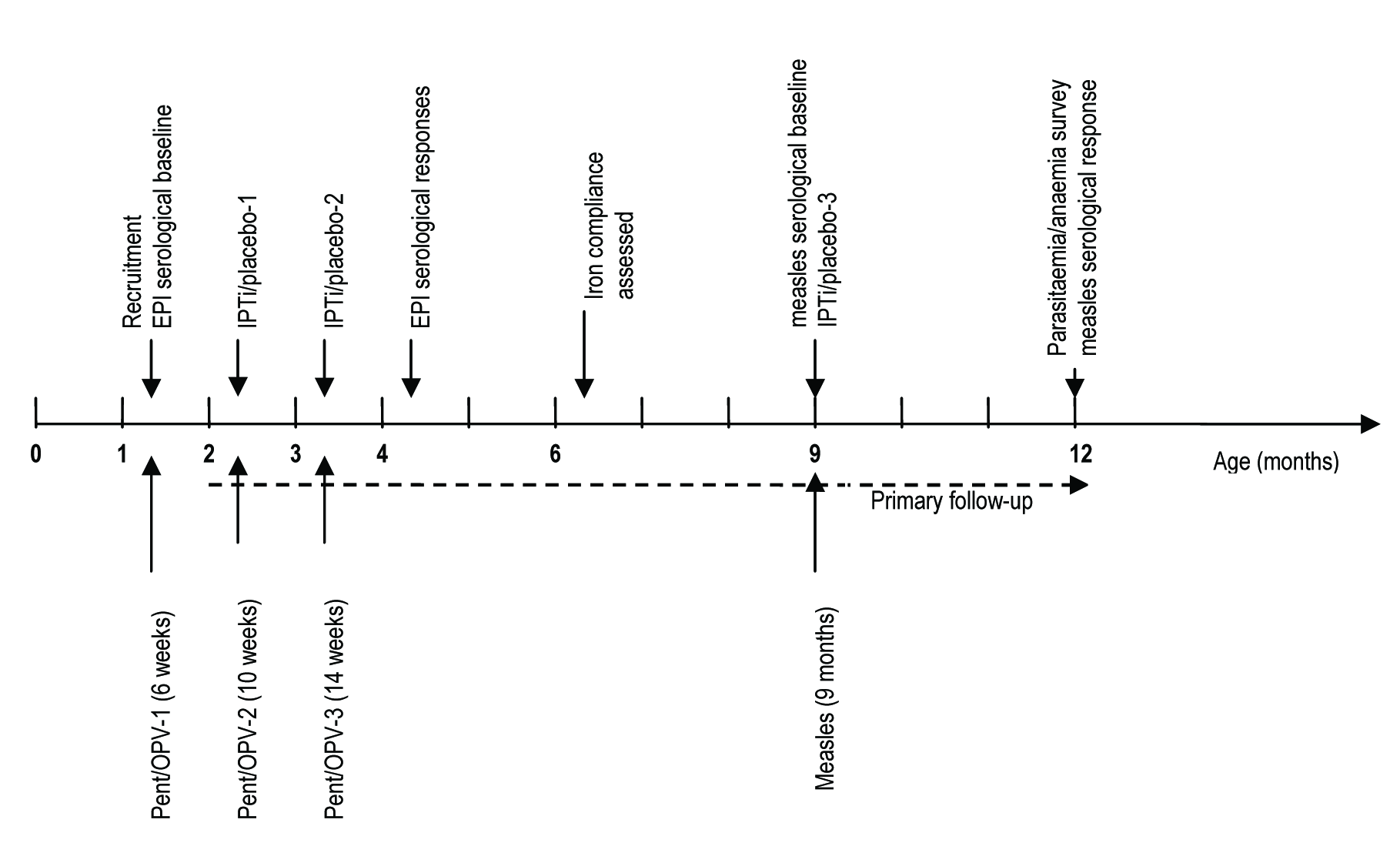

Supplement: Figure S1 — Trial time-line for participants of the IPTi trial in western Kenya Note: PENT = diphtheria-tetanus toxoid-pertussis-hepatitis B-Haemophilus influenza type b vaccine; OPV = oral polio vaccine; EPI = Expanded Programme of Immunization; IPTi = Intermittent Preventive Treatment of infants. (0.68 MB TIF) [file pone.0010016.s004.tif]

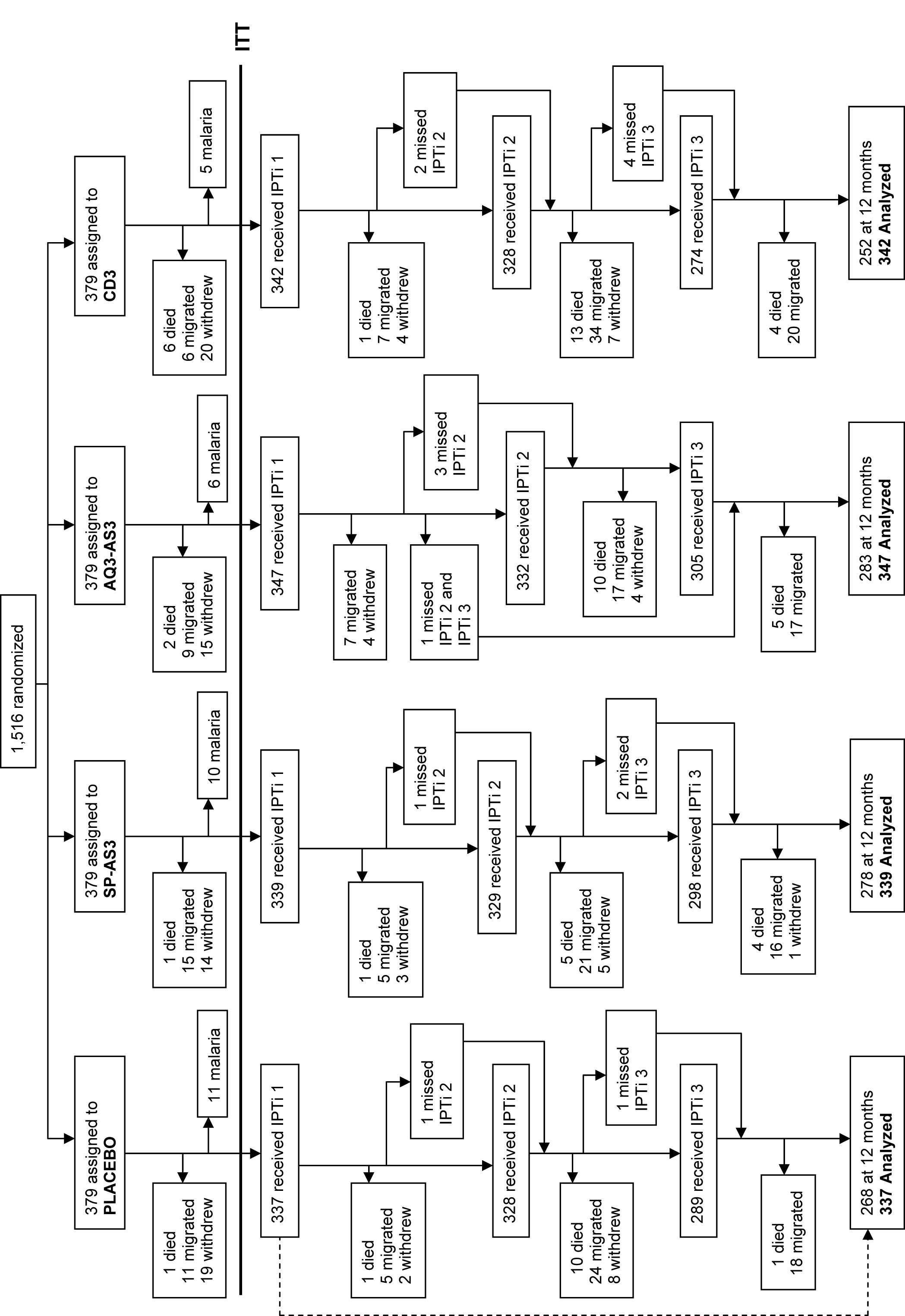

Supplement: Figure S2 — Trial profile Flowchart (0.43 MB TIF) [file pone.0010016.s005.tif]

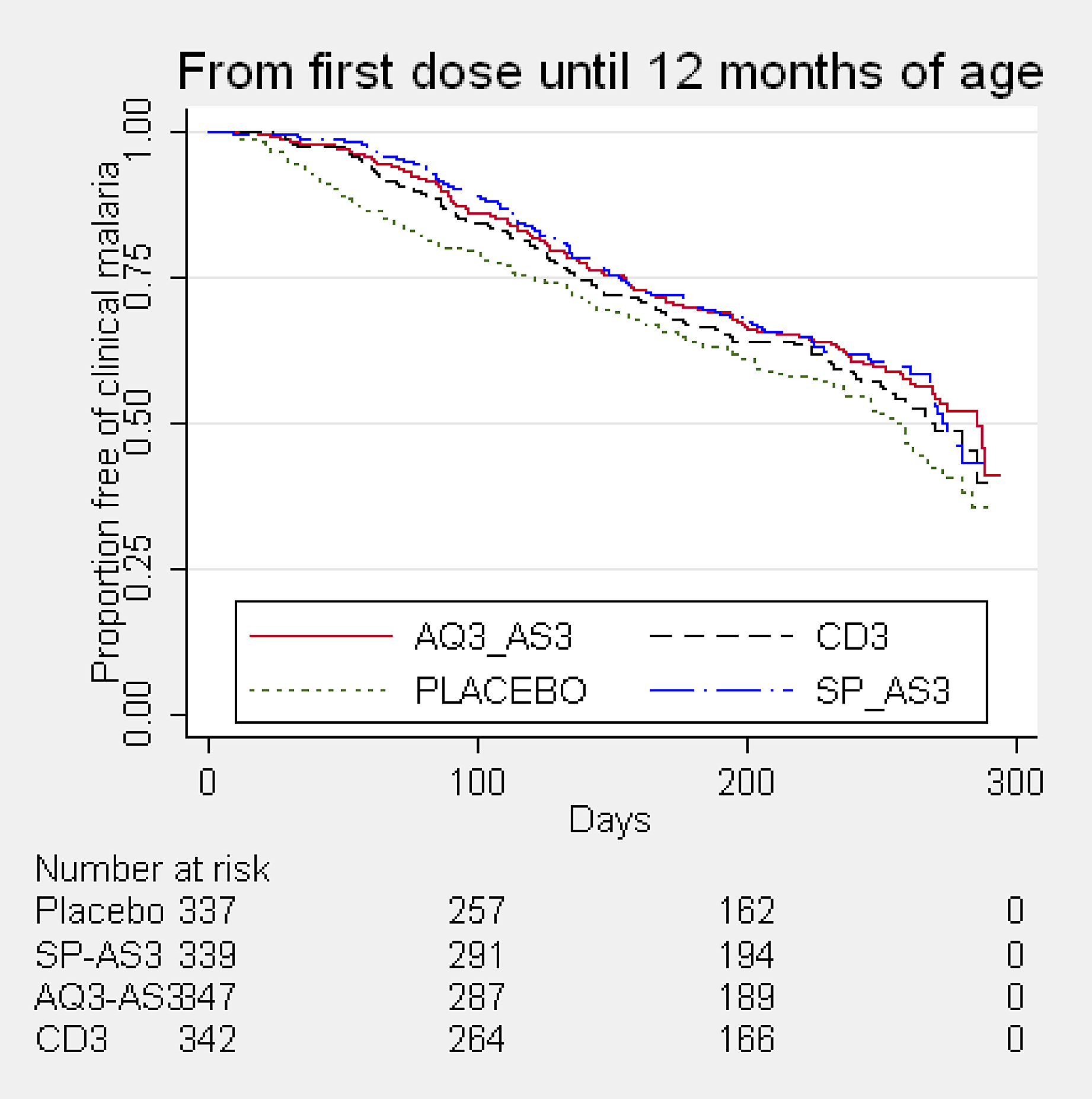

Supplement: Figure S3 — Kaplan-Meier plots showing the cumulative proportion of children remaining free of clinical malaria episodes between the first dose of IPTi and 12 months of age. (0.31 MB TIF) [file pone.0010016.s006.tif]

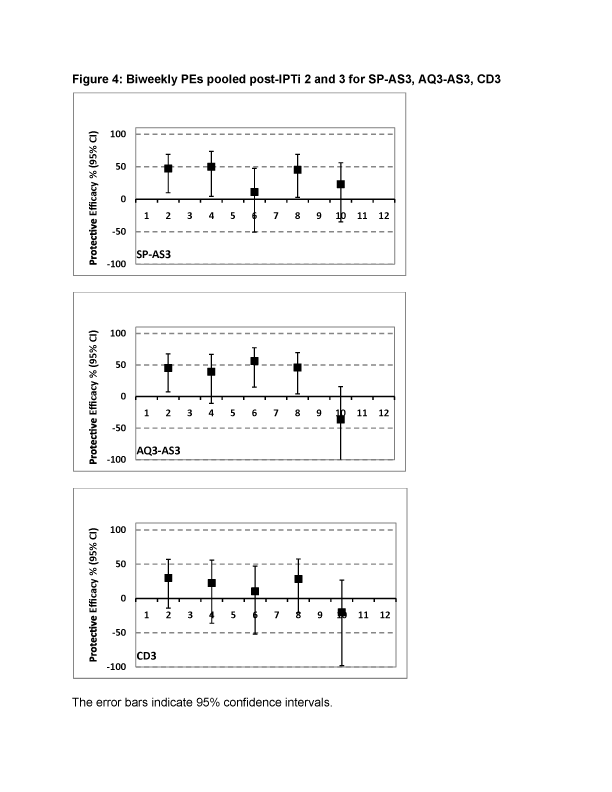

Supplement: Figure S4 — Biweekly PEs pooled post-IPTi 2 and 3 for SP-AS3, AQ3-AS3, and CD3. Note: the error bars indicate 95% confidence intervals. (0.06 MB TIF) [file pone.0010016.s007.tif]
