# Supplementary material for: Intermittent Preventive Treatment in Infants for the Prevention of Malaria in Rural Western Kenya: A Randomized, Double-Blind Placebo-Controlled Trial
Source: PLoS One. 2010 Apr 2;5(4):e10016. doi: 10.1371/journal.pone.0010016 (PMC2848869; doi:10.1371/journal.pone.0010016)
Supplement: Consent S1 — Parental/Guardian consent form. (0.05 MB DOC) [file pone.0010016.s008.doc]

Flesch-Kincaid Reading Score: 8.1

**Parental/Guardian Permission form – Malaria and Anemia Prevention Study (IPTi)**

Background: The Kenya Medical Research Institute (KEMRI) and the Centers for Disease Control and Prevention, USA (CDC) are doing a research study to find the best prevention for lack of blood (anemia) and malaria. As part of this study we want to compare different ways to prevent lack of blood and malaria. One drug we are giving children is iron. The other drugs are Fansidar® with artesunate, amodiaquine with artesunate, and Lapdap®. All three of these drug regimens are used to treat malaria. You can buy iron, amodiaquine, and Fansidar in many shops in Asembo. Artesunate and Lapdap are not widely sold. We want to know if Fansidar + artesunate, amodiaquine + artesunate, or Lapdap, when given with iron, is better than iron alone for preventing lack of blood in infants. We also would like to know if these drugs change the way the body responds to vaccines. About 1500 infants will be enrolled in the study.

Treatment groups: As part of this study, all children who join the study will receive treatment with iron for 4 months. Children will also receive one of four different types of pills. One group of children will get a crushed sugar pill with no medicine (placebo). One group will get Fansidar + artesunate. One group will get amodiaquine + artesunate. One group will get Lapdap. Children in each of the groups will receive crushed pills in syrup at 10 weeks of age, 14 weeks of age, and 9 months of age. Some pills will be taken in clinic, with another dose the next day and a third dose the day after that. Someone will come to your house to give the drug on the second and third day. Neither the study team nor you can pick the treatment group as this could affect the study results. The treatment will be chosen at random (like flipping a coin). Neither the study team nor you will know which of the drugs your child is receiving until the study is over.

Follow-up Visits: As part of this study, you will bring your child to this clinic a total of 7 additional times during the study (at 10, 14 & 18 weeks, and at 9, 12, 18 and 24 months of age). At each visit a health worker will ask questions about your child’s health and examine the child. At some visits, the study staff will take the weight and height of the child. At some visits, she will also prick your child’s heel or finger to obtain a blood sample. At some visits, your child will be given the study drug. Each visit should take about 30 minutes.

Study drugs: At the 10-week, 14-week, and 9-month vaccine visit, your child will get medication to take in clinic. A study team member will visit your house the next two days to give the 2 other doses of medicine. Each time your child is given a study drug, our study staff will watch the child for 30 minutes to make sure he does not vomit the drug or become ill. You will also get a bottle with medicine (iron) at 10 weeks, and again at 14 & 18 weeks. We would like you to give this medicine to your child every day. When your child is 6 months old, we will visit your house to check to see if your child has been given all the iron.

Blood samples: We will prick your child’s heel or finger to obtain a blood sample today, and again at 10 weeks, 18 weeks, 9 months and 12 months of age. The amount of blood we will take is small, about ¼ of a teaspoon. The blood will be stored. Some of the blood will be used to see whether your child had an adequate response to the vaccines. Some of the blood will be used to test for a condition in the blood called sickle cell. You will be informed if your child has sickle cell disease. We will provide counseling to help you understand how to seek medical care for your child and how to care for your child at home. Some of the blood will be used to test for other traits that may cause anemia in children, such as G6PD deficiency.

At the 10 week and 12 month visits, we will put a few drops of your child’s blood onto a small piece of paper. We will test the blood on this paper to learn more about how well the drugs that are used to treat malaria are working in your community.

At 12, 18 and 24 months, we will take 5 drops of blood to check if your child has malaria and/or anemia (lack of blood). We will provide treatment if your child has anemia or malaria and a history of hot body at these visits.

Nasal swab: At the visit at 9 months of age, we will use a cotton swab to go inside your child’s nose. We will check to see whether your child is carrying any bacteria that cause acute upper respiratory disease. All children are vaccinated against this disease, so even if your child has this bacteria, it does not mean that he or she is sick. We are testing to see whether your child has an adequate response to the vaccine.

Sick visits: We ask you to bring your child to this clinic if your child is ill at any time during the study. In the event of an emergency, you should bring your child to the nearest health facility or hospital. We also have staff in the following health clinics: Abidha, Ongi’elo, Lwak, and Saradidi. In case of illness after the clinic closes,at night**,** or on the weekend, we will have a nurse on-call at St. Elizabeth’s Lwak Hospital to see your child. If your child requires hospitalization, we will provide transport for the child to be taken to New Nyanza General Provincial Hospital. We will pay for the child’s hospitalization at these hospitals for malaria, lack of blood, worms, diarrhea, and pneumonia illnesses.

We would prefer you not to buy any routine treatment for malaria or treatment for lack of blood from shops or clinics during the study unless necessary. We ask this because extra medicine may result in side effects for your child. Other drugs may also reduce the effect of the study drugs. We supply all other drugs for malaria, lack of blood, worms, diarrhea, and pneumonia free of charge at the health clinic. Therefore, it will usually not be necessary to buy other medicines for those conditions.

Risks: All medicine has side effects.

*General:* There is some chance of vomiting after any of the study drugs. If this occurs within 30 minutes, then your child will be given another dose of the drug. If the vomiting occurs later than 30 minutes, no additional drug will be given.

*Iron:* Some children do not like the taste of iron. Some children complain of nausea and not feeling like eating after taking iron. In some children the teeth will become darker. In other children the stool will become darker or greener than normal. This is a normal reaction to treatment with iron. The teeth and stool color will become normal again after the iron treatment is completed. Iron is also toxic if taken in large doses (more than recommended amount).

*Fansidar:* Sometimes a child may get a rash after treatment with Fansidar®, one of the study medicines. If this occurs, we will stop the medicine and recommend that your child be treated with different malaria medicines in the future.

*Amodiaquine*: There are no known specific risks of amodiaquine when taken intermittently to treat malaria.

*Artesunate:* There are no known specific risks of artesunate.

*Lapdap:* Sometimes a child can get a temporary anemia following Lapdap. We believe this risk to be small. However, as this is a newer drug, and has not been used often in children in Africa, the risks are not completely known. .

*Sugar pills:* We expect no side effects with sugar pills.

*Heel or finger prick:* A small bruise or mild pain on the heel or finger from where the blood is taken may develop. There is also a chance of infection when blood is drawn. This chance is very small because we always use clean materials.

*Nasal swab:* There is a chance of local irritation, bleeding, or infection from the nasal swab.

Costs/compensation: If the event of any problem from the heel or finger prick or the nasal swab that a doctor needs to look at, or a severe reaction to the malaria drug treatment, we will provide transport to the hospital. In the hospital your child will be treated free of charge. No such incident has occurred since the beginning of the CDC studies in Asembo since June 1992.

Lab tests: All blood samples will be tested in Kisian for malaria. Part of the blood and the nasal swab may be stored and sent to labs in Europe and the USA. In the USA, further studies may be done that will help us to know more about how your body protects itself against malaria and lack of blood. This includes a study of how your child’s body responds to vaccines. The nasal swab will be tested for bacteria. The blood may be tested for iron, response to iron, or response to malaria. Part of the blood will be sent to France to study your child’s response to vaccines. If we get any results from these studies that may affect the health of your child, we will inform you. Results of the G6PD test will not be given. All the lab tests will be done free of charge.

Privacy: If you want your child’s blood sample taken out of storage, please let our study staff know. We will destroy the sample. This will have no effects for you or your child. The facts we collect in the study and the results of the lab tests will be kept private to the extent allowed by law. The child’s name will not be used on any of the study reports or study samples. Only study personnel will have access to these records and samples.

Severe illness: This study involves children who are healthy. Children with severe illness today will not be included in this study but referred to one of the hospitals in the study area. Also if your child becomes very ill during the study because he has had a severe reaction to one of the study drugs we have given your child your child’s participation in the study will need to be stopped.

Withdrawal from the study: Some children may be prescribed daily treatment with cotrimoxasole by a clinician. Cotimoxasole may cause a serious reaction with one of our study drugs. Therefore, if your child begins taking daily cotrimoxasole, we will have to withdraw him or her from the study. This withdrawal will be done to safeguard the health of your child.

Stopping the study: You are free to choose for your child to be part of this study. If you do not want your child to be in the study, you have the right to refuse. If you do not want your child to go on with this study you can also stop anytime. This will have no effects for you or your child. You and your child can continue to receive regular care from this clinic.

Benefits: Your child will likely benefit from being in this study. Your child will receive free health care for malaria, lack of blood, worms, diarrhea, and pneumonia illnesses for a period of 24 months starting from the first day of this study. You will be given a “health passport” which allows your child to receive free treatment for these conditions in one of the study clinics for this period. The child will be seen by a special study nurse, which may result in shorter wait times for medical care. Inpatient hospital costs at Lwak and New Nyanza General Provincial hospitals will also be covered for enrolled children for medical conditions listed in sick visits section. Any other medical costs (such as specialty care or hospitalization for conditions not listed above or not resulting from one of the study medications) are not covered by the project. The free treatment and care at the hospital will help improve the health of your child. The free iron your child receives will probably help prevent lack of blood. Because your child will visit the clinic regularly during the course of the study, your child is also likely to have malaria detected and treated more quickly than usual. Your child will also be receiving malaria tests for febrile illnesses, which may allow for better treatment of these illnesses. You will be informed if your child has sickle cell disease, which may help you seek needed care. We hope the results of this study will help to improve the treatment for malaria and lack of blood in this area.

Alternatives: You can choose not to have your child in this study. If you choose not to have your child in the study, your child will be seen by the regular staff in this clinic. He/she will get the standard MOH care and pay any standard fees. No further visits would be required.

Contacts: If your child has any problems from the study that you want a doctor to look at, you can contact Dr. Juliana Otieno at New Nyanza Provincial General Hospital. The telephone number is 23200. Should any further questions arise, or if you want to quit the study, please contact Dr. Larry Slutsker, Mr. Peter Otieno or Mr. Frank Odhiambo, at the CDC office in Kisian. The office is at Kisian, on the Kisumu-Busia Road. The telephone number is 22929, 22959, or 22983. If you have any questions about your rights as a study patient, or if you want to talk about the study with someone who is not part of this research project, please contact Dr. Margaret Oloo. Dr. Oloo is a special doctor for children and is not part of the study. You can also contact Dr. Margaret Oloo if you think your child has been injured because of this study. She works in the Aga Khan Hospital in Kisumu and can be reached by phone. The phone number is 057 41031.

Thank you very much for your time.

| **Malaria and Anemia Prevention Study:** | |
| --- | --- |
| The above has been explained to me and I have read the permission form or it has been read to me. I agree for my child to take part in the study. I have been told that I am free to choose for my child to be part of this study. I also have been told that if I do not want my child to go on with this study I can stop at anytime. This will have no effect on future care that I might receive for my child or me. I agree for my child’s blood to be tested for malaria, lack of blood and factors that may protect against malaria, lack of blood, or bacterial infections. I agree for my child’s nasal swab to be tested for bacteria. |  |

| Parent/Guardian | Name…………………..  Initials………….. | Signature…………………….  Mark* | Date………………… |
| --- | --- | --- | --- |
| IPTi Registrar | Name………………….. | Signature……………………. | Date………………… |
| Independent witness* | Name………………….. | Signature…………………….  Mark** | Date………………… |
| **A parent can sign or make a mark and have his/her permission confirmed by the signature of a witness.*  *** A mark is made by the parent or guardian by thumbprint (placing their thumb on an inkpad and pressing down in the space above)* | | | |

**Either mother or father or guardian can sign. All not required. Witness only required for those who cannot sign. All pages of the consent form must be initialed by the mother, father, or guardian (the person who signs the consent form).**

| **Long-term storage and future studies:** | |
| --- | --- |
| We would like to store your child’s leftover blood to do more tests related to malaria in the future. No HIV or genetic testing is planned. It is your choice to let us keep the blood after the study ends. You can still be part of the study if you do not want us to store the blood. At the end of this study, we will take your name off of all forms. Therefore, we cannot report any new test results on the stored blood to you.  If you change your mind about saving blood while the study is ongoing, we can destroy the blood. To do this, contact Mr. Peter Otieno, Mr Frank Odhiambo, or Dr. Mary Hamel at the CDC office in Kisian. The office is at Kisian, on the Kisumu-Busia Road. The telephone number is 22929, 22959, or 22983 |  |

| Parent/Guardian | Name…………………..  Initials………….. | Signature…………………….  Mark* | Date………………… |
| --- | --- | --- | --- |
| IPTi Registrar | Name………………….. | Signature……………………. | Date………………… |
| Independent witness* | Name………………….. | Signature…………………….  Mark** | Date………………… |
| **A parent can sign or make a mark and have his/her permission confirmed by the signature of a witness.*  *** A mark is made by the parent or guardian by thumbprint (placing their thumb on an inkpad and pressing down in the space above)* | | | |
